# The spider family Selenopidae (Arachnida, Araneae) in Australasia and the Oriental Region

**DOI:** 10.3897/zookeys.99.723

**Published:** 2011-05-19

**Authors:** Sarah C. Crews, Mark S. Harvey

**Affiliations:** 1 Department of Terrestrial Zoology, Western Australian Museum, Locked Bag 49, Welshpool DC, Perth, WA 6986, Australia; 2 School of Animal Biology, University of Western Australia, 35 Stirling Highway, Crawley, Perth, WA 6009, Australia; 3 Research Associate, Division of Invertebrate Zoology, American Museum of Natural History, New York, NY 10024, USA; 4 Research Associate, California Academy of Sciences, 55 Music Concourse Drive, Golden Gate Park, San Francisco, CA 94118, USA

**Keywords:** Selenopidae, morphology, new species, endemism, Australasia, Oriental Region, taxonomy, new genera, systematics, biodiversity, *Selenops*, *Anyphops*, *Garcorops*, *Hovops*, *Siamspinops*

## Abstract

We relimit and revise the family Selenopidae to include four new genera and 27 new species from Australia and the Oriental Region. The family is redefined, as are the genera *Anyphops* Benoit, *Garcorops* Corronca, *Hovops* Benoit, *Selenops* Latreille, and *Siamspinops* Dankittipakul & Corronca, to accommodate the new genera and to correct previous inconsistencies in the diagnoses and definitions of the aforementioned genera. The species of *Selenops* that occur throughout India and China are also reviewed. Three species occur in China: *Selenops bursarius* Karsch 1879, also known from Japan, Korea and Taiwan, *Selenops ollarius* Zhu, Sha, & Chen 1990, and *Selenops radiatus* Latreille 1819, the type of the genus and most widespread selenopid. *Selenops cordatus* Zhu, Sha & Chen **syn. n.** is recognized as a junior synonym of *Selenops radiatus*. *Amamanganops* **gen. n.** is monotypic, with *Amamanganops baginawa* **sp. n.** (♀; from the Philippines). *Godumops* **gen. n.** is monotypic, with *Godumops caritus* **sp. n.** (♂; from Papua New Guinea). *Karaops* **gen. n.** occurs throughout Australia and includes 24 species. A new combination is proposed for *Karaops australiensis* (L. Koch 1875) **comb. n.** (ex. *Selenops*), and the new species: *Karaops gangarie* **sp. n.** (♀, ♂), *Karaops monteithi* **sp. n.** (♀), *Karaops alanlongbottomi* **sp. n.** (♂), *Karaops keithlongbottomi* **sp. n.** (♂), *Karaops larryoo* **sp. n.** (♂), *Karaops jarrit* **sp. n.** (♂,♀), *Karaops marrayagong* **sp. n.** (♀), *Karaops raveni* **sp. n.** (♂,♀), *Karaops badgeradda* **sp. n.** (♀), *Karaops burbidgei* **sp. n.** (♂,♀), *Karaops karrawarla* **sp. n.** (♂,♀), *Karaops julianneae* **sp. n.** (♀), *Karaops martamarta* **sp. n.** (♀), *Karaops manaayn* **sp. n.** (♀, ♂), *Karaops vadlaadambara* **sp. n.** (♀, ♂), *Karaops pilkingtoni* **sp. n.** (♀, ♂), *Karaops deserticola* **sp. n.** (♀), *Karaops ngarutjaranya* **sp. n.** (♂,♀), *Karaops francesae* **sp. n.** (♂,♀), *Karaops toolbrunup* **sp. n.** (♀, ♂), the type species *Karaops ellenae* **sp. n.** (♂,♀), *Karaops jenniferae* **sp. n.** (♀), and *Karaops dawara* **sp. n.** (♀).The genus *Makdiops* **gen. n.** contains five species from India and Nepal. A new combination is proposed for *Makdiops agumbensis* (Tikader 1969), **comb. n.**, *Makdiops montigenus* (Simon 1889), **comb. n.**, *Makdiops nilgirensis* (Reimoser 1934) **comb. n.**,(ex. *Selenops*). Also, there are two new species the type of the genus *Makdiops mahishasura* **sp. n.** (♀; from India), and *Makdiops shiva* **sp. n.** (♀). The genus *Pakawops* **gen. n.** is monotypic. A new combination is proposed for *Pakawops formosanus* (Kayashima 1943) **comb. n.** (ex. *Selenops*), known only from Taiwan. A new combination is proposed for *Siamspinops aculeatus* (Simon)**comb. n**. (ex. *Selenops*). The distribution and diversity of the studied selenopid fauna is discussed. Finally, keys are provided to all of the selenopid genera and to the species of *Karaops* **gen. n.**and *Makdiops* **gen. n.**

## Introduction

Spiders of the family Selenopidae are distributed in the tropical and subtropical regions worldwide. Also known as wall crab spiders or flatties, they are exceptional in that both their running and striking speeds place them amongst the world’s fastest animals (Crews et al. 2008; Crews unpubl. data), and they are also extremely flattened dorsoventrally. The family currently comprises around 200 species in five genera (Corronca 1998; Platnick 2010): *Anyphops* Benoit 1968, *Garcorops* Corronca 2003, *Hovops* Benoit 1968, *Siamspinops* Dankittipakul & Corronca 2009, and *Selenops* Latreille 1819, the first three being distributed in Africa and Madagascar, *Siamspinops* in Southeast Asia, and *Selenops* as currently defined, is distributed worldwide.

Revisionary and descriptive work for *Anyphops*, *Hovops* and *Garcorops* has been achieved by Lawrence (1940), Benoit (1968), and, more recently, by (Corronca (1996, 2000, 2003, 2005). The genus *Selenops* has been revised primarily by region, with the African species examined by (Lawrence (1940, 1942), Benoit (1968), and (Corronca (2001, 2002), and the South American species revised by (Corronca (1996, 1998). In the West Indies and Central America, the last major revisionary work was conducted by Muma (1953), while (Alayón-García (1992, 2001, 2003, 2005) has revised the species in Cuba and completed other regional descriptions, while only a few species have been described from México (Valdez-Mondragón 2007, 2010) since Muma’s work (1953). Recently, Crews (in press) has undertaken a revision of the species of *Selenops* from North America, Central America and the West Indies, excluding Cuban endemics, resulting in 66 species for this region.

While it has long been known that species of Selenopidae occur in Asia and Australia (e.g., Karsch 1879; L. Koch 1875; Simon 1889a, 1901), the only recent work comes from Dankittipakul and Corronca (2009), in which a new genus, *Siamspinops*, is described, as well as several species from Southeast Asia. A handful of species, all placed in the genus *Selenops*, have also been described from various locations in Asia including India, China, Japan and Taiwan (Karsch 1879; Simon 1889b, 1901; Gravely 1931; Reimoser 1934; Kayashima 1943a, b; Tikader 1969; Patel and Patel 1973; Zhu et al. 1990), however, based on the dearth of specimens in museums, it is clear that more collecting from this region is needed.

Although the Australian fauna is restricted to a single species, *Selenops australiensis* L. Koch from northeastern Queensland (L. Koch 1875), a wide variety of new species have been recently detected amongst museum collections. These taxa represent a previously unreported spider radiation from mainland Australia.

The purpose of this paper is twofold. First, we focus on the genera of the family Selenopidae. This includes providing more solid definitions of each genus, describing five new genera, and providing a key to the genera of the family Selenopidae. Second, we provide a revision of the species of Selenopidae from Australasia, describing 27 new species. A key to the Australian species of *Karaops* gen. n.and a key to the new Asian genus *Makdiops* gen. n. are also provided.

## Materials and methods

Taxa were described and illustrated primarily from specimens stored in 75% ethyl alcohol. In some cases, specimens were not available for direct examination, and we based our conclusions on published descriptions. Female copulatory organs were dissected and cleared using pancreatin (Álvarez-Padilla and Hormiga 2008). Digital images were prepared using a Leica DFC 500 attached to a LEICA MZ16A microscope and the software program AutoMontage Pro Version 5.02 (p) by Syncroscopy. Illustrations were prepared directly from the specimen or from digital images. The left palpus was illustrated, or if unavailable, a photograph of the right palpus was reversed. Positions on the face of the left bulb oriented in a standard ‘upright’ position are denoted by referring to a clock dial. Measurements were made using an ocular micrometer or from digital images, and are given in millimeters. Leg spination patterns follow that of Platnick and Shadab (1975). When a ventral leg spination pattern is given for the tibiae and metatarsi, such as 4–3, it means there are 4 pairs of ventral spines on the tibiae and 3 pairs of ventral spines on the metatarsi. Somatic characters are as defined in Ubick et al. (2005), and genitalic characters for selenopids follow that of (Corronca (1998, 2002, 2003, 2005), except for the posterodorsal fold which Corronca called the uterus externus (see Crews, in press).

### Abbreviations used in the text are as follows:

**Eyes**

AER anterior eye row

ALE anterior lateral eyes

AME anterior median eyes

PER posterior eye row

PLE posterior lateral eyes

PME posterior median eyes

**Legs and palps**

Fm femur

Mt metatarsus

Pt patella

Ti tibia

Ta tarsus

**Leg spination**

ap apical

d dorsal

pr prolateral

rt retrolateral

v ventral

**Male copulatory organs**

MA median apophysis

RTA retrolateral tibial apophysis

C conductor

**Repositories**

AM Australian Museum, Sydney, NSW, Australia (G. Milledge, H. Smith)

CAS California Academy of Sciences, San Francisco, CA, USA (C. Griswold, D. Ubick)

MHNG Muséum d’Histoire Naturelle, Geneva, Switzerland (P. Schwendinger)

QM Queensland Museum, Brisbane, Qld, Australia (R. Raven, O. Seeman)

RMCA Royal Museum for Central Africa, Tervuren, Belgium (D. DeBakker, R. Jocqué)

SAM South Australian Museum, Adelaide, SA, Australia (D. Hirst)

UMZC University Museum of Zoology, Cambridge University, United Kingdom (M. Lowe)

WAM Western Australian Museum, Welshpool, WA, Australia (J. Waldock)

ZMB Museum für Naturkunde, Berlin, Germany (J. Dunlop)

ZMH Zoologisches Institut und Zoologisches Museum, Hamburg, Germany (H. Dastych)

ZMUM Zoological Museum of the Moscow University, Moscow, Russia (K. G. Mikhailov)

ZSI Zoological Survey of India, Kolkata, India

## Taxonomy

### Family Selenopidae

Simon, 1897

http://species-id.net/wiki/Selenopidae

Selenopinae Simon 1897: 23. Type genus *Selenops* Latreille, 1819.

#### Definition.

Benoit (1968) clearly defined the family Selenopidae. Here, we revise this definition to accommodate new species and new genera within the family. All members of the Selenopidae are extremely dorsoventrally flattened, have two tarsal claws and laterigrade legs. They are ecribellate, entelegynes, with eight eyes in two rows; with six in the first row and two in the second row (see also Jocqué and Dippenaar-Schoeman 2006).

#### Description.

Selenopidae are a variety of colors including various shades of grey, brown, yellow, and orange, with darker markings on the cephalothorax and spots or mottling on the abdomen, and annulations on the legs of most species. Chelicerae robust with 2 to 4 cheliceral teeth on each margin. Clypeus is low and chilum absent. Most genera have a longitudinal fovea with lateral radiations, 3 on each side. Labium wider than long, or as long as wide. Endites with dense terminal scopulae. Sternum oval to round with a posterior indentation; sternum extending between coxae IV. Six spinnerets; colulus absent. The legs are long and robust, with the tibiae and metatarsi of legs I and II with paired spines; these spines are the primary character that we use to separate genera. Tarsal scopulae present or absent in both males and females. Tarsal claws variable, prolateral claw is toothed and retrolateral claw is smooth in several species, but in several instances they are both toothed, with prolateral claw having more teeth than the retrolateral claw, or both claws can be smooth. Like in most spider groups, species of selenopids are differentiated by the copulatory organs, thus, the copulatory organs are variable. In many species the epigynum has a median septum and lateral lobes, however there are exceptions. Spermathecae highly sclerotized and occur in various shapes and sizes, from simple to complex (Figs 2–3). Male palps with RTA that is 2–3 branched in many species, with dorsal and ventral branches, or dorsal, median, and ventral branches (Figs 5–6, 83–84); conductor present, often sclerotized (Figs 5–6, 83–84).

#### Distribution.

The Selenopidae occur worldwide and are primarily tropical and subtropical, though several species are found in deserts, and can be found from sea level to over 2500 meters.

##### Key to genera of Selenopidae

Females (those of *Godumops* gen. n.are unknown)

**Table:** 

1	Two pairs of ventral spines on each Ti I and II, and on Mt I and II (known from Madagascar and Reunion Island)	*Hovops*
–	Spination otherwise	2
2(1)	Three pairs of ventral spines on Ti I and II, and two pairs of spines on Mt I and II	3
–	Spination otherwise	4
3(2)	With tarsal scopulae (found in Africa, Asia, southern Europe and the New World)	*Selenops*
–	Without tarsal scopulae (found in India and Nepal)	*Makdiops* gen. n.
4(2)	Found in Africa or Madagascar	5
–	Found elsewhere	6
5(4)	Spination pattern on ventral Ti and Mt I and II 4–3 and found in Madagascar	*Garcorops*
–	Spination otherwise, or if 4–3, not found in Madagascar, but Africa	*Anyphops*
6(4)	Tibia I and II with 4 ventral spines, Mt I and II with 3 ventral spines (found in India)	*Makdiops* gen. n.
–	Other spination pattern	7
7(6)	With 7 pair of ventral spines on Ti I and II, and 5 pairs on Mt I and II (found in Taiwan)	*Pakawops* gen. n.
–	With a different number of ventral spines on Ti and Mt I and II	8
8(7)	Epigynum not divided into lateral lobes, with very small, simple spermathecae (Figs 1–2) (found in the Philippines)	*Amamanganops* gen. n.
–	Epigynum either divided into lateral lobes and/or with complex, coiled spermathecae	9
9(8)	Epigynum with posterodorsal fold covering part of the extremely coiled spermathecae (Fig. 4) (found in Southeast Asia)	*Siamspinops*
–	Epigynum without posterodorsal fold covering spermathecae (Fig. 10), or if one is present, spermathecal ducts are not coiled (Fig. 78) (found in Australia)	*Karaops* gen. n.

Males (those of *Amamanganops* gen. n.and *Pakawops* gen. n. are unknown)

**Table:** 

1	Two pairs of ventral spines on Ti I and II, and on Mt I and II (found in Madagascar and Reunion Island)	*Hovops*
–	Spination otherwise	2
2(1)	Palpus without median apophysis (Fig. 5); terminal margin of labium m-shaped (Fig. 109) (found in New Guinea)	*Godumops* gen. n.
–	Palpus with median apophysis (Figs 23, 83; Benoit 1968, Fig. 6; Corronca 2003, Figs 2C–D; Corronca 2005, Figs 1D–E) terminal margin of labium rounded	3
3(2)	Spination pattern on ventral Ti and Mt I and II 3–2	4
–	Spination pattern on ventral Ti and Mt I and II otherwise	5
4(3)	Tarsal scopulae absent (found in India and Nepal)	*Makdiops* gen. n.
–	Tarsal scopulae present (found in Africa, Asia, southern Europe and the New World)	*Selenops*
5(3)	Conductor T-shaped with basally rounded projection (Corronca 2003, Figs 2C–D) (found in Madagascar)	*Garcorops*
–	Conductor otherwise (Fig. 23; Corronca 2005, Figs 1D–E; Dankittipakul and Corronca 2009, Fig. 22)	6
6(5)	Chelicerae project forward with long fangs (found in Southeast Asia)	*Siamspinops*
–	Chelicerae and fangs otherwise	7
7(6)	Median apophysis large, complex, and strongly sclerotized, often twisted (Benoit 1968, Fig. 6; Corronca 2005, Figs 1D–E) (found in Africa and Madagascar)	*Anyphops*
–	Median apophysis much smaller, simple and tapered, either with one or two branches, lightly sclerotized and never twisted (Figs 7, 49) (found in Australia)	*Karaops* gen. n.

#### Genus Selenops

Latreille, 1819

http://species-id.net/wiki/Selenopidae

http://species-id.net/wiki/Selenops

Figs 112–115

Selenops Latreille 1819: 579. Type species *Selenops radiatus* Latreille, 1819, by original designation.
Orops Benoit 1968: 116. Type species *Selenops littoricola* Strand, 1913, by original designation. Synonymized by Corronca (1996: 60).

##### Diagnosis.

All members of this genus can be distinguished from other genera by the ventral spination on the tibiae and metatarsi of legs I and II, where there are 3 pairs of spines on the tibiae, and 2 pairs of spines on the metatarsi.

##### Remarks.

Despite the amount of recent work that has been done on the family, there is still some difficulty in determining boundaries of genera, in particular with the genus *Selenops*. (Corronca (1998, 2002) has provided diagnoses of the genera of Selenopidae, but there are many variations, and several species considered in the present study do not quite fit into the genus *Selenops* as currently defined. Walckenaer (1837) first recognized three groups based on characteristics of the chelicerae, labium and leg lengths. These characters were not substantiated by Simon (1880) who attempted to divide the family into Old and New World species based on eye size. In the New World, F. O. Pickard-Cambridge (1900) first distinguished species using eye size and position along with genitalic characters. (Petrunkevitch (1925, 1930) divided these species into groups and described new species based on leg proportions. In 1953, Muma established six species groups for species from North and Central America and the Caribbean based on leg lengths, eye size and position, and genitalic characters. Current authors (Alayón-García 1992, 2001, 2003, 2005; Valdez-Mondragon 2007, 2010) still use these characters and groupings despite variation and species that do not fit into any group (Crews in press).

Additionally, molecular phylogenetic work (Crews and Gillespie 2010) and morphological data indicate that the monophyly of the genus *Selenops* is somewhat questionable. In the molecular phylogenies, *Selenops* is either para- or polyphyletic, though these results lack significant support in all trees. The para- and polyphyly occurs between the Old and New World selenopids. That is, the New World selenopids are monophyletic, and the Old World selenopids are not. Benoit (1968, p. 118) noted that American *Selenops* are very different from the African ones, and that they have nothing at the generic level in common with species from the Old World. He suggested that they should be the object of a new classification. Unfortunately, he did not elaborate further. We examined multiple species of New and Old World *Selenops*, but we have no better conclusion than Benoit (1968). We are unable to find any morphological characters that consistently differentiate Old and New World *Selenops*, and do not have enough molecular data from Old World species, and thus the genus is retained for both groups at this time.

##### Description.

Total length 4–20. *Cephalothorax:* Carapace with some marks, wider than long; long, narrow fovea with 6 radiating lines. Setae either plumose or stiff, sometimes both types occur on same specimen; clypeus low. Eyes: 6 eyes in anterior row, either in a straight line or slightly recurved; PME larger than AME in most specimens, but in some specimens equal or, rarely, smaller; eye size occasionally differs between sexes of same species; Chelicerae slightly geniculate, robust, with 3 prolateral and 2 retrolateral teeth. *Legs:* Leg II longer than leg IV in most species, however, this is not always the case. Leg lengths are highly variable in this genus, and do not seem to be a good indicator of phylogeny or classification; tibia and metatarsai with 3 and 2 ventral spines, respectively. Tarsal, and in some species, metatarsal scopulae present, especially in females. *Female copulatory organs:* Epigynum usually with lateral lobes, occasionally with epigynal pockets; some species have a posterodorsal fold (Crews, in press), which is an extension of the external copulatory organs that folds in and may cover spermathecae or internal ducts. *Male copulatory organs:* Tibia typically with 2, and in some species 3 apophyses, with dorsal apophysis longer than ventral one in most species; median apophysis is one or two branched, and can be translucent, or with one or both branches sclerotized.

##### Distribution.

*Selenops* occurs in the New World from southern North America, throughout Central America, to southern South America, including islands in the Caribbean Sea. In the Old World, *Selenops* occurs throughout Africa, the Mediterranean, Middle East, and Asia including Japan and Taiwan.

##### Composition.

Currently there are 124 species of *Selenops* described. Revisionary work of the African *Selenops* species was done first by Lawrence (1940), followed by Benoit (1968), and Corronca (2002). Corronca (2001) has also described new species from the African region. *Selenops radiatus*, the type of the genus, is the most widespread species, occurring from northern Africa, throughout the Middle East and Mediterranean and into India and China. In the New World, Muma (1953) revised the North American, Central American and Caribbean Selenopidae. Corronca (1998) revised South American representatives. Alayón-García (2005) has revised the Cuban species and reviewed and described species from other Caribbean islands such as Jamaica (Alayón-García 2003), the Dominican Republic (Alayón-García 1992), and Curaçao (Alayón-García 2001). (Valdez-Mondragón (2007, 2010) has also described a handful of species from México. The most recent revision of the North and Central American and Caribbean species was done by Crews (in press). Below we include a special section reviewing the *Selenops* species found in China, as the literature is difficult to obtain.

###### Selenops from China

The Chinese species have been reviewed by Zhu et al. (1990). Currently, there are only three species described from this vast region, one of which is widespread throughout eastern Asia, including China, Japan, Korea and Taiwan, and one has the largest range of any member of the genus. Here we review the species, synonymize one species, and provide collecting localities, information on the types and some natural history data.

##### Selenops bursarius

Karsch, 1879

http://species-id.net/wiki/Selenops_bursarius

Selenops bursarius Karsch 1879: 81, plate 1, fig. 2.
Selenops henanensis Zhu and Mao 1983: 151, figs a-e. Synonymized by Zhu et al. (1990).

###### Type material.

Male and female syntypes: Japan (ZMB 2679, 2692, 3501-52, not examined).

###### Distribution.

This species has been found in China, Japan, Korea and Taiwan. In China, the species has been found in Sichuan (Chengdu, Xiushan), Henan (Xinyang), Jiangsu (Suzhou), and Zhejiang (Zhu et al. 1990).

###### Remarks.

In molecular phylogenetic analyses (Crews and Gillespie 2010), *Selenops bursarius* does not group with other *Selenops* species, but is instead always allied with *Karaops* gen. n. from Australia, though this relationship is not well supported. *Selenops bursarius* shares the 3–2 tibial-metatarsal ventral spination with Old and New World *Selenops* species, however the male palps are unique among selenopids. The RTA is very elaborate and consists of three apophyses; large dorsal and medial apophyses and a smaller ventral apophysis. The embolus resembles that of some *Karaops* gen. n.species in its shape and origin. The MA is bulbous, two-branched and highly sclerotized, a unique feature. Finally, the conductor is somewhat T-shaped, a characteristic found in several selenopid genera. We have chosen to retain this species in *Selenops* at the present time, though clearly it retains unique features and may indeed represent an undescribed lineage.

###### Natural history.

In China, it has been found on cedar (*Cryptomeria japonica*), where it hides under the bark during the day and comes out at night (Zhu et al. 1990).

##### Selenops ollarius

Zhu, Sha et Chen, 1990

http://species-id.net/wiki/Selenops_ollarius

Map 1

Selenops ollarius Zhu et al. 1990: 32, figs 9–10.

###### Type material.

Female holotype: Leshan Buddha Temple, Sichuan Province, China [29°34'N, 103°41'E], 23.X.1975, Z. Chuandian (Norman Bethune Medial University, Department of Biology, Changchun, Jilin; not examined).

###### Distribution.

**Map 1.**
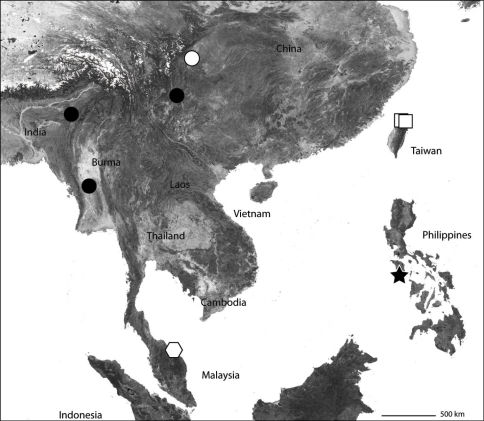
Part of Asia showing the known distribution of the Selenopidae in the region. *Selenops radiatus* Latreille (black circles), *Selenops ollarius* Zhu, Sha and Chen (white circle), *Pakawops formosanus* comb. n. (white squares), *Amamanganops baginawa* (black star), *Siamspinops aculeatus* comb. n. (white hexagon).

Only known from the type locality (Map 1).

###### Remarks.

*Selenops ollarius* clearly belongs in the genus *Selenops*. The epigynum resembles that of *Selenops radiatus* and other Old World Selenopidae.

##### Selenops radiatus

Latreille, 1819

http://species-id.net/wiki/Selenops_radiatus

Fig. 114 Map 1

Selenops cordatus Zhu et al. 1990: 31, figs 5–8. **syn. n.**

###### Type material.

*Selenops cordatus*: Holotype female: Binggu Orchards, Miyi County, Sichuan Province, China [27°07'N, 102°01'E], IX–X.1980 (Sichuan Academy of Agricultural Science Institute of Plant Protection; not examined). Paratypes: Males and females, same data as holotype (Norman Bethune Medial University, Department of Biology, Changchun, Jilin; not examined).

###### Distribution.

Known only from Sichuan Province in China (Map 1); however, it is widespread from Africa, throughout the Mediterranean, India, and other parts of Asia. Thus, it is likely found elsewhere in China.

###### Remarks.

It is clear from the drawings provided by Zhu et al. (1990) that *Selenops cordatus* is a junior synonym of *Selenops radiatus*. This extends the range of *Selenops radiatus* even further east (Map 1), making it the most widespread member of the Selenopidae, the phylogeography of which would no doubt be interesting to study.

#### Genus Amamanganops gen. n.

urn:lsid:zoobank.org:act:7BA592FD-7EA9-459F-B31E-8D98C7FEAC51

http://species-id.net/wiki/Amamanganops

##### Type species:

*Amamanganops baginawa* sp. n.

##### Etymology.

*Amamanganops* gen. n.comes from a combination of words and honors the indigenous peoples from the region of the type locality of this selenopid. Hanunuo Mangyan: *Amamangan* = spider; Greek: *ops* = face, eye. We retain the traditional ending of selenopid genera of *ops*, which originally referred to the eye arrangement. The gender is masculine.

##### Diagnosis.

**Figures 1–6.**
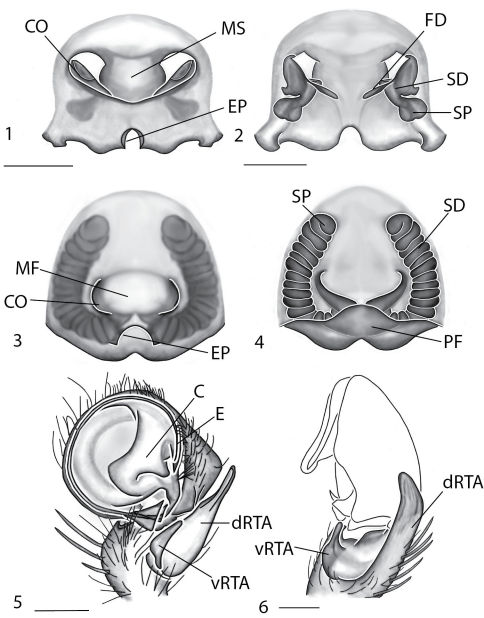
Copulatory organs of *Amamanganops baginawa* sp. n., the holotype from Mindoro Island, Philippines (CAS 9031787) (**1–2**), *Siamspinops aculeatus* (Simon, 1901) comb. n. from Gunong, Malaysia (UMZC) (**3–4**) and *Godumops caritus* sp. n., the holotype from Baiteta Forest, Papua New Guinea (RMCA) (**5–6**): **1, 3** epigyne, ventral view **2, 4** spermathecae, dorsal view **5** male pedipalp, ventral view **6** male pedipalp, retrolateral view. Scale bar: (1–2) 0.5 mm, (5–6) 0.25 mm. Abbreviations: **CO** = copulatory opening, **MS** = median septum, **EP** = epigynal pockets, **FD** = fertilization ducts, **SD** = sperm ducts, **SP** = spermathecae, **MF** = median field, **PF** = posterodorsal fold, **C** = conductor, **E** = embolus, **dRTA** = dorsal branch of retrolateral tibial apolphysis, **vRTA** = ventral branch of retrolateral tibial apophysis.

*Amamanganops* gen. n. can be separated from all other genera by genitalic characters. The epigynum is the simplest known for all Selenopidae. It is not divided into lateral lobes, has a sinuous posterior margin, and has extremely simple and small internal ducts (Figs 1A–B). Males unknown.

##### Description.

Total length 6.90. *Cephalothorax*: Carapace with some dusky markings, wider than long. Fovea short, broad, and shallow. Setae variable, simple, with both long and thin and short and thick hairs present. AER straight, PER slightly recurved. PME equal to AME. Chelicerae slightly geniculate, robust, 3 prolateral and 2 retrolateral teeth. *Legs:* Leg II longer than leg IV, leg III longest. Right leg I has 4 paired spines on tibia and 3 on metatarsus; left leg is missing. Leg II has 5 paired spines on tibia and 3 on metatarsus. Tarsal scopulae absent. *Female copulatory organs:* Epigynum without lateral lobes, with a sinuate posterior margin, and epigynal pockets. Spermathecae small and simple (Figs 1–2).

##### Distribution.

*Amamanganops* gen. n. is known from a single specimen collected around San Jose, on the southern part of the island of Mindoro (Map 1). It is likely found on other parts of the island.

##### Composition.

A single species, *Amamanganops baginawa* sp. n.

##### Amamanganops baginawa sp. n.

urn:lsid:zoobank.org:act:A15B659A-CEC4-48C8-B794-965EBE50AB55

http://species-id.net/wiki/Amamanganops_baginawa

Figs 1–2 103 Map 1

###### Type material.

Holotype female: San Jose, 12°23'N, 121°04'E, Mindoro Island, Philippines, III.1945, E.S. Ross (CAS 9031787).

###### Etymology.

The specific epithet comes from the Buhid Mangyan word *baginawa*, meaning spider in the language of the indigenous people inhabiting the region of the type locality. The name is to be treated as a noun in apposition.

###### Diagnosis.

This species can be differentiated from all others by the very simple internal copulatory organs (Fig. 2). Males unknown.

###### Description.

*Holotype:* Color: carapace yellow-brown, with slightly darker marks laterally; sternum pale yellow-brown; chelicerae yellow-brown with darker infuscations anteriorly and laterally; labium pale yellow-brown; abdomen dorsally yellowish, faded, but lateral dark areas present, a dark lanceolate stripe, w-shaped mark 3/4 way to end, and festoon present; ventrally pale yellow-brown; legs yellow brown with darker annulations, legs darkening distally, underside of femora with longitudinal dark area. Cephalothorax:setae short, stout, rodlike; 0.86 times longer than broad; fovea longitudinal, broad, somewhat shallow. Eyes:AER nearly straight; PER slightly recurved; PME same size as AME, PLE largest, ALE smallest; eye group width 1.24; eye diameters, AME 0.17, ALE 0.03, PME 0.17, PLE 0.25; interdistances AME-ALE 0.31, PME-PLE 0.31, ALE-PLE 0.17, AME-PME 0.04; ocular quadrangle AME-AME 0.10, PME-PME 0.42; clypeus 0.11 high. Mouthparts:lateral boss present, smooth; promargin with three teeth, retromargin with two teeth; maxillae longer than broad, with tuft of conspicuous setae distally; labium distally rounded. Sternum:0.95 times longer than broad, posteriorly indented. Pedipalp:tarsus slightly swollen, claw present with less than six teeth. Legs:Leg I only slightly shorter than legs II, III and IV; leg formula 3241; leg III longest; scopulae absent on all legs; tarsus I–IV with strong claw tufts on all legs; pr claw with less than 10 teeth, rl claw with none; spination: leg I, Fm pr 1–1–0, d 1–1–1, rt 0; Ti v 2–2–2–2; Mt v 2–2–2; Ti and Mt I and II with strong spines; leg II, Fm pr 0, d 1–1–1, rt 0; Ti 2–2–2–2; Mt v 2–2–2; leg III, Fm pr 0, d 1–1–1, rt 0; Ti 0; Mt 0; leg IV, Fm pr 0, d 1–1–1, rt 0; Ti 0; Mt 0. Abdomen:Terminal setal tufts present. Epigyne:Lateral lobes not distinct, median septum, copulatory openings located anterolaterally, posterior margin sinuate, epigynal pockets present; spermathecae very small and simple, well-separated, posterodorsal fold absent. Dimensions:Total length 6.93. Cephalothorax length 2.64, width 3.08. Sternum length 1.47, width 1.55. Abdomen length 4.29, width 3.11. Pedipalp: Fm 0.77, Pt 0.57, Ti 0.54, Ta 0.73, (total) 2.61. Leg I: Fm 2.64, Pt 1.34, Ti 2.10, Mt 1.53, Ta 0.63, (total) 8.24. Leg II: Fm 3.37, Pt 1.32, Ti 2.69, Mt 1.93, Ta 0.82, (total) 10.13. Leg III: Fm 3.77, Pt 1.15, Ti 2.77, Mt 2.10, Ta 0.80, (total) 10.59. Leg IV: Fm 3.40, Pt 1.03, Ti 2.16, Mt 1.61, Ta 0.75, (total) 8.95.

###### Distribution.

The type locality only (Map 1).

#### Genus Anyphops

Benoit, 1968

http://species-id.net/wiki/Anyphops

Figs 104–105

Anyphops Benoit 1968: 115. Type species: *Selenops atomarius*Simon 1887, by original designation.

##### Diagnosis.

*Anyphops* can be separated from all other genera by the ventral leg spination of Ti and Mt I and II, coupled with the collection locality. Specimens have either 4, 5, 6 or 7 paired ventral tibial spines and are found in Africa or Madagascar. If the tibial-metatarsal spination is 4–3 the spider is found in Africa and not Madagascar (see below under Remarks). In males, an additional character useful for diagnosis is the large, complex, sclerotized and often twisted MA.

##### Remarks.

Corronca (1998) described a single species of *Anyphops* from Madagascar, *Anyphops benoiti*. *Anyphops* had previously been known only from Africa while at the time only one genus, *Hovops*, had been described from Madagascar. Presumably *Anyphops benoiti* was included in the genus *Anyphops* due to the leg spination on the metatarsi and tarsi of legs I and II (5–3). In 2003, Corronca described a new genus, *Garcorops*, endemic to Madagascar. He mentioned differences between *Anyphops* and *Garcorops*, and that *Garcorops* seemed to be morphologically closest to the B1 group of *Anyphops*, as defined by Lawrence (1940) from Kenya and South Africa. *Anyphops benoiti* was not specifically mentioned in this paper. Although we have not examined specimens of *Anyphops benoiti*, Corronca’s illustration of the male palpus does not show the large, twisted median apophysis found in the majority of *Anyphops* species, but has a somewhat T-shaped conductor similar to that found in *Garcorops*, though *Anyphops benoiti* does not have the projection on the conductor that Garcorops has. The illustrations of the female copulatory organs are similar to both *Anyphops* and *Garcorops*. While it may seem that leg spination may not be a very strong character by which to separate genera, we would like to point out that in the molecular study of Crews and Gillespie (2010), while not having all genera discussed in the current paper available for genetic study, the genera *Karaops* gen. n., *Garcorops*, *Hovops*, and *Anyphops* were distinct in all analyses. After examining many other morphological characters in detail, the leg spination, coupled with biogeographical data and genetic data, appears to be the best way to distinguish genera at the present time given the specimens that are available.

##### Description.

Total length 4.00–17.40. *Cephalothorax:* Carapace with dark bands or spots laterally, wider than long or equally as long as wide, with cephalic portion more noticeable than in *Selenops*. Narrow fovea with six radiating lines. Setae are simple and sometimes spiniform. Median eyes strongly recurved, PME>AME. Chelicerae slightly geniculate, robust, with 3 prolateral and 2 retrolateral teeth. *Legs:* Leg IV longer than leg II, and leg lengths are typically 4321. Tibiae I and II with pairs of 7, 6, 5 or 4 spines. Tarsal, and in some species, metatarsal scopulae present. *Female copulatory organs:* Epigynum with or without lateral lobes, with well defined median field, depression or septum. Epigynal pockets sometimes present. Spermathecae simple or complex. *Male copulatory organs:* Palpal cymbium with dense terminal scopulae. Palpal tibia with 2 tibial apophyses, dorsal larger than ventral. Dorsal apophysis twisted in some species, and in some species both branches bifurcated. MA complex, strongly sclerotized, angular, and twisted in some species.

##### Distribution.

*Anyphops* occurs throughout Africa, as well as on the islands of St. Helena and Madagascar.

##### Composition.

Currently there are 64 species of *Anyphops* described. Most species were first described as members of the genus *Selenops* by Lawrence (1940). Benoit (1968) transferred these into the genus *Anyphops* and described additional species. (Corronca (1998, 2000, 2005) described five more species, and re-described the *lycosiformis* group.

#### Genus Garcorops

Corronca, 2003

http://species-id.net/wiki/Garcorops

Fig. 106

Garcorops Corronca 2003: 387. Type species: *Garcorops madagascar* Corronca, 2003, by original designation.

##### Diagnosis.

*Garcorops* is easily separated from all other genera by the T-shaped conductor with a basally rounded projection. Females are best recognised from most other genera by the presence of 4 ventral pairs of spines on the tibiae and 3 pairs on the metatarsi, although there are some species of *Anyphops* with similar spination.

##### Description.

Total length 5.30–6.90. *Cephalothorax:* Carapace with some light markings, wider than long. Long narrow fovea with 6 radiating lines. Setae simple. Clypeus low. Eye rows recurved, with PME larger than AME. Chelicerae slightly geniculate, robust, with 3 prolateral and 2 retrolateral teeth. *Legs:* Leg IV longer than leg II. Tibiae and metatarsi with 4 and 3 pairs of ventral spines, respectively. *Female copulatory organs:* Epigynum with distinct lateral lobes in most species connected by a sclerotized bridge. Median field depressed, epigynal pockets absent, spermathecae complex. *Male copulatory organs:* Palpal tibia with 2 tibial apophyses, dorsal larger than ventral. MA unbranched and not sclerotized, conductor T-shaped with basally rounded projection.

##### Distribution.

*Garcorops* is found in Madagascar and the Comoros Islands.

##### Composition.

Currently there are three described extant species of *Garcorops*, *Garcorops jocquei* Corronca, 2003, *Garcorops madagascar* Corronca, 2003 and *Garcorops paulyi* Corronca, 2003, all recently described by Corronca (2003). A fourth species, *Garcorops jadis*, has been found in Madagascan copal, and was described by Bosselaers (2004). It has been suggested by Penney et al. (2005) that this could represent an ‘undiscovered’ extant species, or an extinct species.

#### Genus Godumops gen. n.

urn:lsid:zoobank.org:act:271213BF-F851-4FC2-88FE-1583D0B911CE

http://species-id.net/wiki/Godumops

##### Type species:

*Godumops caritus* sp. n.

##### Etymology.

*Godumops* **gen. n** comes from a combination of words and honors the indigenous peoples of Papua New Guinea. Although there are many different indigenous groups and languages in Papua New Guinea, we chose the Nobonob language, as this language is spoken around the type locality. Nobonob: *Godum =* spider; Greek: *ops* = face, eye. We retain the traditional ending of selenopid genera of *ops*, which originally referred to the eye arrangement. The gender is masculine.

##### Diagnosis.

*Godumops* gen. n.can be separated from all other genera by a combination of characters. In males, MA is lacking (Figs 5–6) with no distinct fovea and no radiating lines on the cephalothorax (Fig. 107). The terminal portion of the labium is also m-shaped (Fig. 109), whereas it is rounded in all other genera. Females unknown.

##### Description.

Total length 4.50. *Cephalothorax:* Carapace slightly darker on edges, longer than wide, fovea indistinct, round, extremely shallow, lacking radiating lines. Setae variable, ranging from soft, to thick and coarse, short peg-like spines to long and thin; some are of medium length and thickness. Both AER and PER slightly recurved. PME smaller than AME. Chelicerae slightly geniculate, robust, with three prolateral and 2 retrolateral teeth. *Legs:* Leg II is the longest, followed by III, IV and I. Tibial and metatarsal ventral spination is 7–4. Tarsal scopulae absent. *Male copulatory organs:* Palpal tibia with 2 tibial apophyses. Dorsal apophysis much longer than ventral apophysis. MA absent.

##### Distribution.

**Map 2.**
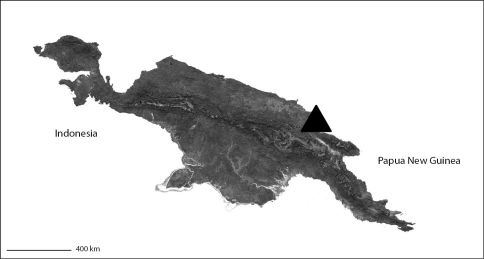
Papua New Guinea showing the distribution of *Godumops caritus* sp. n. (black triangle).

Known only from the type locality (Map 2). It is very likely there are many more species in the region.

##### Composition.

The genus contains a single species, *Godumops caritus* sp. n., known from a single male.

##### Godumops caritus sp. n.

urn:lsid:zoobank.org:act:20FD6560-84A9-456B-A6A9-17211C14EDFC

http://species-id.net/wiki/Godumops_caritus

Figs 5–6 107, 109 Map 2

###### Type material.

Holotype male: canopy fogging the tree *Pometia pinnata* in Baiteta Forest, 5°01'S, 145°45'E, Madang Province, Papua New Guinea, 31.III.1993, O. Missa (RMCA).

###### Etymology.

The specific epithet comes from the Latin word *caritus*, meaning lacking, devoid of, or poor, and refers to the near lack of a fovea, and lack of prosomal radiating lines, and the absence of a MA, which is found in all other known Selenopidae. The name is to be treated as an adjective.

###### Diagnosis.

This species can be separated from all other Selenopidae by a lack of MA (Fig. 1). Females unknown.

###### Description.

*Holotype:* Color: carapace uniformly yellow-brown; sternum pale yellow; chelicerae yellow-brown, slightly darker brown anteriorly, near lateral condyle; maxillae pale yellow; labium pale yellow-brown; abdomen dorsally yellow brown with darker markings laterally, a few medially, and some lighter spots medially; ventrally pale yellow-brown; legs yellowish, annulations indistinct, only distinct markings dark markings ventrally on femur I. Cephalothorax:carapace 0.96 times longer than broad; fovea small round depression, very shallow. Eyes:AER slightly recurved; PER recurved; AME slightly larger than PME, PLE=AME, ALE smallest; eye group width 1.09; eye diameters, AME 0.19, ALE 0.06, PME 0.15, PLE 0.19; interdistances AME-ALE 0.27, PME-PLE 0.23, ALE-PLE 0.19, AME-PME 0.04; ocular quadrangle AME-AME 0.11, PME-PME 0.5; clypeus 0.06 high. Mouthparts:chelicerae with a few stout setae medially and anteriorly; lateral boss present, smooth; promargin with three teeth, retromargin with two teeth; maxillae longer than broad, with tuft of conspicuous setae distally; labium slightly anchor shaped, distally m-shaped (Fig. 109). Sternum:0.97 times longer than broad, only very slightly posteriorly indented. Pedipalp:femur, spination dorsal 0–1–2; retrolateral tibial apophysis with two apophyses, dorsal apophysis at least three times longer than ventral apophysis, triangular in ventral view, broad at base, tapering distally, ventral apophysis small, tapering distally; retrolateral basal cymbial process absent; cymbial scopulae absent, cymbium round, tapering retrolaterally. Conductor pointed at tip, directed ventrally, arising on a curved stalk with a medial projection from stalk, similar to *Garcorops* species; embolus very long and slender, beginning at 5 o’clock, terminating at 3 o’clock, base of embolus with two overlapping, thin triangular structures, one with several teeth along the bottom margin; median apophysis absent. Legs:leg I only slightly shorter than legs II, III and IV; leg formula 2341; scopulae absent on all legs; tarsus I–IV with strong claw tufts; pr claw with many teeth c.10–15, rl claw lacking teeth; spination: leg I, Fm pr 1–1–1, d 1–1–1, rl 0; Ti v 2–2–2–2–2–2–2; Mt v 2–2–2–2; Ti and Mt I and II with strong spines; leg II, Fm pr 0, d 1–1–1, rt 0; tibia 2–2–2–2–2–2–2; Mt v 2–2–2–2; leg III, Fm pr 0, d 1–1–1, rt 0; Ti 0; Mt 0; leg IV, Fm pr 0, dorsal 1–1–1, rt 0; Ti 0; Mt 0. Abdomen:terminal tufts of setae may be present, difficult to tell as there is some damage to the abdomen.

*Dimensions:* Total length 4.48. Cephalothorax length 2.18, width 2.28. Sternum length 1.17, width 1.13. Abdomen length 2.30, width 1.70. Pedipalp: Fm 0.75, Pt 0.40, Ti 0.48, Ta 0.78, (total) 2.41. Leg I: Fm 2.20, Pt 0.77, Ti 1.97, Mt 1.82, Ta 0.77, (total) 7.53. Leg II: Fm 2.96, Pt 0.96, Ti 2.74, Mt 2.04, Ta 0.86, (total) 9.56. Leg III: Fm 2.93, Pt 0.88, Ti 2.36, Mt 1.91, Ta 0.77, (total) 8.85. Leg IV: Fm 2.62, Pt 0.96, Ti 2.04, Mt 1.44, Ta 0.77, (total) 7.83.

###### Natural history.

This species was collected from canopy fogging the tree *Pometia pinnata* in lowland rainforest habitat.

###### Distribution.

The type locality only (Map 2).

#### Genus Hovops

Benoit, 1968

http://species-id.net/wiki/Hovops

Fig. 108

Hovops Benoit 1968: 117. Type species: *Selenops pusillus* Simon, 1887, by original designation.

##### Diagnosis.

*Hovops* can be easily separated from all other genera by having the ventral tibial and metatarsal spination 2–2, as well as iridescent scales on the cephalothorax and abdomen.

##### Description.

*Cephalothorax:* Carapace with dark marks contrasting with white setae, longer than wide in most specimens. Fovea short and broad. Setae variable, iridescent scales present on cephalothorax and abdomen, some setae long and thin, some short and stiff. AER straight, PER slightly recurved. AME notably larger than PME, double in certain species. Chelicerae slightly geniculate, robust, with 3 prolateral and 2 retrolateral teeth. *Legs:* Tibial and metatarsal ventral spination is 2–2. Tarsal scopulae absent. *Female copulatory organs:* Epigynum variable. *Male copulatory organs:* Palpal tibia with 2 tibial apophyses, larger one curved, smaller one bent or dilated at base.

##### Distribution.

Known only from Madagascar and the island of Réunion.

##### Composition.

There are currently six described species of *Hovops*: *Hovops dufouri* (Vinson, 1863), *Hovops legrasi* (Simon, 1887), *Hovops madagascariensis* (Vinson, 1863), *Hovops mariensis* (Strand, 1908), *Hovops modestus* (Lenz, 1886) and *Hovops pusillus* (Simon, 1887). This genus is in need of revision, as the newest species description is over 100 years old, with the other descriptions being 120–150 years old. The majority of species are described in separate publications and there are only two diagnostic drawings.

#### Genus Karaops gen. n.

urn:lsid:zoobank.org:act:AE105D7B-507E-4D06-8311-95B231539E46

http://species-id.net/wiki/Karaops

##### Type species:

*Karaops ellenae* sp. n.

##### Etymology.

*Karaops* gen. n. comes from a combination of words and honors the indigenous peoples of Australia by referring to the indigenous Selenopidae found throughout the continent. Nyoongar: *Kara =* spider; Greek: *ops* = face, eye. We retain the traditional ending of selenopid genera of *ops*, which originally referred to the eye arrangement. The gender is masculine.

##### Diagnosis.

*Karaops* gen. n.can be separated from all other genera by a combination of characters: 1. ventral tibial and metatarsal spination of legs I and II something other than 3–2, 2. absence of scopulae, 3. found only in Australia. The males have a small, simple MA that is not twisted in any species.

##### Description.

Total length 3.90–10.30. *Cephalothorax:* Carapace with some dusky marks, usually wider than long. Fovea longitudinal, broad, and shallow. Setae variable, ranging from soft to thick and coarse, short peg-like spines to long and thin; some are of medium length and thickness. Chelicerae slightly geniculate, robust, with 3 prolateral and 2 retrolateral teeth, or 4 prolateral and 3 retrolateral teeth. *Eyes:* AER straight to slightly recurved to recurved, PER slightly recurved to strongly recurved. PME larger than AME in most species, though equal or smaller in some specimens. *Legs:* Leg III usually longest, though leg II or IV is longest in some species. Leg pattern 3241in most specimens, but is variable both between and within species, as in *Selenops*. Tibial and metatarsal ventral spination is primarily in pairs of 5 and 3, respectively, but can also be 6–3, 5–4, 6–4, 5–0, or are unpaired. Tarsal scopulae absent. *Female copulatory organs:* Epigynum with lateral lobes, a well-defined median area, and with or without epigynal pockets. Spermathecae and internal ducts range from simple and round to highly coiled. *Male copulatory organs:* Palpal tibia with 2 or 3 tibial apophyses. Dorsal apophysis longer than or equal to ventral apophysis in most species. MA 1 or 2 branched, ranging from unsclerotized to strongly sclerotized.

##### Remarks.

Species of*Karaops* gen. n.can be locally abundant, but are relatively rare or at least elusive given the low numbers of species in museum collections.Although none have yet been determined to be short range endemics (SREs) (Harvey 2002), it is possible that with more thorough collecting in particular areas or with additional morphometric or molecular data, some will be found to be SREs. Many species are known only from one sex, and several species are known from only a single specimen.

##### Distribution.

*Karaops* gen. n.occurs throughout mainland Australia, but is apparently absent from Tasmania (Maps 4–10).

**Map 3.**
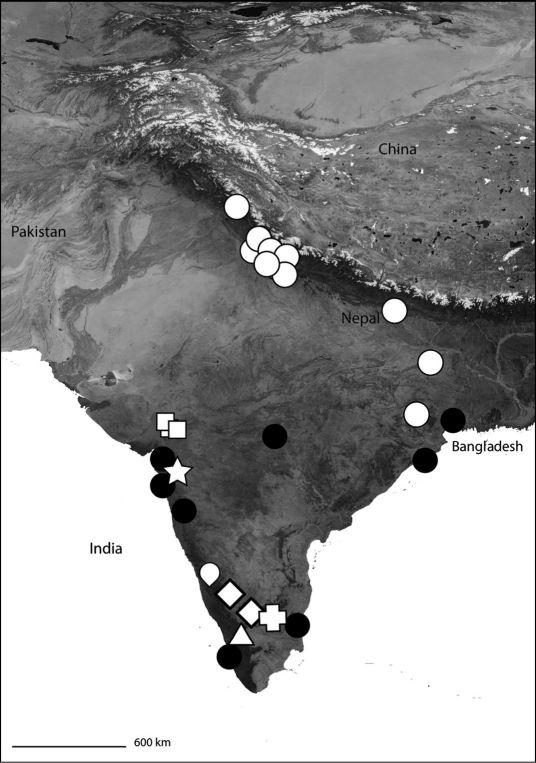
India showing the distribution of Selenopidae in the region. *Selenops radiatus* Latreille (black circles), *Makdiops nilgirensis* comb. n. (white triangle), *Selenops shevoyarensis* Gravely (white plus sign), *Makdiops mahishasura* sp. n. (white diamonds), *Makdiops agumbensis* comb. n. (white tear drop), *Makdiops shiva* sp. n. (white star), *Selenops sumitrae* Patel & Patel (white squares), *Makdiops montigenus* comb. n. (white circles).

##### Composition.

In addition to transferring *Selenops australiensis* to *Karaops* gen. n., we describe 23 new species. It is likely that more species will be found. Given the large ranges of some species, yet seemingly stable morphology across isolated populations, molecular or morphometric data may reveal cryptic species.

##### Key to Karaops species

(males of *Karaops monteithi* sp. n., *Karaops marrayagong* sp. n., *Karaops badgeradda* sp. n., *Karaops julianneae* sp. n., *Karaops martamarta* sp. n., *Karaops deserticola* sp. n., *Karaops jenniferae* sp. n., *Karaops dawara* sp. n. unknown; females of *Karaops alanlongbottomi* sp. n., *Karaops keithlongbottomi* sp. n., and *Karaops larryoo* sp. n. unknown)

**Table:** 

1	Males	2
–	Females	17
2(1)	Ventral spines on tibiae I and II unpaired	3
–	Ventral spines on tibiae I and II paired	4
3(2)	Base of embolus between 3 and 5 o’clock, base of median apophysis subquadrangular, with single branch curving slightly distally in ventral view (Fig. 29)	*Karaops raveni* sp. n.
–	Base of embolus between 4 and 6 o’clock, base of median apophysis ovoid, with single branch pointed retrolaterally in ventral view (Fig. 23)	*Karaops jarrit* sp. n.
4(2)	Cheliceral promargin with 4 teeth	5
–	Cheliceral promargin with 3 teeth	6
5(4)	Cymbium pointed at tip, conductor terminates at 1 o’clock, base of embolus large and rounded, extending almost to base of cymbium (Fig. 71)	*Karaops toolbrunup* sp. n.
–	Cymbium rounded at tip, conductor terminates at nearly 3 o’clock, base of embolus angular (Fig. 65)	*Karaops francesae* sp. n.
6(4)	Tibial apophyses with 3 processes	7
–	Tibial apophyses with 2 processes	9
7(6)	Conductor crescent-shaped, narrowing very abruptly in the middle, forming a long, narrow, scythe-shaped terminus (Fig. 17)	*Karaops alanlongbottomi* sp. n.
–	Conductor shaped otherwise or not narrowing as abruptly (Figs 19, 21)	8
8(7)	Conductor angular, terminus directed proximally, strongly sclerotized, MA directed retrolaterally (Fig. 21)	*Karaops larryoo* sp. n.
–	Conductor with squarish projection medially, terminus directed retrolaterally to proximally, MA directed distally (Fig. 19)	*Karaops keithlongbottomi* sp. n.
9(6)	MA very small, and attached to base of embolus (Figs 7, 13)	10
–	MA larger, and attached elsewhere	11
10(9)	Pointed angular projection directed proximally coming off base of embolus at 6 o’clock position (Fig. 13)	*Karaops gangarie* sp. n.
–	No angular projection on base of embolus (Fig. 7)	*Karaops australiensis*
11(9)	MA with two branches (Figs 49, 51, 61)	12
–	MA with one branch (Fig. 23)	14
12(11)	Base of embolus very large, covering part of MA, quadrangular projection at the tip of the conductor (Fig. 49)	*Karaops manaayn* sp. n.
–	Base of embolus not covering part of MA, conductor pointed at tip	13
13(12)	Tip of conductor slightly undulate (Fig. 61)	*Karaops ngarutjaranya* sp. n.
–	Tip of conductor curved regularly, directed retrolaterally, portion of conductor behind MA (Fig. 51)	*Karaops vadlaadambara* sp. n.
14(12)	Dorsal tibial apophysis tapered, slender, and pointed, MA of irregular shape, conductor with pointed terminal projection (Fig. 73)	*Karaops ellenae* sp. n.
–	Dorsal tibial apophysis quadrangular, truncate in lateral view, MA with a distinct hook	15
15(14)	Embolus thick, directed distally, bisecting the cymbium, not curving or hook shaped (Fig. 55)	*Karaops pilkingtoni* sp. n.
–	Embolus with a thick base, but becoming very long and slender, curving around the edge of the cymbium	16
16(15)	MA large, directed laterally, with a small hook distally, tapering toward hook, widening to a flat, truncate tip. Space between conductor and MA. Embolus curving, but not curving around the edge of the cymbium (Fig. 35)	*Karaops burbidgei* sp. n.
–	MA directed distally, with a distal hook, rounded at the tip. Conductor with long distal processes leaving no space between MA and conductor. Embolus curving around edge of cymbium (Fig. 39)	*Karaops karrawarla* sp. n.
17(1)	Tibiae I and II each with 6 pairs of ventral spines	18
–	Tibiae I and II each with 5 pairs of ventral spines	21
18(17)	Sperm ducts highly coiled, with 4–5 coils, no obvious oval or round large spermathecae (Fig. 12)	*Karaops gangarie* sp. n.
–	Sperm ducts with less than 4 coils, large oval to round spermathecae	19
19(18)	Lateral lobes form diamond shape around median septum, epigynal pockets absent (Fig. 27)	*Karaops marrayagong* sp. n.
–	Lateral lobes not forming diamond shape around median septum, epigynal pockets present	20
20(19)	Internal ducts coiled (Fig. 68)	*Karaops francesae* sp. n.
–	Internal ducts not coiled (Fig. 70)	*Karaops toolbrunup* sp. n.
21(17)	Cheliceral promargin with 4 teeth	*Karaops dawara* sp. n.
–	Cheliceral promargin with 3 teeth	22
22(21)	Spermathecae not large and round, but small and ovoid to elongated (Figs 10, 76)	23
–	Spermathecae large and round (Fig. 78)	27
23(22)	Epigynal pockets present (Fig. 75)	*Karaops ellenae* sp. n.
–	Epigynal pockets absent	
2424(23)	Lacking a clearly defined median field, lateral lobes indistinct (Fig. 9)	*Karaops australiensis*
–	Median field and lateral lobes more distinct	25
25(24)	Median field large and keyhole-shaped (Fig. 43)	*Karaops julianneae* sp. n.
–	Median field otherwise	26
26(25)	Internal ducts long and gently curving, with less than 5 coils (Fig. 34)	*Karaops badgeradda* sp. n.
–	Internal ducts tightly coiled with more than 5 coils (Fig. 16)	*Karaops monteithi* sp. n.
27(22)	Internal ducts not coiled (Fig. 78)	*Karaops jenniferae* sp. n.
–	Internal ducts coiled at least once	28
28(27)	Lateral lobes forming diamond shape around median septum (Fig. 25)	*Karaops jarrit* sp. n.
–	Lateral lobes and median septum shaped otherwise	29
29(28)	Lateral lobes widely separated (Figs 47, 53, 57, 59)	30
–	Lateral lobes close together or fused (Fig. 63)	33
30(29)	Median septum quadrangular, no sclerotization at copulatory openings (Fig. 47)	*Karaops manaayn* sp. n.
–	Median septum shaped otherwise, copulatory openings sclerotized	31
31(30)	Spermathecae huge, nearly touching near the midline, median septum with some wrinkling (Figs 57–58)	*Karaops pilkingtoni* sp. n.
–	Spermathecae well-separated, median septum smooth	32
32(31)	Sides of median septum parallel, median septum quadrangular (Fig. 53)	*Karaops vadlaadambara* sp. n.
–	Sides of median septum coming together near the epigastric furrow, median septum subtriangular (Fig. 59)	*Karaops deserticola* sp. n.
33(29)	Median septum and lateral lobes forming a keyhole shape (Fig. 63)	*Karaops ngarutjaranya* sp. n.
–	Median septum and lateral lobes otherwise	34
34(33)	Median septum and lateral lobes fused (Fig. 45)	*Karaops martamarta* sp. n.
–	Boundaries of median septum and lateral lobes distinct	35
35(34)	Copulatory openings without proximal bilobal sclerotization (Fig. 31)	*Karaops raveni* sp. n.
–	Copulatory openings with proximal bilobal sclerotization (Figs 37, 41)	36
36(35)	Copulatory openings located medially (Fig. 37)	*Karaops burbidgei* sp. n.
–	Copulatory openings located in the upper 1/3 of the epigynal plate (Fig. 41)	*Karaops karrawarla* sp. n.
–	Copulatory openings located in the upper 1/3 of the epigynal plate (Fig. 41)	*Karaops karrawarla* sp. n.

The following synopsis of *Karaops* species is based on similarities of the copulatory organs between species.

##### Karaops australiensis

(L. Koch, 1875) comb. n.

http://species-id.net/wiki/Karaops_australiensis

Figs 7–10 Map 4

Selenops australiensis Koch 1875: 615, plate 43, fig. 6. Koch 1876: 832, plate 71, fig. 3.

###### Type material.

Holotype immature (ZMH, not examined): Bowen [20°01'S, 148°15'E], Queensland, Australia.

###### Other material examined.

**AUSTRALIA: Queensland:** Johansen’s Cave, 23°09'S, 150°28'E, 29.V.2000, from fogging trees with pyrethrum, vine scrub, 100 m, G.B. Monteith, 1♂ (QM S57515); from base of Jim Crow Mountain [23°13'S, 150°38'E], VII.1982, A. Rozefelds, 1♀ (QM S61054); Brandy Creek [20°21'S, 148°43'E], 15.IV.1975, R. Monroe, J. Covacevich, P. Filewood, 1♂ (QM S47115).

###### Diagnosis.

**Figures 7–14.**
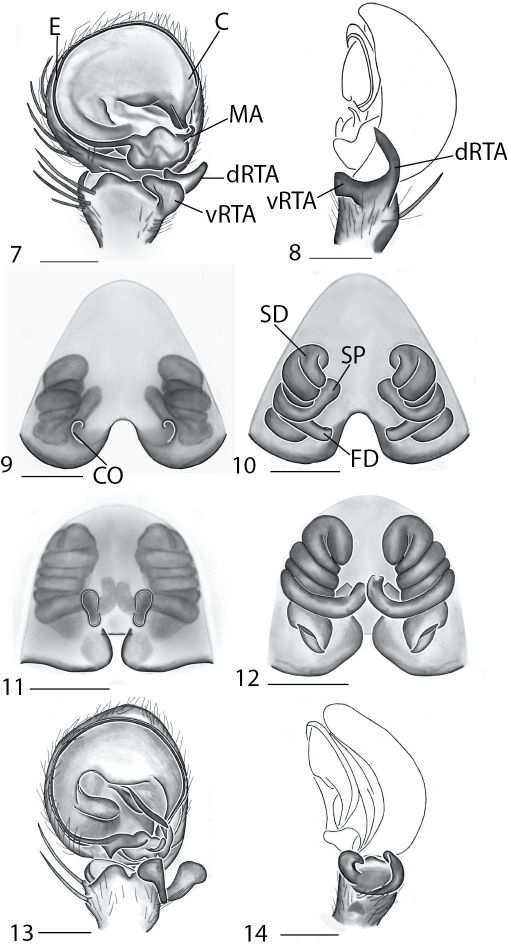
Copulatory organs of *Karaops australiensis* comb. n., male from Johansens’s Cave, Queensland, Australia (QM S57515) (**7–8**) and female from Jim Crow Mountain, Queensland, Australia (QM S61054) (**9–10**), and *Karaops gangarie* sp. n., female holotype from Amos Bay, Northeast Queensland, Queensland, Australia (QM S52315) (**11–12**) and male from Amos Bay, Northeast Queensland, Queensland, Australia (QM S88644): **7, 13** male pedipalp, ventral view **8, 14** male pedipalp, retrolateral view **9, 11** epigyne, ventral view **10, 12** spermathecae, dorsal view. Scale bar: 0.25 mm. Abbreviations: **CO** = copulatory opening, **FD** = fertilization ducts, **SD** = sperm ducts, **SP** = spermathecae, **C** = conductor, **E** = embolus, **dRTA** = dorsal branch of retrolateral tibial apolphysis, **vRTA** = ventral branch of retrolateral tibial apophysis.

The male coiled, the small MA that is attached to the base of the cymbium (Fig. 7). Females can be separated from other species by the coiled sperm ducts that lead to small, ovoid spermathecae, and epigynal pockets are absent (Figs 9–10).

###### Remarks.

The holotype from Bowen, north-eastern Queensland, is an immature (L. Koch 1875) and therefore unidentifiable to species level. We have assigned several adult specimens collected near Bowen to this species, in the assumption that only a single species occurs in the region. Although males and females have not been collected sympatrically, we have assigned them to the same species as they were collected less than 20 km apart.

###### Description.

*Male (QM S57515)*Color: Carapace uniformly yellow-brown; sternum pale yellow-brown; chelicerae pale yellow with darker infuscations anteriorly; maxillae pale yellow-brown; labium pale brown; abdomen dorsally dark grey, with pale patches anteriorly, dorsally and posteriorly; ventrally pale yellow-brown; legs with all segments clearly annulated. Cephalothorax:setae long and thin; 0.89 times longer than broad; fovea longitudinal, broad, very shallow. Eyes:AER nearly straight; PER slightly recurved; PME larger than AME, PLE largest, ALE smallest; eye group width 1.27; eye diameters, AME 0.16, ALE 0.08, PME 0.18, PLE 0.27; interdistances AME-ALE 0.24, PME-PLE 0.18, ALE-PLE 0.10, AME-PME 0.03; ocular quadrangle AME-AME 0.43, PME-PME 0.8; clypeus 0.10 high. Mouthparts:chelicerae with a few stout setae medially and anteriorly; lateral boss present, smooth; promargin with 3 teeth, retromargin with 2 teeth; maxillae longer than broad, with tuft of conspicuous setae distally; labium distally rounded. Sternum:0.83 times longer than broad, posteriorly indented. Pedipalp:femur, spination dorsal 0–1–1; retrolateral tibial apophysis with 2 processes, ventral apophysis short and quadrangular in lateral view, apophysis triangular, dorsal apophysis longer, curved and pointed at tip; retrolateral basal cymbial process absent; cymbial scopulae absent, cymbium round in ventral view; conductor very large, pointed at tip, terminating at 3 o’clock; base of embolus quadrangular and sinuate, abruptly constricted into very long, thin embolus that curves around edge of cymbium, beginning at 4 o’clock, terminating at 3 o’clock; MA a small short hook, attached to base of embolus. Legs:leg I only slightly shorter than legs II, III and IV; leg formula 3241; scopulae absent on all legs; tarsus I–IV with strong claw tufts on all legs; pr claw with c. 10–15 teeth, rl claw lacking teeth; spination: leg I, Fm pr 1–1–0, d 1–1–1, rl 0; Ti d 0, v 2–2–2–2–2; Mt v 2–2–2; Ti and Mt I and II with strong spines; leg II, Fm pr 0, d 1–1–1, rl 0–1–1; Tiv 2–2–2–2–2; Mt v 2–2–2; leg III, Fm pl 0, d 1–1–1, rl 0–1–1; Ti 0; Mt 0; leg IV, Fm pr 0, d 1–1–1, rl 0; Ti 0; Mt 0. Abdomen:terminal setal tufts may be likely, but hairs are worn off. *Dimensions:* Total length 4.25. Cephalothorax length 2.24, width 2.53. Sternum length 1.12, width 1.35. Abdomen length 2.16, width 2.04. Pedipalp: Fm 0.76, Pt 0.39, Ti 0.40, Ta 0.71, (total) 2.26. Leg I: Fm 2.76, Pt 1.04, Ti 2.44, Mt 2.08, Ta 1.15, (total) 9.47. Leg II: Fm 3.58, Pt 1.15, Ti 2.92, Mt 2.59, Ta 1.28, (total) 11.51. Leg III: Fm 3.93, Pt 1.09, Ti 3.08, Mt 2.67, Ta 1.21, (total) 11.98. Leg IV: Fm 3.38, Pt 0.90, Ti 2.59, Mt 2.42, Ta 1.16, (total) 10.45.

*Female (QM S61054):* Color: carapace uniformly yellow-brown; sternum pale yellow-brown; chelicerae pale yellow with darker infuscations anteriorly; maxillae pale yellow-brown; labium pale brown; abdomen dorsally dark grey, with pale patches anteriorly, dorsally and posteriorly; ventrally pale yellow-brown; legs with all segments clearly annulated. Cephalothorax:Setae long and thin; 0.83 times longer than broad; fovea longitudinal, broad, very shallow. Eyes:AER nearly straight; PER slightly recurved; PME larger than AME, PLE largest, ALE smallest; eye group width 1.46; eye diameters, AME 0.16, ALE 0.01, PME 0.20, PLE 0.27; interdistances AME-ALE 0.31, PME-PLE 0.22, ALE-PLE 0.18, AME-PME 0.05; ocular quadrangle AME-AME 0.45, PME-PME 0.89; clypeus 0.1 high. Mouthparts:chelicerae with a few stout setae medially and anteriorly; lateral boss present, smooth; promargin with 3 teeth, retromargin with 2 teeth; maxillae longer than broad, with tuft of conspicuous setae distally; labium distally rounded. Sternum:0.82 times longer than broad, posteriorly indented. Pedipalp:tarsus slightly swollen, claw present, without teeth. Legs:leg I only slightly shorter than legs II, III and IV; leg formula 3241; scopulae absent on all legs; Ta I–IV with strong claw tufts; pr claw with c. 10–15 teeth, rl claw lacking teeth; spination: leg I, Fm pr 1–1–0, d 1–1–1, rl 0; Ti d 0, v 2–2–2–2–2; Mt v 2–2–2; Ti and Mt I and II with strong spines; leg II, Fm pr 0, d 1–1–1, rl 0; Ti v 2–2–2–2–2–2; Mt v 2–2–2; leg III, Fm pr 0, d 1–1–1, rl 0; Ti 0; Mt 0; leg IV, Fm pr 0, d 1–1–1, rl 0; Ti 0; Mt 0. Abdomen:possible setal tufts, old specimen, hairs worn off. Epigyne:Lateral lobes indistinct, posterior margin with a medial arch, small comma-shaped copulatory openings on either side of arch, epigynal pockets absent; internally with 5–6 medially to laterally coiled ducts, small oblong spermathecae. *Dimensions:* Total length 6.63. Cephalothorax length 2.50, width 3.01. Sternum length 1.25, width 1.52. Abdomen length 4.35, width 3.47. Pedipalp: Fm 0.8, Pt 0.48, Ti 0.48, Ta 0.82, (total) 2.58. Leg I: Fm 2.64, Pt 1.10, Ti 2.34, Mt 1.80, Ta 0.99, (total) 8.87. Leg II: Fm 3.45, Pt 1.16, Ti 2.82, Mt 2.17, Ta 1.08, (total) 10.68. Leg III: Fm 3.80, Pt 1.14, Ti 2.81, Mt 2.16, Ta 1.06, (total) 10.97. Leg IV: Fm 3.28, Pt 0.89, Ti 2.44, Mt 2.00, Ta 1.07, (total) 9.68.

###### Natural history.

This species has been collected from trees fogged with pyrethrum in vine scrub, and has been seen under the bark of eucalypts (R. Atkinson, pers. comm.).

###### Distribution.

**Map 4.**
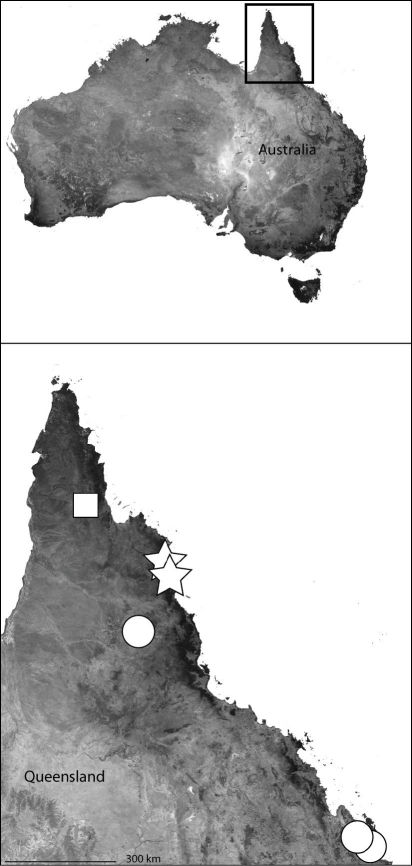
Northeast Queensland Australia (inset) showing the distribution of *Karaops*. *Karaops monteithi* sp. n. (white square), *Karaops gangarie* sp. n. (white stars), *Karaops australiensis* (L. Koch) (white circles).

This specieshas been collected from Northeast Queensland to the southern Cape York Peninsula (Map 4).

##### Karaops gangarie sp. n.

urn:lsid:zoobank.org:act:921C1C8C-B042-4D8A-AB40-38E9A63FB319

http://species-id.net/wiki/Karaops_gangarie

Figs 11–14 Map 4

###### Type material.

Holotype female (QM S52315): under bark in rainforest near Amos Bay [15°41'S, 145°20'E], Queensland, Australia, 15.V.1973, V.E. Davies. Paratypes: same data as holotype, 1♂ (QM S88644); 1♀, Cooktown, 15°27'49"S, 145°15'28"E, 3.I.2009, R. Raven, under bark, rainforest (QM S88003).

###### Other material examined.

**AUSTRALIA: Queensland:** same data as holotype, 1 immature ♂ (QM).

###### Etymology.

The specific epithet comes from the indigenous word for Cooktown, the type locality, in the Guugu Yimithirr language.

###### Diagnosis.

Males can be distinguished from other species by a triangular projection directed basally coming off of the base of the embolus (Fig. 13) and females can be differentiated from others by the strongly coiled ducts and no distinctly swollen spermathecae (Fig. 12).

###### Description.

*Female (holotype):* Color: carapace yellow-brown, with slightly darker marks laterally; sternum pale yellow; chelicerae pale yellow with darker infuscations anteriorly; maxillae pale yellow-brown, lightening distally; labium pale yellow-brown, lightening distally; abdomen dorsally yellow-brown with darker markings; ventrally pale yellow-brown; legs with femora, patellae and tibiae I–IV clearly annulated, yellow-brown, darkening distally; annulations not entirely encircling legs. Cephalothorax: setae long and thin to medium thickness; 0.84 times longer than broad; fovea longitudinal, broad, very shallow. Eyes:AER slightly recurved; PER recurved; PME larger than AME, PLE largest, ALE smallest; eye group width 1.21; eye diameters, AME 0.15, ALE 0.06, PME 0.21, PLE 0.27; interdistances AME-ALE 0.34, PME-PLE 0.27, ALE-PLE 0.29, AME-PME 0.06; ocular quadrangle AME-AME 0.15, PME-PME 0.52; clypeus 0.06 high. Mouthparts:chelicerae with a few stout setae medially and anteriorly; lateral boss present, smooth; promargin with 3 teeth, retromargin with 2 teeth; maxillae longer than broad, with tuft of conspicuous setae distally; labium distally rounded. Sternum:0.89 times longer than broad, posteriorly indented. Pedipalp:tarsus slightly swollen, claw present with more than 6 teeth. Legs:leg I much shorter than legs II, III and IV; leg formula 3241; leg III longest; scopulae absent on all legs; tarsus I–IV with strong claw tufts; pr claw with c. 10–15 teeth, rl claw lacking teeth; spination: leg I, Fm pr 1–1–0, d 1–1–1, rl 0; Ti d 0, v 2–2–2–2–2–2; Mt v 2–2–2; Ti and Mt I and II with strong spines; leg II, Fm pr 0, d 1–1–1, rl 0; Ti v 2–2–2–2–2; Mt v 2–2–2; leg III, Fm pr 0, d 1–1–1, rl 0; Ti v 1–1–0; Mt 0; leg IV, Fm pr 0, dorsal 1–1–1, rl 0; Ti v 1–1; Mt 1–0. Abdomen:Tufts of setae on posterior end of abdomen present. Epigyne:Lateral lobes distinct posteriorly, copulatory openings nearly midway up epigynal plate, copulatory openings comma to peanut-shaped indentations, epigynal pockets absent, posterodorsal fold absent, internal ducts coiled laterally to medially 5–6 times, fertilization ducts located posteriorly (Figs 11–12). *Dimensions:* Total length 5.61. Cephalothorax length 2.50, width 2.99. Sternum length 1.40, width 1.57. Abdomen length 3.11, width 2.77. Pedipalp: Fm 0.77, Pt 0.57, Ti 0.46, Ta 0.80, (total) 2.60. Leg I: Fm 2.77, Pt 0.96, Mt 1.89, Ta 0.99, (total) 8.92. Leg II: Fm 3.37, Pt 1.19, Ti 2.64, Mt 2.01, Ta 0.96, (total) 10.17. Leg III: Fm 3.23, Pt 1.07, Ti 2.77, Mt 2.30, Ta 1.11, (total) 10.48. Leg IV: Fm 3.23, Pt 0.96, Ti 2.45, Mt 2.10, Ta 0.86, (total) 9.60.

*Male (paratype):* Color: carapace uniformly yellow-brown; sternum pale yellow; chelicerae pale yellow with darker infuscations anteriorly and laterally; maxillae pale yellow-brown, lightening distally; labium pale yellow-brown, lightening distally; abdomen dorsally yellow-brown with darker markings; ventrally pale yellow-brown; legs with femora, patellae and tibiae I–IV clearly annulated, yellow-brown, darkening distally; annulations not entirely encircling legs. Cephalothorax:Setae long and thin to medium thickness; 0.83 times longer than broad; fovea longitudinal, broad, very shallow. Eyes:AER slightly recurved; PER recurved; PME larger than AME, PLE largest, ALE smallest; eye group width 1.15; eye diameters, AME 0.17, ALE 0.11, PME 0.23, PLE 0.27; interdistances AME-ALE 0.29, PME-PLE 0.23, ALE-PLE 0.27, AME-PME 0.04; ocular quadrangle AME-AME 0.10, PME-PME 0.46; clypeus 0.04 high. Mouthparts:chelicerae with a few stout setae medially and anteriorly; lateral boss present, smooth; promargin with 3 teeth, retromargin with 2 teeth; maxillae longer than broad, with tuft of conspicuous setae distally; labium distally rounded. Sternum:0.81 times longer than broad, posteriorly indented. Pedipalp:femur, spination dorsal 0–1–2; retrolateral tibial apophysis with 2 apophyses, nearly equal in size, in lateral view, ventral apophysis curved dorsally and dorsal apophysis curved ventrally, in ventral view, both apophyses widen distally, with a small, rounded process on each end; retrolateral basal cymbial process absent; scopulae absent. Cymbium rounded in ventral view, slightly angled basally on the retrolateral side. Conductor large, pointed at tip, terminating at 4 o’clock, two flexible processes coming off of conductor; embolus arising from a wider, sinuous base, with a triangular process, base narrows abruptly and embolus begins at 6 o’clock, ending at 4 o’clock, embolus very long and slender; MA very short, unsclerotized, curved, distally rounded, with single process, directed distally, arising from base of embolus (Fig. 13). Legs:leg formula unknown due to missing legs; scopulae absent on all legs; tarsi I–IV with strong claw tufts; pr claw with c. 10–15 teeth, rl claw lacking teeth; spination: leg I, Fm pr 1–1–0, d 1–1–1, rl 0; Ti d 0, v 2–2–2–2–2; Mt v 2–2–2; Ti and Mt I and II with weak spines. Abdomen:Possible setal tufts, but specimen is old and hairs are worn off. *Dimensions:* Total length 5.29. Cephalothorax length 2.40, width 2.87. Sternum length 1.73, width 1.40. Abdomen length 2.99, width 2.48. Pedipalp: Fm 0.77, Pt 0.38, Ta 0.77, (total) 2.30. Leg I: Fm 3.23, Pt 1.26, Ti 2.89, Mt 2.62, Ta 1.34, (total) 11.34. Leg II: Missing. Leg III: Missing. Leg IV: Missing.

###### Natural history.

This species has been collected under bark in rainforest, and under bark of *Melaleuca*.

###### Distribution.

Northeast Queensland (Map 4).

##### Karaops monteithi sp. n.

urn:lsid:zoobank.org:act:5D3829B6-5020-4601-8CB0-CAF52A3AEC6D

http://species-id.net/wiki/Karaops_monteithi

Figs 15–16 Map 4

###### Type material.

Holotype female (QM S61052): Upper Lankelly Creek [13°57'S, 143°12'E], Coen District, Queensland, Australia, 10–11.VI.1971, G.B. Monteith.

###### Etymology.

This species is named for the collector, G.B. Monteith, in honor of his amazing collecting prowess.

###### Diagnosis.

Females of *Karaops monteithi* sp. n. can be distinguished from other species by having highly coiled ducts, very small spermathecae located medially, and a small posterodorsal fold (Fig. 16). Males unknown.

**Figures 15–22.**
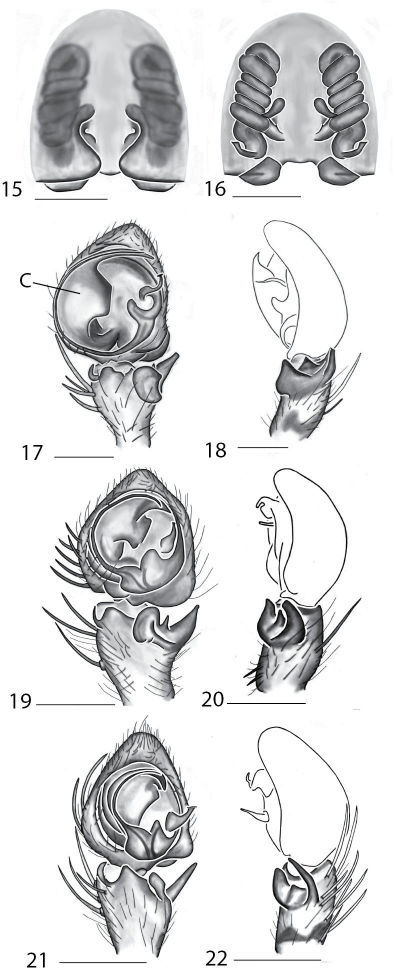
Copulatory organs of *Karaops monteithi* sp. n., female holotype from Lankelly Creek, Coen District, North Queensland, Australia (QM S61052) (**15–16**), *Karaops alanlongbottomi* sp. n., male holotype from northwest tip of Degerando Island, Champagny Islands, Western Australia, Australia (WAM T93/1330) (**17–18**), *Karaops keithlongbottomi* sp. n., male holotype from Drysdale River Station, Western Australia, Australia (WAM T55002) (**19–20)**, and *Karaops larryoo* sp. n. male holotype from north of Larryoo, Drysdale River National Park, Western Australia, Australia (WAM T93/1333) (**21–22**). **15** epigyne, ventral view **16** spermathecae, dorsal view **17, 19, 21** male pedipalp, ventral view **18, 20, 22** male pedipalp, retrolateral view. Scale bar: (15–16) 0.25 mm, (17–22)0.50 mm. Abbreviation: **C** = conductor.

###### Description.

*Holotype*: Color: carapace uniformly yellow-brown; sternum pale yellow-brown; chelicerae pale yellow with darker infuscations anteriorly and laterally; maxillae pale yellow-brown; labium pale brown; abdomen dorsally dark grey, with pale patches anteriorly, dorsally and posteriorly; ventrally pale yellow-brown; legs with femora, patellae and tibiae I–IV clearly annulated, yellow-brown, darkening distally; annulations not entirely encircling legs. Cephalothorax: Setae short, stout, rodlike, over entire habitus; 0.92 times longer than broad; fovea longitudinal, broad, very shallow. Eyes:AER nearly straight; PER recurved; PME larger than AME, PLE largest, ALE smallest; eye group width 1.68; eye diameters, AME 0.18, ALE 0.08, PME 0.22, PLE 0.3; interdistances AME-ALE 0.35, PME-PLE 0.30, ALE-PLE 0.19, AME-PME 0.04; ocular quadrangle AME-AME 0.49, PME-PME 0.99; clypeus 0.14 high. Mouthparts:chelicerae with a few stout setae medially and anteriorly; lateral boss present, smooth; promargin with 3 teeth, retromargin with 2 teeth; maxillae longer than broad, with tuft of conspicuous setae distally; labium distally rounded. Sternum:0.85 times longer than broad, posteriorly indented. Pedipalp:tarsus swollen, claw present with c. 6 teeth. Legs:leg I only slightly shorter than legs II, III and IV; leg formula 3241; scopulae absent on all legs; tarsus I–IV with strong claw tufts; pr claw with c. 10–15 teeth, rl claw lacking teeth; spination: leg I, Fm pr 1–1–0, d 1–1–1, rl 0; Ti d 0, v 2–2–2–2–2; Mt v 2–2–2; Ti and Mt I and II with strong spines; leg II, Fm pl 0, dorsal 1–1–1, rl 0; Ti v 2–2–2–2–2; Mt v 2–2–2; leg III, Fm pr 0, d 1–1–1, rl 0; Ti 0; Mt 0; leg IV, Fm pr 0, d 1–1–1, rl 0; Ti 0; Mt 0. Abdomen:possible setal tufts, old specimen, hairs worn off. Epigyne:Lateral lobes distinct posteriorly, forming a sub-diamond shaped median field, comma shaped copulatory openings laterally on median field, epigynal pockets absent internal ducts highly coiled medially to laterally 6 times, spermathecae very small and slightly oval-shaped, fertilization ducts located posteriorly, very small posterodorsal fold present on either side, does not cover any part of the internal ducts (Figs 15–16). *Dimensions:* Total length 5.97. Cephalothorax length 2.96, width 3.23. Sternum length 1.48, width 1.75. Abdomen length 3.28, width 3.02. Pedipalp: Fm 0.65, Pt 0.61, Ti 0.73, Ta 0.87, (total) 2.86. Leg I: Fm 3.11, Pt 1.37, Ti 2.81, Mt 2.16, Ta 1.16, (total) 10.61. Leg II: Fm 3.93, Pt 1.51, Ti 3.12, Mt 2.47, Ta 1.16, (total) 12.19. Leg III: Fm 4.33, Pt 1.40, Ti 3.14, Mt 2.67, Ta 1.27, (total) 12.81. Leg IV: Fm 3.85, Pt 1.19, Ti 2.93, Mt 2.57, Ta 1.27, (total) 11.81.

###### Natural history.

No data.

###### Distribution.

The type locality only (Map 4).

##### Karaops alanlongbottomi sp. n.

urn:lsid:zoobank.org:act:28DEC2CA-ED76-4867-B8A8-93A286D4260F

http://species-id.net/wiki/Karaops_alanlongbottomi

Figs 17–18 Map 5

###### Type material.

Holotype male (WAM T93/1330): northwest tip of Degerando Island, Champagny Islands, 15°20'S, 124°11'E, Western Australia, Australia, 11.VII.1988, A.F. Longbottom.

###### Etymology.

This species is named after Alan Longbottom, collector of the holotype and many other interesting arachnids for the Western Australian Museum.

###### Diagnosis.

Males can be separated from other species by having three processes of the RTA as well as a crescent-shaped conductor with a scythe shaped terminus (Fig. 17). Females unknown.

###### Description.

*Holotype:*Color: carapace uniformly yellow-brown; sternum pale yellow-brown; chelicerae pale yellow with darker infuscations anteriorly and laterally; maxillae pale yellow-brown; labium pale brown; abdomen dorsally yellow-brown with red-brown and grey markings; ventrally pale yellow-brown; legs with all segments clearly annulated. Cephalothorax:setae short, stout and rodlike, over entire habitus; 0.84 times longer than broad; fovea longitudinal, broad, very shallow. Eyes:AER slightly recurved; PER recurved; PME larger than AME, PLE largest, ALE smallest; eye group width 1.83; eye diameters, AME 0.22, ALE 0.14, PME 0.28, PLE 0.38; interdistances AME-ALE 0.37, PME-PLE 0.29, ALE-PLE 0.19, AME-PME 0.03; ocular quadrangle AME-AME 0.56, PME-PME 1.06; clypeus 0.09 high. Mouthparts:chelicerae with a few stout setae medially and anteriorly; lateral boss present, smooth; promargin with 3 teeth, retromargin with 2 teeth; maxillae longer than broad, with tuft of conspicuous setae distally; labium distally rounded. Sternum:0.91 times longer than broad, posteriorly indented. Pedipalp:femur, spination dorsal 0–1–2; retrolateral tibial apophysis with 3 processes, dorsal apophysis longest, slightly curved, tapering, medial apophysis small and triangular, ventral apophysis quadrangular in lateral view, and rounded in ventral view; retrolateral basal cymbial process absent; cymbial scopulae absent; cymbium round to triangular, angled bottom right; conductor crescent-shaped, with a squarish projection medially, pointed at tip; embolus very long and slender, beginning at 4 o’clock, terminating at 2 o’clock; MA long, slender, slightly sinuous, directed laterally then distally, with a flattened process at the tip (Figs 17–18). Legs:Leg I only slightly shorter than legs II, III and IV; leg formula 3241; scopulae absent on all legs; tarsus I–IV with strong claw tufts; claws without teeth; spination: leg I, Fm pr 1–1–0, d 1–0–1, rl 0; Ti d 0, v 2–2–2–2–2; Mt v 2–2–2; Ti and Mt I and II with strong spines; leg II, Fm pl 0–0–1, d 1–1–1, rl 0–0–1; Ti v 2–2–2–2–2; Mt v 2–2–2; leg III, Fm pl 0, d 1–1–1, rl 0–1–1; Ti v 1–1–0; Mt 0; leg IV, Fm pr 0–1–1, d 1–1–1, rl 0–1–1; Ti pr 1–0–1, v 2–2–0, rl 1–1–0; Mt pr 0, v 2–0–0, rl 1–1–0. Abdomen:without tufts of setae. *Dimensions:* Total length 6.25. Cephalothorax length 3.19, width 3.78. Sternum length 1.82, width 1.99. Abdomen length 3.15, width 2.65. Pedipalp: Fm 1.28, Pt 0.71, Ti 0.88, Ta 1.38, (total) 4.25. Leg I: Fm 4.19, Pt 1.71, Ti 3.95, Mt 3.55, Ta 1.65, (total) 15.05. Leg II: Fm 5.35, Pt 1.73, Ti 4.62, Mt 4.32, Ta 1.71, (total) 17.73. Leg III: Fm 5.74, Pt 1.77, Ti 4.91, Mt 4.34, Ta 1.98, (total) 18.74. Leg IV: Fm 5.39, Pt 1.52, Ti 4.25, Mt 4.28, Ta 1.72, (total) 17.16.

###### Natural history.

Collected from under rocks.

###### Distribution.

**Map 5.**
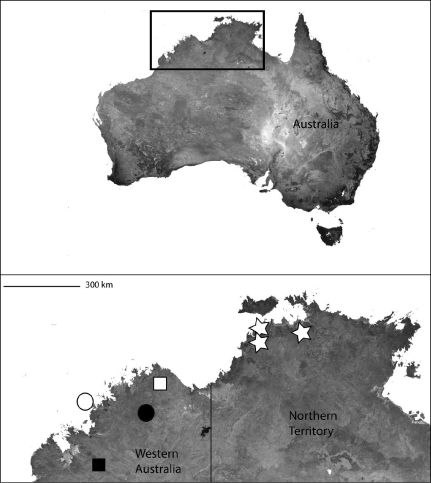
Northern Australia (inset) showing the distribution of *Karaops* gen. n. *Karaops jenniferae* sp. n. (black square), *Karaops alanlongbottomi* sp. n. (white circle), *Karaops keithlongbottomi* sp. n. (black circle), *Karaops larryoo* sp. n. (white square), *Karaops dawara* sp. n. (white stars).

The type locality only (Map 5).

##### Karaops keithlongbottomi sp. n.

urn:lsid:zoobank.org:act:6E52A0A5-4F5A-49D8-9688-F78FA28C89E2

http://species-id.net/wiki/Karaops_keithlongbottomi

Figs 19–20 Map 5

###### Type material.

Holotype male (WAM T55002): Drysdale River Station, 15°42'S, 126°23'E, Western Australia, Australia, late 1995, T. Anders.

###### Etymology.

This species is named for the late Keith Longbottom, collector of many interesting arachnids for the Western Australian Museum.

###### Diagnosis.

Males can be differentiated from other species by having an RTA with three processes, a crescent shaped conductor with a medial quadrangular lateral projection, and a MA that is directed distally (Fig. 19). Females unknown.

###### Description.

*Holotype:* Color: carapace uniformly yellow-brown; sternum pale yellow-brown; chelicerae pale yellow with darker infuscations anteriorly and laterally; maxillae pale yellow-brown; labium pale yellow-brown; abdomen dorsally yellow-brown with darker markings; ventrally pale yellow-brown; legs with femora, patellae and tibiae I–IV clearly annulated, yellow-brown, darkening distally; annulations not entirely encircling legs. Cephalothorax:Short, stout rodlike setae; 0.91 times longer than broad; fovea longitudinal, broad, very shallow. Eyes:AER nearly straight; PER recurved; PME larger than AME, PLE largest, ALE smallest; eye group width 1.86; eye diameters, AME 0.17, ALE 0.12, PME 0.31, PLE 0.44; interdistances AME-ALE 0.36, PME-PLE 0.37, ALE-PLE 0.21, AME-PME 0.04; ocular quadrangle AME-AME 0.48, PME-PME 1.04; clypeus 0.12 high. Mouthparts: chelicerae with a few stout setae medially and anteriorly; lateral boss present, smooth; promargin with 3 teeth, retromargin with 2 teeth; maxillae longer than broad, with tuft of conspicuous setae distally; labium distally rounded. Sternum:0.87 times longer than broad, posteriorly indented. Pedipalp:femur, spination dorsal 0–1–2; retrolateral tibial apophysis with 3 processes, dorsal apophysis tapering, gently curved, truncate distally, medial apophysis small, conical and pointed at tip, ventral apophysis with a round, flattened process at tip; retrolateral basal cymbial process present; scopulae absent; cymbium oval and angled bottom right; conductor crescent shaped with a quadrangular process medially, pointed at tip; embolus very long and slender, beginning at 6 o’clock, terminating at 1 o’clock; MA long, slender, tapering distally, flattened at tip, directed distally (Figs 19–20). Legs:leg I only slightly shorter than legs II, III and IV; leg formula 3241; scopulae absent on all legs; tarsus I–IV with strong claw tufts; claws without teeth; spination: leg I, Fm pr 1–1–0, d 1–1–1, rl 0; Ti d 0, v 2–2–2–2–2; Mt v 2–2–2; Ti and Mt I and II with strong spines; leg II, Fm pr 0, d 1–1–1, rl 0–1–1; Ti v 2–2–2–2–2; Mt v 2–2–2; leg III, Fm pr 0, d 1–1–1, rl 0–1–1; Ti v 1–1–0; Mt 0; leg IV, Fm pr 0, d 1–1–1, rl 0; Ti pr 1–1–0, v 2–2–0, rl 0–1–0; Mt 0. Abdomen:abdomen damaged, unknown if setal tufts are present. *Dimensions:* cephalothorax length 3.33, width 3.64. Sternum length 1.73, width 1.98. Pedipalp: Fm 1.23, Pt 0.69, Ti 0.42, Ta 1.02, (total) 3.36. Leg I: Fm 4.07, Pt 1.87, Ti 4.37, Mt 3.67, Ta 1.80, (total) 15.78. Leg II: Fm 5.22, Pt 1.98, Ti 5.11, Mt 4.26, Ta 1.94, (total) 18.51. Leg III: Fm 5.83, Pt 1.85, Ti 5.21, Mt 4.44, Ta 1.87, (total) 19.20. Leg IV: Fm 4.99, Pt 1.53, Ti 4.58, Mt 4.16, Ta 1.87, (total) 17.13.

###### Natural history.

No data.

###### Distribution.

The type locality only (Map 5).

##### Karaops larryoo sp. n.

urn:lsid:zoobank.org:act:0A05B097-3607-4C36-BC24-21753F7F3CED

http://species-id.net/wiki/Karaops_larryoo

Figs 21–22 Map 5

###### Type material.

Holotype male (WAM T93/1333): north of Larryoo, Drysdale River National Park, 14°51'S, 126°49'E, Western Australia, Australia, 12.VI.1992, M.S. Harvey, J.M. Waldock. Paratype: same data as holotype, 1♂ (WAM T93/1332).

###### Etymology.

This species is named for the type locality, and is to be treated as a noun in apposition.

###### Diagnosis.

Males can be differentiated by the presence of three processes on the palpal RTA and the tip of the conductor being heavily sclerotized and directed basally (Fig. 21). Females unknown.

###### Description.

*Holotype:*Color: carapace yellow-brown, with slightly darker marks medially; sternum pale yellow-brown; chelicerae pale yellow with darker infuscations anteriorly; maxillae pale yellow-brown; labium pale yellow-brown; abdomen dorsally yellow-brown with darker markings; ventrally pale yellow-brown; legs with all segments clearly annulated. Cephalothorax:setae short, stout, rodlike; 0.89 times longer than broad; fovea longitudinal, broad, very shallow. Eyes:AER slightly recurved; PER recurved; PME larger than AME, PLE largest, ALE smallest; eye group width 1.52; eye diameters, AME 0.17, ALE 0.11, PME 0.24, PLE 0.34; interdistances AME-ALE 0.31, PME-PLE 0.23, ALE-PLE 0.16, AME-PME 0.04; ocular quadrangle AME-AME 0.43, PME-PME 0.85; clypeus 0.09 high. Mouthparts:chelicerae with a few stout setae medially and anteriorly; lateral boss present, smooth; promargin with 3 teeth, retromargin with 2 teeth; maxillae longer than broad, with tuft of conspicuous setae distally; labium distally rounded. Sternum:0.94 times longer than broad, posteriorly indented. Pedipalp:femur, spination dorsal 0–1–2; retrolateral tibial apophysis with 3 processes, dorsal apophysis long, slender and slightly curved, median apophysis broad and triangular, ventral apophysis small with a squarish tip; retrolateral basal cymbial process present; cymbial scopulae absent; cymbium oval to triangular, angled bottom right; conductor large and quadrangular, with a sclerotized tip directed basally; embolus long and slender, beginning at 6 o’clock, terminating at 1 o’clock, not following edge of cymbium, but more toward the center of the bulb; MA long, slightly curved, distally spatulate, directed laterally then distally (Fig. 21). Legs:leg I only slightly shorter than legs II, III and IV; leg formula 3421; scopulae absent on all legs; tarsus I–IV with strong claw tufts; pr claws with 1 or 2 small teeth; spination: leg I, Fm pr 1–1–0, d 1–1–1, rl 0; Ti d 0, v 2–2–2–2–2; Mt 0; Ti and Mt I and II with very weak spines; leg II, Fm pr 0, d 1–1–1, rl 0; Ti v 2–2–2–2–2; Mt 0; leg III, Fm pr 0, d 1–1–1, rl 0; Ti 0; Mt 0; leg IV, Fm pr 0, d 1–1–1, rl 0; Ti 0; Mt 0. Abdomen:without terminal tufts of setae. *Dimensions:* Total length 5.61. Cephalothorax length 2.69, width 3.01. Sternum length 1.48, width 1.55. Abdomen length 3.22, width 2.98. Pedipalp: Fm 0.81, Pt 0.41, Ti 0.45, Ta 0.81, (total) 2.48. Leg I: Fm 4.16, Pt 1.41, Ti 3.89, Mt 3.28, Ta 1.45, (total) 14.19. Leg II: Fm 5.49, Pt 1.59, Ti 4.71, Mt 3.81, Ta 1.82, (total) 17.42. Leg III: Fm 6.01, Pt 1.48, Ti 4.77, Mt 4.38, Ta 1.68, (total) 18.32. Leg IV: Fm 5.47, Pt 1.19, Ti 4.42, Mt 3.89, Ta 1.61, (total) 16.58.

###### Natural history.

Collected from under rocks.

###### Distribution.

The type locality only (Map 5).

##### Karaops jarrit sp. n.

urn:lsid:zoobank.org:act:60577CDB-3D72-46C7-B7C2-E53D257311A0

http://species-id.net/wiki/Karaops_jarrit

Figs 23–26 Map 6

###### Type material.

Holotype male (WAM T55003): 11 km NW of Roe’s Rock (16A), Fitzgerald River National Park, 33°57'47"S, 119°16'39"E, Western Australia, Australia, XI.1996, A. Saunders. Paratype: conveyor #2, Worsley Alumina Overland Conveyor Belt, SW of Boddington, 33°07'43"S, 116°07'34"E, Western Australia, Australia, 8.VI.2007, J. Hynes, 1♀ (WAM T87168).

###### Other material examined.

**AUSTRALIA: Western Australia:** Duncraig, 31°50'S, 115°47'E, 17.XII.1987, D. Robinson, 1♀ (WAM T93/1329); 24.2 km WNW of Quindanning, Worsley Alumina conveyor #1, 32°59'52.6"S, 116°19'05.3"E, 30.XI.2009, J. Hynes, 1♂ (WAM T99505); 33.5 km W. of Quindanning, Worsley Alumina conveyor #1, 33°02'34.3"S, 116°12'36.9"E, 1.XII.2009, J. Hynes, 1♂ (WAM T99504); 26.8 km NW of Quindanning, Worsley Alumina conveyor #1, 33°00'38.5"S, 116°17'14.2"E, 9.XII.2009, J. Hynes, 1♂ (WAM T99759); 23.8 km NW of Quindanning, Worsley Alumina conveyor #1, 32°59'42.8"S, 116°19'28.3"E, 9.XII.2009, J. Hynes, 1♂ (WAM T99760); 24.3 km NW of Quindanning, Worsley Alumina conveyor #1, 32°59'53.3"S, 116°19'03.0"E, 9.XII.2009, J. Hynes, 1♂ (WAM T99761).

###### Etymology.

The specific name comes from the Nyoongar *jarrit*, meaning jarrah. Jarrah trees (*Eucalyptus marginata*) are common throughout the area where this species is found. The name is to be treated as a noun in apposition.

###### Diagnosis.

**Figures 23–28.**
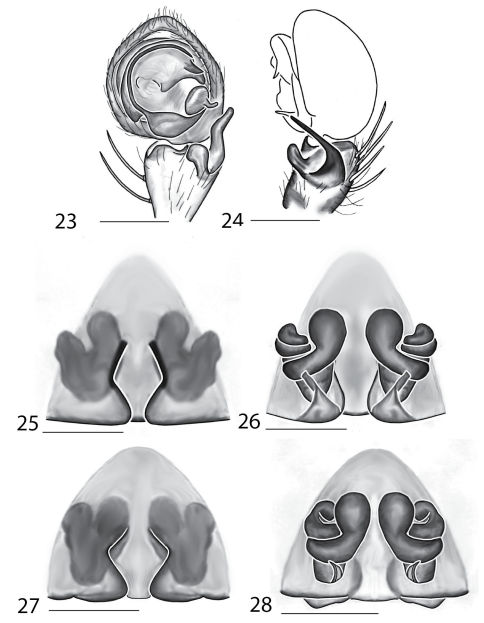
Copulatory organs of *Karaops jarrit* sp. n., male holotype from 11 km NW of Roe’s Rock, Fitzgerald River National Park, Western Australia, Australia (WAM T55003) (**23–24**), female paratype from southwest of Boddington, Western Australia, Australia (WAM T87168) (**25–26**), and *Karaops marrayagong* sp. n., female holotype from Kitty’s Creek, near Sydney, New South Wales, Australia (AM KS19743) (**27–28**) **23** male pedipalp, ventral view **24** male pedipalp, retrolateral view **25, 27** epigyne, ventral view **26, 28** spermathecae, dorsal view. Scale bar: 0.25 mm.

Males of this species can be separated from all other species except *Karaops raveni* sp. n. by having unpaired spines on Ti I and II, and can be separated from *Karaops raveni* by having the MA with a quadrangular base. Females can be separated from other species by the a diamond shaped median septum, small posterodorsal folds, and coiled internal ducts (Figs 23–26).

###### Remarks.

Though the male and female have not been collected together, it is clear from their morphologies that they are the same species. Additionally, and interestingly, this species is morphologically similar to *Karaops raveni* sp. n. and *Karaops marrayagong* sp. n. from eastern Australia. The cephalothorax of all three species is strongly flattened, giving the habitus a very truncate, or short and squat, appearance. The females have 5 paired spines on the ventral tibiae I and II, and 3 pairs on the metatarsi, whereas the male has either 4 or 5 spines (unpaired) on tibiae I and II, and no spines on the ventral surface of the metatarsi, though the male of *Karaops marrayagong* sp. n. is unknown.

###### Description.

*Male (holotype):*Color: carapace yellow-brown, with slightly darker marks laterally; sternum pale yellow-brown; chelicerae uniformly dark red-brown; maxillae pale yellow-brown; labium pale brown; abdomen dorsally yellow-brown with darker markings; ventrally pale yellow-brown; legs uniformly pale red-brown. Cephalothorax:setae long and thin, carapace strongly flattened; 0.75 times longer than broad; fovea longitudinal, broad, very shallow. Eyes:AER slightly recurved; PER recurved; PME same size as AME, PLE largest, ALE smallest; eye group width 1.81; eye diameters, AME 0.21, ALE 0.21, PME 0.12, PLE 0.3; interdistances AME-ALE 0.34, PME-PLE 0.25, ALE-PLE 0.15, AME-PME 0.05; ocular quadrangle AME-AME 0.59, PME-PME 1.08; clypeus 0.12 high. Mouthparts:chelicerae with a few stout setae medially and anteriorly; lateral boss present, smooth; promargin with 3 teeth, retromargin with 2 teeth; maxillae longer than broad, with tuft of conspicuous setae distally; labium distally rounded. Sternum:0.66 times longer than broad, posteriorly indented. Pedipalp:femur, spination dorsal 0–1–2; retrolateral tibial apophysis with 2 processes, dorsal apophysis long and slender, curved ventrally in lateral view, pointed at tip, slightly bent laterally at tip in ventral view, ventral apophysis broad and blunt, rounded to quadrangular distally; retrolateral basal cymbial process absent; cymbial scopulae absent; cymbium round to triangular, angled bottom right; conductor large, slightly crescent shaped, with slightly rounded to triangular processes, one in the center of the bulb, and two near the tip, one atop the other, the top one more pointed and directed ventrally; embolus very long and slender, arising off of a large ovoid base and tapering abruptly, beginning at 6 o’clock, terminating at 2 o’clock; MA ovoid, with a single finger-like process arising medially, directed ventrolaterally, MA only lightly sclerotized (Figs 23–24). Legs:leg I much shorter than legs II, III and IV; leg formula 2341; scopulae absent on all legs; tarsus I–IV with strong claw tufts; pr claws with c. 10–15 teeth, rl claws lacking teeth; spination: leg I, Fm pr 1–1–0, d 1–1–1, rl 0; Ti d 0, v 1–1–1–1, or d 0, v 1–1–1–1–1; Mt 0; Ti and Mt I and II with very weak spines; leg II, Fm pr 0, d 1–1–1, rl 0; Ti absent; Mt 0; leg III, Fm pr 0, d 1–1–1, rl 0; Ti 0; Mt 0; leg IV, Fm pr 0, d 1–1–1, rl 0; Ti 0; Mt 0. Abdomen:without tufts of setae, but hairs worn off. *Dimensions:* Total length 5.31. Cephalothorax length 2.61, width 3.50. Sternum length 0.66, width 1.00. Abdomen length 2.72, width 2.86. Pedipalp: Fm 0.97, Pt 0.54, Ti 0.50, Ta 0.83, (total) 2.84. Leg I: Fm 3.94, Pt 1.57, Ti 3.93, Mt 3.33, Ta 1.43, (total) 14.20. Leg II: Fm 6.67, Pt 1.87, Ti 6.24, Mt 5.96, Ta 2.02, (total) 22.76. Leg III: Fm 6.14, Pt 1.60, Ti 5.18, Mt 4.89, Ta 1.77, (total) 19.58. Leg IV: Fm 4.81, Pt 1.29, Ti 4.18, Mt 4, Ta 1.56, (total) 15.84.

*Female* (paratype): Color: carapace yellow-brown, with slightly darker marks laterally and medially; sternum pale yellow-brown; chelicerae yellow-brown; labium pale yellow-brown, lightening distally; abdomen dorsally dark grey with lighter cardiac area and lighter patches laterally and posteriorly; ventrally pale yellow-brown; legs: femora prolaterally with dark annulations connected along the length giving the appearance of dark grey femora with four yellow spots, patellae and tibiae with annulations, retrolaterally and dorsally dark, ventrally yellow to yellow-brown, metatarsus and tarsus dark. Cephalothorax: setae long and thin, carapace strongly flattened; 0.72 times longer than broad; fovea longitudinal, broad, very shallow. Eyes:AER nearly straight; PER slightly recurved; AME slightly larger than PME, PLE largest, ALE smallest; eye group width 1.53; eye diameters, AME 0.19, ALE 0.10, PME 0.17, PLE 0.29; interdistances AME-ALE 0.40, PME-PLE 0.33, ALE-PLE 0.29, AME-PME 0.08; ocular quadrangle AME-AME 0.19; clypeus 0.06 high. Mouthparts:chelicerae with a few stout setae medially and anteriorly; lateral boss present, smooth; promargin with 3 teeth, retromargin with 2 teeth; maxillae longer than broad, with tuft of conspicuous setae distally; labium distally rounded. Sternum:0.68 times longer than broad, posteriorly indented. Pedipalp:tarsus slightly swollen, claw present with less than 6 teeth. Legs:leg I much shorter than legs II, III and IV; leg formula 3241; scopulae absent on all legs; tarsus I–IV with strong claw tufts; pr claws with c. 10 teeth, rl claws with none; spination: leg I, Fm pr 1–1–0, d 1–1–1, rl 0; Ti d 0, v 2–2–2–2–2; Mt v 2–2–2; Ti and Mt I and II with strong spines; leg II, Fm pr 0, d 1–1–1, rl 0; Ti v 2–2–2–2–2; Mt v 2–2–2; leg III, Fm pr 0, d 1–1–1, rl 0; Ti 1–0–0; mt 0; leg IV, Fm pr 0, d 1–1–1, rl 0; Ti v 1–1; Mt 1–0. Abdomen:terminal setal tufts present. Epigyne:two lobes surrounding median area, giving the median field a long diamond shaped appearance, truncated posteriorly, copulatory openings located anterolaterally, at sides of median field, epigynal pockets absent, very small posterodorsal fold barely covering bottom of internal ducts, spermathecae ovoid, located anteriorly, ducts coiled, with the anterior most coil flattened, fertilization ducts located posteriorly (Figs 25–26). *Dimensions:* Total length 6.21. Cephalothorax length 2.67, width 3.70. Sternum length 1.40, width 2.06. Abdomen length 3.54, width 3.52. Pedipalp: Fm 0.94, Pt 0.57, Ti 0.61, Ta 0.84, (total) 2.06. Leg I: Fm 3.26, Ti 2.79, Mt 2.38, Ta 0.99, (total) 10.80. Leg II: Fm 4.59, Pt 1.47, Ti 3.62, Mt 2.96, Ta 1.22, (total) 13.86. Leg III: Fm 5.15, Pt 1.44, Ti 3.73, Mt 2.93, Ta 1.38, (total) 14.63. Leg IV: Fm 4.25, Pt 1.15, Ti 3.05, Mt 2.64, Ta 1.09, (total) 12.18.

###### Natural history.

Found in pitfall traps, and at night along overland conveyors.

###### Distribution.

**Map 6.**
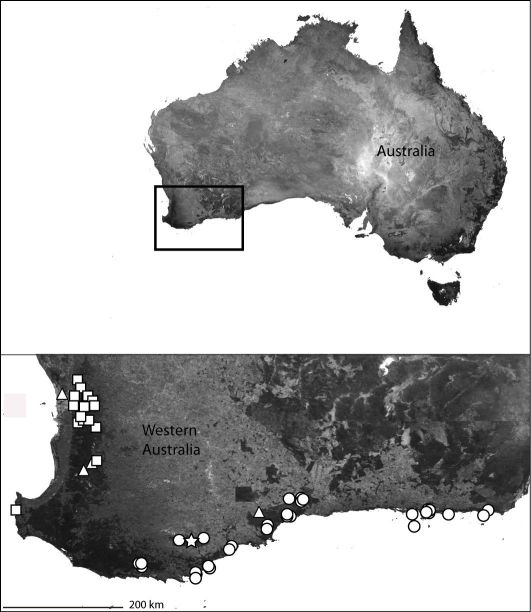
The southwest coast of Western Australia (inset) showing the distribution of *Karaops* gen. n. *Karaops francesae* sp. n. (white circles), *Karaops toolbrunup* sp. n. (white star), *Karaops jarrit* sp. n.(white triangles), and *Karaops ellenae* sp. n.(white squares).

Near the south and west coasts of southwestern Australia (Map 6).

##### Karaops marrayagong sp. n.

urn:lsid:zoobank.org:act:07EC2C05-4B21-482D-A5CE-5429DBFEE3A6

http://species-id.net/wiki/Karaops_marrayagong

Figs 27–28 Map 7

###### Type material.

Holotype female (AM KS19743): Kitty’s Creek [33°47'S, 151°08'E], near Sydney, New South Wales, Australia, 1916.

###### Etymology.

The specific epithet comes from the indigenous Dharug word for spider. Dharug is the language indigenous to the type locality. The name is to be treated as a noun in apposition.

###### Diagnosis.

This species can be separated from *Karaops raveni* sp. n. as the lateral lobes of the epigyne do not come into contact posteriorly, the ducts are narrower, and the spermathecae are closer together (Figs 27–28). Males unknown.

###### Remarks.

There is some ambiguity as to where exactly this specimen is from as it is rather old. The region of Kitty’s Creek in Sydney has been searched recently, but the area has been developed a great deal since the specimen was originally collected.

###### Description.

*Holotype:*Color: carapace uniformly yellow-brown; sternum pale yellow-brown; chelicerae yellow-brown; maxillae pale yellow-brown; labium pale brown; abdomen dorsally pale creamy-yellow with a few darker flecks; ventrally pale yellow-brown; legs uniformly pale red-brown. Cephalothorax:setae long and thin; carapace strongly flattened; 0.71 times longer than broad; fovea longitudinal, broad, very shallow. Eyes:AER nearly straight; PER slightly recurved; AME slightly larger than PME, PLE largest, ALE smallest; eye group width 1.55; eye diameters, AME 0.15, ALE 0.10, PME 0.15, PLE 0.17; interdistances AME-ALE 0.38, PME-PLE 0.36, ALE-PLE 0.27, AME-PME 0.13; ocular quadrangle AME-AME 0.21, PME-PME 0.84; clypeus 0.04 high. Mouthparts:chelicerae with a few stout setae medially and anteriorly; lateral boss present, smooth; promargin with 4 teeth, retromargin with 3 teeth; maxillae longer than broad, with tuft of conspicuous setae distally; labium distally rounded. Sternum:0.69 times longer than broad, posteriorly indented. Pedipalp:tarsus slightly swollen, claw present, with c. 6 teeth. Legs:leg I much shorter than legs II, III and IV; leg formula 3241; scopulae absent on all legs; tarsus I–IV with strong claw tufts; pr claw with less than 10 teeth, rl claws with none; spination: leg I, Fm pr 0, dorsal 1–1–1, rl 0; Ti d 0, v 2–2–2–2–2–2; Mt v 2–2–2–2; Ti and Mt I and II with strong spines; leg II, Fm pr 0, d 1–1–1, rl 0; Ti v 2–2–2–2–2; Mt v 2–2–2; leg III, Fm pr 0, d 1–1–1, rl 0; Ti 1–0–0; mt 1–0; leg IV, Fm pr 0, d 1–1–1, rl 0; Ti v 1–0–0; Mt 0. Abdomen:possible setal tufts, hairs worn off. Epigyne:two lateral lobes, forming a diamond shaped median area, copulatory openings located anteromedially, epigynal pockets absent; internally, wide ducts coil 2–3 times and lead to oval spermathecae that are close together, fertilization ducts located posteriorly, posterodorsal fold absent (Figs 27–28).

*Dimensions:* Total length 5.81. Cephalothorax length 2.77, width 3.88. Sternum length 1.43, width 2.07. Abdomen length 2.04, width 3.70. Pedipalp: Fm 0.86, Pt 0.57, Ti 0.59, Ta 0.71, (total) 2.73. Leg I: Fm 3.37, Pt 1.44, Ti 2.93, Mt 3.07, Ta 1.15, (total) 11.96. Leg II: Fm 4.68, Pt 1.72, Ti 4.07, Mt 2.82, Ta 1.32, (total) 14.61. Leg III: Fm 5.36, Pt 1.66, Ti 4.45, Mt 3.70, Ta 1.43, (total) 16.60. Leg IV: Fm 4.77, Pt 1.34, Mt 2.82, Ta 1.15, (total) 13.60.

###### Natural history.

No data.

###### Distribution.

**Map 7.**
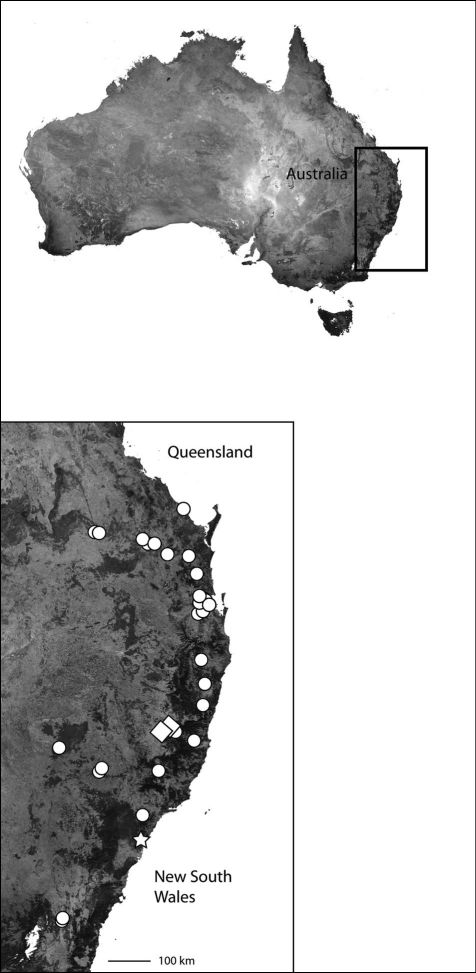
Eastern Australia (inset) showing the distribution of *Karaops* gen. n. *Karaops raveni* sp. n. (white circles), *Karaops manaayn* sp. n. (white diamonds), *Karaops marrayagong* sp. n. (white star).

The type locality only (Map 7).

##### Karaops raveni sp. n.

urn:lsid:zoobank.org:act:95307E87-23A0-41D0-B809-4686F230CD51

http://species-id.net/wiki/Karaops_raveni

**Figures 29–34.**
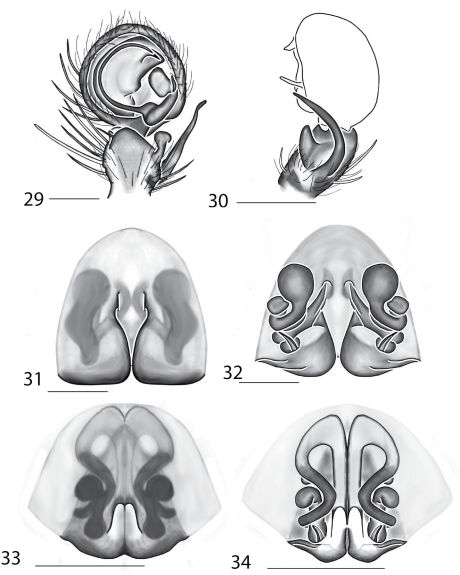
Copulatory organs of *Karaops raveni* sp. n., male holotype from Brooyar State Forest, Queensland, Australia (QM S50593) (**29–30**), female paratype from Boat Mountain EP, Queensland, Australia (QM S47057) (**31–32**), and *Karaops badgeradda* sp. n., female holotype from Badgeradda Range, Muggon Station, Western Australia, Australia (WAM T97214) (**23–34**) **29** male pedipalp, ventral view **30** male pedipalp, retrolateral view **31, 33** epigyne, ventral view **32, 34** spermathecae, dorsal view. Scale bar: 0.25 mm (**29–32**), 0.50 mm (**33–34**).

Figs 29–32 91 96–97 111 Map 7

###### Type material.

Holotype male (QM S50593):Brooyar State Forest, 26°08'S, 152°31'E, Queensland, Australia, 300 m, 16.VIII.1997, G.B. Monteith. Paratype: Boat Mountain EP, 26°10'S, 151°58'E, Queensland, Australia, 580 m, 14.XII.1994, G.B. Monteith and G. Thompson, 1♀ (QM S47057).

###### Other material examined.

**AUSTRALIA: Queensland:** Binjour Plateau [25°28'S, 151°23'E], 18.XI.2000, R. Raven, B. Baehr, 1♂ (QM S60167);Brisbane, 2.XII.1935, H.A. Longman, 1♂ (QM S61053); Bundaberg, 24°52'S, 152°21'E, 1986, E. Zillman, forest, 1♀ (QM S61056); Camira [27°38'S, 152°55'E], 1.I.1994, R. Raven, 1♂ (QM S25834); Drewvale, Illaweena Street, 27°38.5'S, 153°03.8'E, 40 m, 2.IX.2008, QM Party, scribbly gum, heath, 2♂ (QM S62456); Drewvale, Illaweena Street, 27°38.5'S, 153°03.8'E, 40 m, 5.XI.2003, QM Party, scribbly gum, heath, 1♀ (QM S66707); Gayndah, 100 km west of Maryborough [25°37'S, 151°36'E], I.2001, B. Baehr, 1♀ (QM S56867); Karawatha Forest, site 6, 27°37.6'S, 153°05.4'E, 5.XI.2003, QM Party, eucalypt woodland, 1♀ (QM S65113); Kenilworth, 25.I.1975, 1♀ (QM S47114); Nipping Gully, site 5, 25°42'S, 151°26'E, 26.I.1999, G. Monteith, vine scrub, fogging trees with pyrethrum, 1♀, 1 penultimate ♂, 3 immatures (QM S57611); Oxley [27°33'S, 152°58'E], I.1894, 1♀, 4 immatures (QM S61057); Springfield, 27°40'S, 152°55'E, 19.IX.1998, K. Walker, G. Robinson, 1♂, 1♀ (QM S42635, S42636); Stafford [27°24'S, 153°00'E], I.1910, 1♀ (QM S61055); Taroom District, Boggomoss, Price’s Creek, 25°29'S, 150°08'E, 11.IX.1996, P. Lawless, H. Janetzki, D.P., 1♀ (QM S36689); Taroom, Mount Rose Station, BM8, Boggomoss, 25°27'14"S, 150°01'45"E, 17.VI.1996, P. Lawless, H. Janetzki, J. Stanisic, G. Ingram, under bark of *Ficus* and *Eucalyptus*, 2♂, 1♀, 2 immatures (QM S37308). **New South Wales:** Armidale-Kempsey Road above MacLeay River, 30°44'52.6"S, 152°14'31.2"E, 26.II.2009, S. Crews, H. Smith, under eucalypt bark on tree along road, 1 immature (WAM T97228); Bimbadeen Lookout, Southwest of Cessnock, 21.IV.1990, D. Hirst, under bark, 1♀, 1 penultimate ♂ (SAM N199360-61); Brooklana [30°16'S, 152°51'E], E. Dorrigo, .VII.1929, W. Heron, 1♂, 1 immature (AM KS43754); Coolah Tops National Park, Grass Tree Track, 31°44'06"S, 150°00'05"E, 8.XI.2001, G. Milledge, under bark, 1♂ (AM KS75104); Coolah Tops National Park, Bald Hill Track, 2.5 km from the Forest Road, 31°45'02"S, 150°01'26"E, 8.XI.2001, G. Milledge, under bark, 1♀, 1 immature (AM KS75087); MacLean District Lower Clarence River [29°30'S, 153°12'E], 19.XI.1940, A.A. Cameron, 1♀ (AM KS43755); Richmond Range [28°20'S, 152°55'E], 16.IV.1976, R. Raven, on tin water tank, 1♀ (QM S34994); Skillion Nature Reserve, 0.7 km off Armidale-Kempsey Road near Jack’s Flat Road, 30°58'54.4"S, 152°43'24.8"E, 26.II.2009, S.C. Crews, H. Smith, under bark and on trees at sunset, 2 penultimate ♂, 6 immatures (WAM T97216, T97221, T97223, T97224, T97226, T97251, T97289, T97290); Tinderry Range, 17.8 km from Captain’s Flat Road on Tinderry Road, 35°45'01"S, 149°15'40"E, 10.XII.2005, V.W. Framenau, J.M. Waldock, under rocks on scree slope, 2♀ (WAM T67903); Tinderry Range, ESE of Michelago, 35°44'33.9"S, 149°14'57.1"E, 3.III.2009, S. Crews, A. Seago, under rocks on scree slope, 1♀, 1 penultimate ♂, 2 immatures (WAM T97217-T97219, T97231); Warrumbungles National Park, John Renshaw Parkway, 1.9 km W of camp Wambelong, 31°16'32'S, 148°57'37"E, 10.XI.2001, G. Milledge, H. Smith, M. Gray, under rocks, 2♀, 1 immature (AM KS75204); Watchimbark Nature Reserve beside Watchimbark Creek, 31°42'50"S, 151°37'44"E, 31.V.2007, H. Smith, under bark, 1♂, 1♀ (WAM T85304, T85305).

###### Etymology.

The specific epithet is in honor of Dr. Robert Raven from the Queensland Museum in recognition of his work on Australian spiders.

###### Diagnosis.

Males can be distinguished from all other males by having unpaired setae ventrally on tibiae I and II, and from *Karaops jarrit* sp. n. by having the MA with a quadrangular base (Figs 29–30). Females can be distinguished from other species by an almost downward-pointing arrow- shaped median septum and the lateral lobes coming into contact posteriorly (Figs 31–32).

###### Remarks.

Thus far, this is the most widespread species of *Karaops* gen. n. Although the copulatory organs were identical in all specimens examined, there is much morphological variation throughout the range of the species in somatic characters, and size. The female has paired tibial and metatarsal ventral spines on legs I and II, while in the male, the spines are unpaired on the tibiae and there are no spines on the metatarsi, as in *Karaops jarrit* sp. n. There also seems to be some variation in the number of cheliceral teeth present – typically the promargin has 4 teeth and the retromargin has 3 teeth, however, it may differ between the right and left sides of some specimens (e.g. L 4,2; R 4,3 or R 4,2; L 2,3).

###### Description.

*Male (holotype):*Color: carapace uniformly yellow-brown; sternum pale yellow-brown; chelicerae pale yellow with darker infuscations anteriorly and laterally; maxillae pale yellow-brown; labium pale brown; abdomen dorsally yellow-brown with darker markings; ventrally pale yellow-brown; legs with femora, patellae and tibiae I–IV clearly annulated, yellow-brown, darkening distally; annulations not encircling entire legs. Cephalothorax:setae long and thin, carapace strongly flattened; 0.73 times longer than broad; fovea longitudinal, broad, very shallow. Eyes:AER nearly straight; PER slightly recurved; AME slightly larger than PME, PLE largest, ALE smallest; eye group width 1.6; eye diameters, AME 0.20, ALE 0.12, PME 0.17, PLE 0.26; interdistances AME-ALE 0.29, PME-PLE 0.30, ALE-PLE 0.17, AME-PME 0.06; ocular quadrangle AME-AME 0.54, PME-PME 0.95; clypeus 0.06 high. Mouthparts:chelicerae with a few stout setae medially and anteriorly; lateral boss present, smooth; promargin with 3 teeth, retromargin with 2 teeth; maxillae longer than broad, with tuft of conspicuous setae distally; labium distally rounded. Sternum:0.7 times longer than broad, posteriorly indented. Pedipalp:femur, spination dorsal 0–1–2; retrolateral tibial apophysis with 2 processes, dorsal apophysis long and slender, curved ventrally, pointed at tip, ventral apophysis much shorter, rounded in lateral view, with flattened ovoid tip in ventral view; retrolateral basal cymbial process present; cymbial scopulae absent, cymbium round; conductor large and slightly angular, pointed at tip; embolus very long and slender, beginning at 6 o’clock, terminating at 1 o’clock; MA with quadrangular base, long finger-like process arising from posteriorly and curving ventrodistally, only slightly sclerotized (Figs 29–30). Legs:leg I much shorter than legs II, III and IV; leg formula 2341; scopulae absent on all legs; tarsus I–IV with strong claw tufts; pr claws with c. 10–15 teeth, rl claws with 1 or 2 teeth; spination: leg I, Fm pr 1–1–0, d 1–1–1, rl 0; Ti d 0, v 1–1–1–1–1–1; Mt 0; Ti and Mt I and II with very weak spines; leg II, Fm pr 0, d 1–1–1, rl 0; Ti v 1–1–1–1–1–1; Mt 0; leg III, Fm pr 0, d 1–1–1, rl 0; Ti 0; Mt 0; leg IV, Fm pr 0, d 1–1–1, rl 0; Ti 0; Mt 0. Abdomen:without tufts of setae. *Dimensions:* Total length 4.84. Cephalothorax length 2.34, width 3.21. Sternum length 1.25, width 1.79. Abdomen length 2.57, width 2.72. Pedipalp: Fm 0.79, Pt 0.44, Ti 0.48, Ta 0.73, (total) 2.44. Leg I: Fm 3.33, Pt 1.25, Ti 3.10, Mt 3.09, Ta 1.19, (total) 11.96. Leg II: Fm 5.60, Pt 1.46, Ti 4.99, Mt 4.13, Ta 1.51, (total) 17.69. Leg III: Fm 5.41, Pt 1.41, Ti 4.58, Mt 3.89, Ta 1.46, (total) 16.75. Leg IV: Fm 4.28, Pt 1.06, Ti 3.41, Mt 3.14, Ta 1.29, (total) 13.18.

*Female (paratype):* Color: carapace uniformly yellow-brown; sternum pale yellow-brown; chelicerae pale yellow with darker infuscations anteriorly and laterally; maxillae pale yellow-brown; labium pale brown; abdomen dorsally yellow-brown with darker markings; ventrally pale yellow-brown; legs with femora, patellae and tibiae I–IV clearly annulated, yellow-brown, darkening distally; annulations not entirely encircling legs. Cephalothorax:setae long and thin; carapace strongly flattened; 0.77 times longer than broad; fovea longitudinal, broad, very shallow. Eyes:AER nearly straight; PER slightly recurved; PME same size as AME, PLE largest, ALE smallest; eye group width 1.93; eye diameters, AME 0.21, ALE 0.13, PME 0.20, PLE 0.31; interdistances AME-ALE 0.41, PME-PLE 0.34, ALE-PLE 0.20, AME-PME 0.10; ocular quadrangle AME-AME 0.62, PME-PME 1.15; clypeus 0.08 high. Mouthparts:chelicerae with a few stout setae medially and anteriorly; lateral boss present, smooth; promargin with 4 teeth, retromargin with 2 teeth; maxillae longer than broad, with tuft of conspicuous setae distally; labium distally rounded. Sternum:0.75 times longer than broad, posteriorly indented. Pedipalp:tarsus slightly swollen, claw present, with c. 6 teeth. Legs:leg I much shorter than legs II, III and IV; leg formula 3241; scopulae absent on all legs; tarsus I–IV with strong claw tufts; pr claws with c.10–15 teeth, rl claws with 1 or 2 teeth; spination: leg I, Fm pr 1–1–0, d 1–1–1, rl 0; Ti d 0, v 2–2–2–2–2; Mt v 2–2–2; Ti and Mt I and II with strong spines; leg II, Fm pr 0, d 1–1–1, rl 0; Ti v 2–2–2–2–2; Mt v 2–2–2; leg III, Fm pr 0, d 1–1–1, rl 0; Ti 0; Mt 0; leg IV, Fm pr 0, d 1–1–1, rl 0; Ti 0; Mt 0. Abdomen:setal tufts present. Epigyne:lateral lobes, nearly touching posteriorly, forming an arrow shaped median field, epigynal pockets absent, copulatory openings located anterolaterally; internally two round spermathecae located anteriorly, ducts twisting multiple times, with anterior most coil flattened, small posterodorsal fold present, not covering any internal ducts, fertilization ducts located posteriorly (Figs 31–32). *Dimensions:* Total length 7.28. Cephalothorax length 3.02, width 3.94. Sternum length 1.55, width 2.08. Abdomen length 4.19, width 4.26. Pedipalp: Fm 0.83, Pt 0.56, Ti 0.56, Ta 0.92, (total) 2.87. Leg I: Fm 3.43, Pt 1.60, Ti 3.02, Mt 2.35, Ta 1.09, (total) 11.49. Leg II: Fm 4.94, Pt 1.71, Ti 4.07, Mt 3.11, Ta 1.40, (total) 15.23. Leg III: Fm 5.38, Pt 1.61, Ti 4.27, Mt 3.43, Ta 1.55, (total) 16.24. Leg IV: Fm 4.34, Pt 1.22, Ti 3.57, Mt 2.83, Ta 1.26, (total) 13.22.

###### Natural history:

Found under bark and under rocks, and on trees at night (Fig. 91).

###### Distribution.

Widespread in eastern Australia, from the Tinderry Range in the south of New South Wales to the Bundaberg Forest in Queensland in the north (Figs 96–97; Map 7).

##### Karaops badgeradda sp. n.

urn:lsid:zoobank.org:act:84E3F7C0-62DB-4FA7-A9DA-91D1829E20CD

http://species-id.net/wiki/Karaops_badgeradda

**Map 8.**
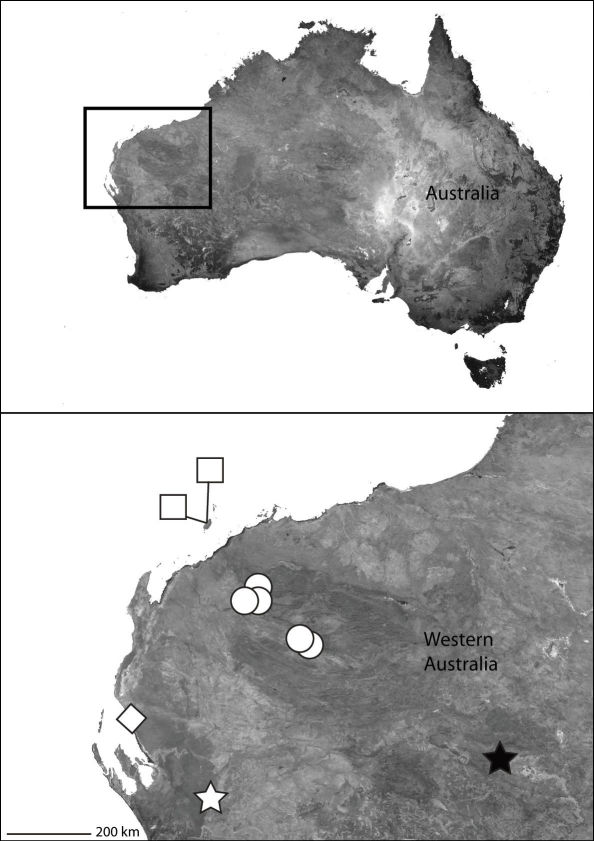
The northwest of Western Australia (inset) showing the distribution of *Karaops* gen. n. *Karaops julianneae* sp. n. (black star), *Karaops badgeradda* sp. n. (white star), *Karaops karrawarla* sp. n. (white diamond), *Karaops martamarta* sp. n.(white circles), *Karaops burbidgei* sp. n. (white squares).

Figs 33–34 92 98 Map 8

###### Type material.

Holotype female (WAM T97214): site 5, Badgeradda Range, Muggon Station, 26°46'01.6"S, 115°32'54.4"E, Western Australia, Australia, 18.III.2009, S.C. Crews, M.C. Murrmann, under rocks.

###### Other material examined.

**AUSTRALIA: Western Australia:** collected with holotype, 2♀ (WAM T97213, T97215); Muggon Station, site MUG 9, 26°49'03"S, 115°32'56"E, 20–28.X.2003, A. Desmond, M. Cowan, dry pitfall, 1 immature (WAM T95019); Muggon Station, site MUG 5, 26°46'S, 115°33'E, 20–28.X.2003, A. Desmond, M. Cowan, dry pitfall, 2 immatures (WAM T95020).

###### Etymology.

The specific epithet comes from the type locality. The name is to be treated as a noun in apposition.

###### Diagnosis.

This species can be separated from all other species by genitalic characteristics, including a bilobed sclerotized area covering the copulatory openings located in the posterior third of the epigynal plate (Fig. 33). Males unknown.

###### Remarks.

Immatures are matched with this species as they were collected from the same locality as the holotype.

###### Description.

*Holotype:*Color: carapace yellow-brown, with slightly darker marks laterally and medially; sternum pale yellow; chelicerae pale yellow with darker infuscations anteriorly and laterally; maxillae pale yellow; labium pale yellow-brown, lightening distally; abdomen dorsally reddish with darker red brown cardiac mark, many dark spots medially and laterally; ventrally pale yellow-brown; legs pale yellow, with all segments clearly annulated, though those on femur not entirely encircling it, also hollow in center, giving femora a ‘leopard spot’ appearance. Cephalothorax:setae long and thin; carapace flattened; 0.78 times longer than broad; fovea longitudinal, broad, somewhat shallow. Eyes:AER slightly recurved; PER recurved; PME larger than AME, PLE largest, ALE smallest; eye group width 1.49; eye diameters, AME 0.19, ALE 0.06, PME 0.25, PLE 0.38; interdistances AME-ALE 0.57, PME-PLE 0.33, ALE-PLE 0.38, AME-PME 0.04; ocular quadrangle AME-AME 0.19, PME-PME 0.55; clypeus 0.1 high. Mouthparts:chelicerae with a few stout setae medially and anteriorly; lateral boss present, smooth; promargin with 3 teeth, retromargin with 2 teeth; maxillae longer than broad, with tuft of conspicuous setae distally. Sternum:0.83 times longer than broad, posteriorly indented. Pedipalp:claw present with more than 6 teeth. Legs:leg I much shorter than legs II, III and IV; leg formula 3421; scopulae absent on all legs; tarsus I–IV with strong claw tufts; claws without teeth; spination: leg I, Fm pr 1–1–1, d 1–1–1, rl 0; Ti d 0, v 2–2–2–2–2; Mt v 2–2–2–2; Ti and Mt I and II with strong spines; leg II, Fm pr 0, dorsal 1–1–1, rl 0; Ti v 2–2–2–2–2; Mt v 2–2–2–2; leg III, Fm pr 0, d 1–1–1, rl 0; Ti v 1–1–0; Mt 2–0; leg IV, Fm pr 0, d 1–1–1, rl 0; Ti v 1–0; Mt 0. Abdomen:terminal setal tufts present. Epigyne:lateral lobes fused, small medial depression in lower third of plate, bilobed sclerotized hood, epigynal pockets absent, copulatory openings located under hood; copulatory ducts in contact medially, then both curve outward, then back inward becoming darker and more sclerotized, then outward again before twisting around to the small ovoid, folded spermathecae, fertilization ducts located posteriorly, small posterodorsal fold present, not covering any part of the internal genital (Figs 33–34). *Dimensions:* Total length 7.33. Cephalothorax length 2.89, width 3.52. Sternum length 1.57, width 1.90. Abdomen length 4.44, width 3.88. Pedipalp: Fm 0.86, Pt 0.46, Ti 0.54, Ta 0.94, (total) 2.80. Leg I: Fm 3.51, Pt 1.29, Ti 2.99, Mt 2.77, Ta 1.20, (total) 11.76. Leg II: Fm 4.44, Pt 1.56, Ti 3.22, Mt 3.08, Ta 1.27, (total) 13.57. Leg III: Fm 4.77, Pt 1.51, Ti 3.73, Mt 3.52, Ta 1.39, (total) 14.92. Leg IV: Fm 4.68, Pt 1.53, Ti 3.44, Mt 3.03, Ta 1.53, (total) 14.21.

###### Natural history.

Collected under rocks (Figs 92, 98).

###### Distribution.

This species is only known from the Muggon Station area of Western Australia (Fig. 98; Map 8).

##### Karaops burbidgei sp. n.

urn:lsid:zoobank.org:act:F77F5868-C184-44B4-935D-BDE163C673B7

http://species-id.net/wiki/Karaops_burbidgei

Figs 35–38 Map 8

###### Type material.

Holotype male (WAM T55000): John Wayne Country, Barrow Island, Western Australia, Australia, 20°45'08"S, 115°22'05"E, 4.XI.-2.XII.1993, M.S. Harvey, J.M. Waldock, pitfall, rocky site. Paratype: Barrow Island, site 45, 20°47'18"S, 115°28'31"E, 24.IV.2005, K. Edward, S. Callan, hand collecting at night, 1♀ (WAM T76698).

###### Other material examined.

**AUSTRALIA:** **Western Australia:** Barrow Island, Gorgon Project footprint plot GP6, 20°47'05"S, 115°26'28"E, 6.V.2006, S. Callan, R. Graham, high limestone flats, at night, 1 immature (WAM T97863); Barrow Island, Plot N10, 20°49'23"S, 115°22'21"E, 6.V.2006, S. Callan, R. Graham, evaporation pits, at night, 1 immature (WAM T97682); Barrow Island, Plot N09, 20°47'05"S, 115°23'38"E, 6.V.2006, S. Callan, R. Graham, central processing facility, at night, 1 immature (WAM T97681).

###### Etymology.

This species is named for Andrew Burbidge in recognition of his conservation activities in Australia.

###### Diagnosis.

**Figures 35–42.**
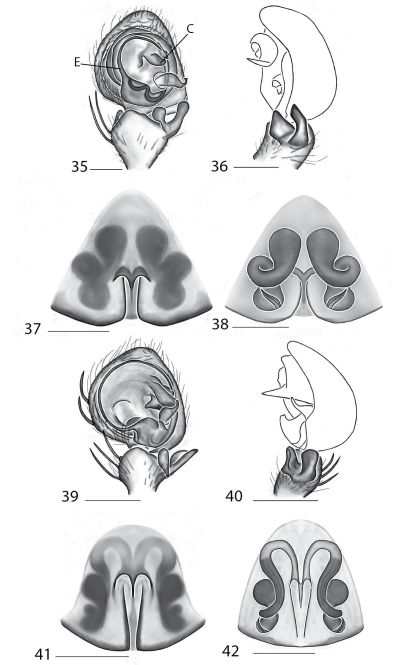
Copulatory organs of *Karaops burbidgei* sp. n., male holotype from John Wayne Country, Barrow Island, Western Australia, Australia (WAM T55000) (**35–36**), female paratype from Barrow Island, site 45, Western Australia, Australia (WAM T76698) (**37–38**), and *Karaops karrawarla* sp. n., male holotype from BB3, Bush Bay, Western Australia, Australia (WAM T55001) (**39–40**), female paratype from Bush Bay, Western Australia, Australia (WAM T76700) **35, 39** male pedipalp, ventral view **36, 40** male pedipalp, retrolateral view **37, 41** epigyne, ventral view **38, 42** spermathecae, dorsal view. Scale bar: 0.25 mm (**35–38**), 0.50 mm (**39–42**). Abbreviation: **C** = conductor.

Males of this species can be differentiated from other species by their large, transparent, laterally directed MA (Fig. 35). Females can be differentiated from other species by their medially located copulatory openings, and short internal ducts (Figs 37–38).

###### Description.

*Male (holotype):* Color: carapace uniformly yellow-brown; sternum pale yellow-brown; chelicerae pale yellow with darker infuscations anteriorly; maxillae pale yellow-brown; labium pale brown; abdomen missing; ventrally pale yellow-brown; legs with femorae, tibiae and tip of tarsi lightly annulated. Cephalothorax: setae long and thin; carapace strongly flattened; 1.14 times longer than broad; fovea longitudinal, broad, very shallow. Eyes:AER slightly recurved; PER slightly recurved; PME larger than AME, PLE largest, ALE smallest; eye group width 0.96; eye diameters, AME 0.13, ALE 0.08, PME 0.19, PLE 0.29; interdistances AME-ALE 0.29, PME-PLE 0.21, ALE-PLE 0.19, AME-PME 0.02; ocular quadrangle AME-AME 0.10, PME-PME 0.36; clypeus 0.12 high. Mouthparts:chelicerae with a few stout setae medially and anteriorly; lateral boss present, smooth; promargin with 3 teeth, retromargin with 2 teeth; maxillae longer than broad, with tuft of conspicuous setae distally; labium distally rounded. Sternum:0.72 times longer than broad, posteriorly indented. Pedipalp:femur, spination dorsal 0–1–1; retrolateral tibial apophysis with 2 processes, dorsal apophysis longer, narrower, angular, ventral apophysis quadrangular in lateral view, tip dilated in ventral view; retrolateral basal cymbial process absent; cymbial scopulae absent, cymbium oval to triangular, angled bottom right; conductor large with two processes at the tip, one fleshy and amorphous, the more anterior one quadrangular and more sclerotized; embolus long and slender, arising from large quadrangular base that abruptly narrows, beginning at 7 o’clock, terminating at two o’clock; MA long, basally thick, narrowing abruptly distally and curving distally, spatulate at tip, directed laterally (Figs 35–36). Legs:leg formula unknown (at least one leg missing); scopulae absent; tarsi with strong claw tufts; claws without teeth; spination: leg I, Fm pr 1–1–1, d 1–1–1, rl 0; Ti d 0, v 2–2–2–2–2; Mt v 2–2–2–2; Ti and Mt I and II with strong spines; II, Fm pr 0–0–1, d 1–1–1, rl 0; Ti v 2–2–2–2–2; Mt v 2–2–2–2; IV, Fm pr 0–0–1, dorsal 1–1–1, rl 0; Ti v 2–2; Mt 0. *Dimensions:* cephalothorax length 1.99, width 1.74. Sternum length 0.96, width 1.34. Pedipalp: Fm 0.67, Pt 0.38, Ti 0.38, Ta 0.71, (total) 2.14. Leg I: Fm 2.57, Pt 1.01, Ti 2.12, Mt 2.05, Ta 1.07, (total) 8.82. Leg II: Fm 3.22, Pt 1.05, Ti 2.62, Mt 2.30, Ta 1.15, (total) 10.34. Leg III: Missing. Leg IV: Fm 3.59, Pt 0.96, Ti 2.77, Mt 2.55, Ta 1.21, (total) 11.08.

*Female* (paratype):Color: carapace uniformly yellow-brown; sternum pale yellow-brown; chelicerae pale yellow with darker infuscations anteriorly; maxillae pale yellow-brown; labium pale yellow-brown; abdomen dorsally pale creamy-yellow with a few darker flecks; ventrally pale yellow-brown; legs with segments clearly annulated, but annulations do not completely encircle femorae, legs darkening distally at tibiae; annulations lighter in centers giving a ‘leopard spot’ appearance. Cephalothorax:setae long and thin; carapace flattened; 0.9 times longer than broad; fovea longitudinal, broad, very shallow. Eyes:AER nearly straight; PER recurved; PME larger than AME, PLE largest, ALE smallest; eye group width 1.35; eye diameters, AME 0.13, ALE 0.05, PME 0.19, PLE 0.29; interdistances AME-ALE 0.24, PME-PLE 0.25, ALE-PLE 0.20, AME-PME 0.03; ocular quadrangle AME-AME 0.40, PME-PME 0.74; clypeus 0.14 high. Mouthparts:chelicerae with a few stout setae medially and anteriorly; lateral boss present, smooth; promargin with 3 teeth, retromargin with 2 teeth; maxillae longer than broad, with tuft of conspicuous setae distally; labium distally rounded. Sternum:0.93 times longer than broad, posteriorly indented. Pedipalp:tarsus slightly swollen, claw present, with 3 teeth, widely separated. Legs:leg I only slightly shorter than legs II, III and IV; leg formula 3241; scopulae absent on all legs; tarsus I–IV with strong claw tufts; pr claw with 1 or 2 small teeth; spination: leg I, Fm pr 1–1–1, d 1–1–1, rl 0; Ti d 0, v 2–2–2–2–2; Mt v 2–2–2–2; Ti and Mt I and II with strong spines; leg II, Fm pr 0–0–1, d 1–1–1, rl 0; Ti v 2–2–2–2–2; Mt v 2–2–2–2; leg III, Fm pr 0–0–1, d 1–1–1, rl 0; Ti 0; Mt 0; leg IV, Fm pr 0–0–1, d 1–1–1, rl 0–0–1; Ti 0; Mt 0. Abdomen:without tufts of setae. Epigyne:lateral lobes separated posteriorly, nearly touching; bilobed sclerotized hood in the middle of plate, copulatory openings located under hood; epigynal pockets absent; internally, two ovoid spermathecae, located anteriorly, close together, sperm ducts twisted once, fertilization ducts located posteriorly, posterodorsal fold absent (Figs 37–38). *Dimensions:* Total length 5.41. Cephalothorax length 2.20, width 2.44. Sternum length 1.23, width 1.32. Abdomen length 3.48, width 3.07. Pedipalp: Fm 0.73, Pt 0.70, Ti 0.44, Ta 0.67, (total) 2.54. Leg I: Fm 2.18, Pt 0.96, Ti 1.89, Mt 1.51, Ta 0.83, (total) 7.37. Leg II: Fm 2.61, Pt 1.04, Ti 2.20, Mt 1.71, Ta 0.94, (total) 8.50. Leg III: Fm 3.05, Pt 0.94, Ti 2.25, Mt 1.81, Ta 0.96, (total) 9.01. Leg IV: Fm 2.71, Pt 0.87, Ti 1.89, Mt 1.77, Ta 0.91, (total) 8.15.

###### Natural history.

Collected from on/under rocks at night, or in pitfall traps around rocks.

###### Distribution.

Known only from Barrow Island, Western Australia (Map 8).

##### Karaops karrawarla sp. n.

urn:lsid:zoobank.org:act:D0E97EC7-327C-474A-89DD-E6F2C66AA604

http://species-id.net/wiki/Karaops_karrawarla

Figs 39–42 Map 8

###### Type material.

Holotype male (WAM T55001): Bush Bay, site BB3, 25°04'40"S, 113°42'37"E, Western Australia, Australia, 16.I-23.V.1995, P.L. West et al., pitfall. Paratype: 1 female, same data as holotype (WAM T76700).

###### Etymology.

The specific name comes from the Yinggarda word *karrawarla*, meaning spider in the language of the indigenous Yinggarda people of the region. This name is to be treated as a noun in apposition.

###### Diagnosis.

Males can be differentiated from other species by having a palpal conductor with a long, fleshy terminal process (Fig. 39). Females can be distinguished from other species by having distinct lateral lobes, and a bilobed sclerotized area covering the copulatory openings in the upper third of the epigynal plate (Fig. 41).

###### Description.

*Male (holotype):*Color: carapace uniformly yellow-brown; sternum pale yellow-brown; chelicerae pale yellow with darker infuscations anteriorly; maxillae pale yellow-brown; labium pale brown; abdomen dorsally yellow-brown with red-brown markings; ventrally pale yellow-brown; legs with femora, patellae and tibiae I–IV clearly annulated, yellow-brown, darkening distally; annulations not entirely encircling legs. Cephalothorax:setae long and thin; 0.89 times longer than broad; fovea longitudinal, broad, very shallow. Eyes:AER nearly straight; PER recurved; PME larger than AME, PLE largest, ALE smallest; eye group width 1.45; eye diameters, AME 0.20, ALE 0.11, PME 0.24, PLE 0.31; interdistances AME-ALE 0.28, PME-PLE 0.24, ALE-PLE 0.16, AME-PME 0.02; ocular quadrangle AME-AME 0.46, PME-PME 0.86; clypeus 0.15 high. Mouthparts:chelicerae with a few stout setae medially and anteriorly; lateral boss present, smooth; promargin with 3 teeth, retromargin with 2 teeth; maxillae longer than broad, with tuft of conspicuous setae distally; labium distally rounded. Sternum:0.79 times longer than broad, posteriorly indented. Pedipalp:femur, spination dorsal 0–1–2; retrolateral tibial apophysis with 2 processes, dorsal apophysis quadrangular, slightly rounded at tip, directed laterally, ventral apophysis narrower, rounded at tip; retrolateral basal cymbial process absent; cymbial scopulae absent, cymbium round, angled bottom right; conductor large with long fleshy terminal process over a more angled process; embolus very long and slender, arising from a wide base that narrows abruptly, beginning at 6 o’clock, terminating at 3 o’clock; MA with a wide base that narrows to a small hook, directed distally (Figs 39–40). Legs:leg I only slightly shorter than legs II, III and IV; leg formula 2341; scopulae absent on all legs; tarsus I–IV with strong claw tufts; claws without teeth; spination: leg I, femur prolateral 1–1–1, dorsal 1–1–1, retrolateral 1–1–0; tibia dorsal 1–1–0, ventral 2–2–2–2–2; metatarsus ventral 2–2–2–2; tibiae and metatarsi I and II with strong spines; leg II, femur prolateral 0–1–1, dorsal 1–1–1, retrolateral 0; tibia ventral 2–2–2–2–2; metatarsus ventral 2–2–2–2; leg III, femur prolateral 0–1–1, dorsal 1–1–1, retrolateral 0; tibia 0; metatarsus 0; leg IV, femur prolateral 0–1–1, dorsal 1–1–1, retrolateral 0–1–1; tibia prolateral 1–0–1, ventral 2–2–0, retrolateral 0–1–1; metatarsus 0. Abdomen:possible setal tufts, setae worn off. *Dimensions:* Total length 4.78. Cephalothorax length 2.40, width 2.70. Sternum length 1.25, width 1.59. Abdomen length 2.91, width 2.10. Pedipalp: Fm 0.79, Pt 0.67, Tb 0.51, Tr 1, (total) 2.97. Leg I: Fm 3.43, Pt 1.31, Tb 3.24, Mt 2.99, Tr 1.49, (total) 12.46. Leg II: Fm 4.27, Pt 1.28, Tb 3.73, Mt 3.54, Tr 1.68, (total) 14.50. Leg III: Fm 4.58, Pt 1.31, Tb 3.22, Mt 3.62, Tr 1.70, (total) 14.43. Leg IV: Fm 4.06, Pt 1.02, Tb 3.44, Mt 3.35, Tr 1.62, (total) 13.49.

*Female (paratype):* Color: carapace uniformly yellow-brown; sternum pale yellow-brown; chelicerae pale yellow with darker infuscations anteriorly; maxillae pale yellow-brown; labium pale brown, lightening distally; abdomen dorsally yellow-brown with red-brown markings; ventrally pale yellow-brown; legs with segments clearly annulated, but annulations do not completely encircle femorae, legs darkening distally at tibiae; annulations lighter in centers giving a ‘leopard spot’ appearance. Cephalothorax:setae long and thin; 0.85 times longer than broad; fovea longitudinal, broad, very shallow. Eyes:AER nearly straight; PER slightly recurved; PME larger than AME, PLE largest, ALE smallest; eye group width 1.74; eye diameters, AME 0.18, ALE 0.09, PME 0.26, PLE 0.35; interdistances AME-ALE 0.38, PME-PLE 0.32, ALE-PLE 0.25, AME-PME 0.05; ocular quadrangle AME-AME 0.49, PME-PME 0.98; clypeus 0.22 high. Mouthparts:chelicerae with a few stout setae medially and anteriorly; lateral boss present, smooth; promargin with 3 teeth, retromargin with 2 teeth; maxillae longer than broad, with tuft of conspicuous setae distally; labium distally rounded. Sternum:0.92 times longer than broad, posteriorly indented. Pedipalp:tarsus slightly swollen, claw present with more than 6 teeth. Legs:leg I only slightly shorter than legs II, III and IV; leg formula 3241; scopulae absent on all legs; tarsus I–IV with strong claw tufts; claws without teeth; spination: leg I, Fm prolateral 1–1–1, d 1–1–1, rl 1–1–0; Ti d 0, v 2–2–2–2–2; Mt v 2–2–2–2; Ti and Mt I and II with strong spines; leg II, Fm pr 0–0–1, d 1–1–1, rl 0; Ti v 2–2–2–2–2; Mt v 2–2–2–2; leg III, Fm pr 0–0–1, d 1–1–1, rl 0; Ti 0; Mt 0; leg IV, Fm pr 0–0–1, d 1–1–1, rl 0; Ti 0; Mt 0. Abdomen:terminal setal tufts present. Epigyne:lateral lobes nearing each other posteriorly, bilobed hood in upper third of plate, copulatory openings located under this hood, epigynal pockets absent; internally sperm ducts directed anteriorly, then curve posteriorly becoming more sclerotized and darker, twisting outward to round spermathecae, fertilization ducts located posteriorly, posterodorsal fold absent (Figs 41–42). *Dimensions:* Total length 6.76. Cephalothorax length 3.02, width 3.54. Sternum length 1.56, width 1.70. Abdomen length 4.37, width 3.85. Pedipalp: Fm 0.94, Pt 0.64, Ti 0.28, Ta 0.46, (total) 2.32. Leg I: Fm 3.33, Pt 1.44, Ti 3.02, Mt 2.70, Ta 1.32, (total) 11.81. Leg II: Fm 4.47, Pt 1.51, Ti 3.73, Mt 3.12, Ta 1.39, (total) 14.22. Leg III: Fm 4.48, Pt 1.50, Ti 4.01, Mt 3.39, Ta 1.46, (total) 14.84. Leg IV: Fm 4.16, Pt 1.31, Ti 3.21, Mt 2.97, Ta 1.46, (total) 13.11.

###### Natural history.

No data.

###### Distribution.

The type locality only (Map 8).

##### Karaops julianneae sp. n.

urn:lsid:zoobank.org:act:15499C94-E1D4-48EB-92E9-EB7F637C8158

http://species-id.net/wiki/Karaops_julianneae

Figs 43–44 Map 8

###### Type material.

Holotype female (WAM T64748): quadrat 19, Lorna Glen Station, 26°00'05"S, 121°33'48"E, Western Australia, Australia, 1–7.XI.2002, M.A. Cowan, pitfall traps.

###### Other material examined:

**Australia: Western Australia:** Same data as holotype, 1♀, 3 immatures (WAM T107714); 1♀, 3 immatures, same data as holotype except 5–14.IV.2002 (WAM T65383); 1 immature, same data as holotype except 13–19.III.2003 (WAM T107715).

###### Etymology.

The specific epithet is in honor of Julianne Waldock for all of her work on Australian terrestrial invertebrates.

###### Diagnosis.

**Figures 43–50.**
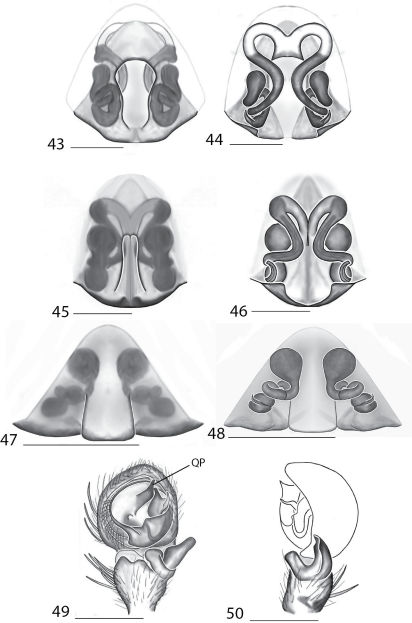
Copulatory organs of *Karaops julianneae* sp. n., male holotype from Lorna Glen Station, Western Australia, Australia (WAM T64748) (**43–44**), *Karaops martamarta* sp. n., female holotype from Trinity Bore South, vic. Cardo Camp, Red Hill, Pilbara, Western Australia, Australia (WAM T97482) (**45–46**), and *Karaops manaayan* sp. n., female holotype from Kempsey Road, West Armidale, above Mcleay River, New South Wales, Australia (AM KS043756) (**47–48**), male paratype from Armidale-Kempsey Road, above Mcleay River, New South Wales, Australia (AM KS113351) (**49–50**), **43, 45, 47** epigyne, ventral view **44, 46, 48** spermathecae, dorsal view **49** male pedipalp, ventral view **50** male pedipalp, retrolateral view. Scale bar: 0.50 mm. Abbreviation: **QP** = quadrangular process.

Females of this species can be differentiated from others by a well-defined keyhole-shaped median field, and a single genital opening that internally is divided into two copulatory ducts leading to medium-sized oval spermathecae (Figs 43–44). Males unknown.

###### Description.

*Holotype:* Color: carapace uniformly yellow-brown; sternum pale yellow-brown; chelicerae yellow-brown; maxillae pale yellow-brown, lightening distally; labium pale yellow-brown, lightening distally; abdomen dorsally yellow-brown with darker markings; ventrally pale yellow-brown; legs with femora, patellae and tibiae I-IV clearly annulated, yellow-brown, darkening distally; annulations not entirely encircling legs. Cephalothorax:setae long and thin; 0.81 times longer than broad; fovea longitudinal, broad, very shallow. Eyes:AER slightly recurved; PER recurved; PME larger than AME, PLE largest, ALE smallest; eye group width 1.43; eye diameters, AME 0.15, ALE 0.13, PME 0.27, PLE 0.33; interdistances AME-ALE 0.34, PME-PLE 0.29, ALE-PLE 0.31, AME-PME 0.04; ocular quadrangle AME-AME 0.52, PME-PME 0.98; clypeus 0.19 high. Mouthparts:chelicerae with a few stout setae medially and anteriorly, lateral boss present, smooth; promargin with 3 teeth, retromargin with 2 teeth; maxillae longer than broad, with tuft of conspicuous setae distally; labium distally rounded. Sternum:0.76 times longer than broad, posteriorly indented. Pedipalp:tarsus slightly swollen, claw present, with c. 6 teeth. Legs:leg I much shorter than legs II, III and IV; leg formula 3421; scopulae absent on all legs; tarsus I–IV with strong claw tufts; claws without teeth; spination: leg I, Fm pr 1–1–1, d 1–1–1, rl 0; Ti d 0, v 2–2–2–2–2; Mt v 2–2–2–2; Ti and Mt I and II with strong spines; leg II, Fm pl 0–1–1, d 1–1–1, rl 0; Ti v 2–2–2–2–2; Mt v 2–2–2–2; leg III, Fm pr 0–0–1, d 1–1–1, rl 0; Ti v 1–1–0; Mt 2–0; leg IV, Fm pr 0–0–1, d 1–1–1, rl 0–0–1; Ti v 1–1; Mt 2–0–0. Abdomen: terminal setal tufts present. Epigyne:lateral lobes fused forming a keyhole shaped excavated median area, copulatory openings located at the top of the medial area, epigynal pockets absent; internally, long ducts curving anterolaterally, then medioposteriorly becoming more sclerotized and darker, then posterolaterally, and back anteriorly to ovoid spermathecae, fertilization ducts located posteriorly, very small posterodorsal fold laterally (Figs 43–44). *Dimensions:* Total length 6.54. Cephalothorax length 2.84, width 3.52. Sternum length 1.43, width 1.89. Abdomen length 4.07, width 3.36. Pedipalp: Fm 0.87, Pt 0.57, Ti 0.63, Ta 0.98, (total) 3.05. Leg I: Fm 3.44, Pt 1.32, Ti 3.05, Mt 2.64, Ta 1.17, (total) 11.62. Leg II: Fm 3.81, Pt 1.43, Ti 3.52, Mt 2.96, Ta 1.26, (total) 12.98. Leg III: Fm 4.63, Pt 1.44, Ti 3.66, Mt 3.11, Ta 1.38, (total) 14.22. Leg IV: Fm 4.30, Pt 1.20, Ti 3.29, Mt 3.14, Ta 1.25, (total) 13.18.

###### Natural history.

No data.

###### Distribution.

The type locality only (Map 8).

##### Karaops martamarta sp. n.

urn:lsid:zoobank.org:act:ABD0F693-286D-4D6C-B819-441B2B7B62C7

http://species-id.net/wiki/Karaops_martamarta

Figs 45–46 93 99 Map 8

###### Type material.

Holotype female (WAM T97482): Trinity Bore South TBRC078, vicinity of Cardo Camp, Red Hill, Pilbara, Western Australia, Australia, 22°23'54.89"S, 116°19'32.43"E, 14.V.2009, S.C. Crews.

###### Other material examined.

**AUSTRALIA: Western Australia.** Pilbara, Red Hill, vicinity of Cardo Camp, Cardo Bore East, 22°11'57.67"S, 116°12'00.69"E, 15.V.2009, S.C. Crews, under rocks, 1♀ (WAM T97484); Hamersley Range, Western Ranges, c. 12 km NW. of Mt Sylvia, 23°12'15.2"S, 117°31'01.0"E, 21.VI.2009, M. Greenham, A. Johnsen, D. Kamian, 1♀, 6 immatures (WAM T97939-97942, T97944-T97946); Hamersley Range, Western Ranges, c. 22 km WNW. of Paraburdoo, 23°11'04.2"S, 117°27'29.1"E, 20.VI.2009, M. Greenham, 1 immature (WAM T97943); 19.8 km WNW Mount Berry, site WYE 10, 22°25'47.9"S, 116°16.47.3"E, 9.IX.2003–10.X.2004, CALM Pilbara Survey, ethylene glycol pit trap, 1♀ (abdomen only), 1 immature (WAM T94997).

###### Etymology.

The specific name comes from the Kurrama word *martamarta*, meaning red in the language of the indigenous Kurrama people of the region, and refers to the red color of this species (Fig 93). The name is to be treated as a noun in apposition.

###### Diagnosis.

Females of this species can be differentiated from all others by the indistinct median septum and lateral lobes, and the median field being a long, narrow depression, with round spermathecae, and the sperm ducts nearly touch medially (Figs 45–46). Male is unknown.

###### Description.

*Holotype:*Color: carapace red-brown, darker marks laterally and mediolaterally; sternum red-brown, darker around border; chelicerae red-brown with darker infuscations anteromedially to laterally; maxillae red-brown, lightening distally; labium red-brown, lightening distally; abdomen dorsally reddish with darker red brown cardiac mark, many dark spots medially and laterally; ventrally pale reddish; legs light reddish, darkening distally, annulations clearly visible, not entirely encircling legs, with open center on femorae and tibiae giving a ‘leopard spot’ appearance, with red hairs. Cephalothorax:setae long and thin; 0.87 times longer than broad; fovea longitudinal, broad, very shallow. Eyes:AER slightly recurved; PER recurved; PME larger than AME, PLE largest, ALE smallest; eye group width 1.4; eye diameters, AME 0.75, ALE 0.08, PME 0.21, PLE 0.33; interdistances AME-ALE 0.38, PME-PLE 0.36, ALE-PLE 0.29, AME-PME 0.06; ocular quadrangle AME-AME 0.17, PME-PME 0.34; clypeus 0.1 high. Mouthparts:chelicerae with a few stout setae medially and anteriorly; lateral boss present, smooth; promargin with 3 teeth, retromargin with 2 teeth; maxillae longer than broad, with tuft of conspicuous setae distally; labium distally rounded. Sternum: 0.83 times longer than broad, posteriorly indented. Pedipalp:tarsus not swollen, claw present with more than 6 teeth. Legs:leg I much shorter than legs II, III and IV; leg formula 3421; scopulae absent on all legs; tarsus I–IV with strong claw tufts; claws without teeth; spination: leg I, Fm pr 1–1–1, d 1–1–1, rl 1–1–0; Ti d 0, v 2–2–2–2–2; Mt v 2–2–2–2; Ti and Mt I and II with strong spines; leg II, Fm pr 0, d 1–1–1, rl 0; Ti v 2–2–2–2–2–2; Mt v 2–2–2–2; leg III, Fm pr 0, d 1–1–1, rl 0; Ti v 1–1–0; Mt 2–0; leg IV, Fm pr 0, d 1–1–1, rl 0; Ti v 1–1; Mt 2–0–0. Abdomen:terminal setal tufts present. Epigyne:lateral lobes fused, median excavation extending more than halfway up plate, slightly bilobed at anterior of excavation, copulatory openings located under this, epigynal pockets absent; internally, sperm ducts directed anteriorly, then laterally, becoming darker and more sclerotized, curving inward, then back laterally at almost a 90 degree angle before twisting around to large round spermathecae, fertilization ducts located posteriorly, small posterodorsal fold present, covering a small portion of the internal copulatory organs (Figs 45–46). *Dimensions:* Total length 5.90. Cephalothorax length 2.79, width 3.21. Sternum length 1.43, width 1.72. Abdomen length 3.11, width 2.89. Pedipalp: Fm 0.96, Pt 0.52, Ti 0.57, Ta 0.82, (total) 2.87. Leg I: Fm 2.93, Pt 1.24, Ti 2.52, Mt 2.01, Ta 1.24, (total) 9.94. Leg II: Fm 3.55, Pt 1.34, Ti 2.64, Mt 2.30, Ta 1.24, (total) 11.07. Leg III: Fm 3.70, Pt 1.32, Ti 2.89, Mt 2.52, Ta 1.24, (total) 11.77. Leg IV: Fm 3.52, Pt 1.24, Ti 2.64, Mt 2.64, Ta 1.28, (total) 11.32.

###### Natural history.

Collected from under rocks (Fig. 99) and in pitfall traps.

###### Distribution.

Found in the Pilbara region of Western Australia (Fig. 99; Map 8).

##### Karaops manaayn sp. n.

urn:lsid:zoobank.org:act:FE547E2C-AC98-43ED-92FF-0904CEB24348

http://species-id.net/wiki/Karaops_manaayn

Figs 47–50 96 Map 7

###### Type material.

Holotype female (AM KS43756): Kempsey Road, West of Armidale, above Macleay River [30°39'S, 152°12'E], New South Wales, Australia, 15.IV.1992, J. Frazier, on rock wall above river. Paratype: Armidale-Kempsey Road above Macleay River, 30°44'52.6"S, 152°14'31.2"E, New South Wales, Australia, 26.II.2009, S.C. Crews, H. Smith, under bark, 1♂ (AM KS113351).

###### Etymology.

The specific epithet comes from the word for spider, *manaayn*, in the indigenous language of the Dhangatti people that inhabit the Macleay Valley. The name is to be treated as a noun in apposition.

###### Diagnosis.

Females can be differentiated from all other species by having a large quadrangular medium septum (Fig. 47). Males can be differentiated from all other species by having a very large MA that is partially covered by the base of the embolus, as well as a quadrangular projection terminally on the conductor (Fig. 49).

###### Description.

*Female (holotype).*Color: carapace yellow-brown, with slightly darker marks laterally and medially; sternum pale yellow-brown; chelicerae uniformly dark red-brown; maxillae pale yellow-brown; labium pale yellow-brown; abdomen dorsally yellow-brown with red-brown and grey markings; ventrally pale yellow-brown; legs with femora, patellae and tibiae I-IV clearly annulated, yellow-brown, darkening distally; annulations not encircling legs entirely. Cephalothorax:carapace strongly flattened; setae medium length and thickness; 0.8 times longer than broad; fovea longitudinal, broad, very shallow. Eyes:AER nearly straight; PER slightly recurved; PME larger than AME, PLE largest, ALE smallest; eye group width 1.72; eye diameters, AME 0.23, ALE 0.17, PME 0.27, PLE 0.31; interdistances AME-ALE 0.42, PME-PLE 0.40, ALE-PLE 0.15, AME-PME 0.1; ocular quadrangle AME-AME 0.25, PME-PME 0.86; clypeus 0.11 high. Mouthparts:chelicerae with a few stout setae medially and anteriorly; lateral boss present, smooth; promargin with 3 teeth, retromargin with 2 teeth; maxillae longer than broad, with tuft of conspicuous setae distally; labium distally rounded. Sternum:0.84 times longer than broad, posteriorly indented. Pedipalp:tarsus slightly swollen, claw present, with c. 6 teeth. Legs:leg I much shorter than legs II, III and IV; leg formula 3241; scopulae absent on all legs; tarsus I–IV with strong claw tufts; claws prolateral claw with c. 10 teeth, retrolateral claw with similar number, but teeth are shorter; spination: leg I, Fm pr 1–1–0, d 1–1–1, rl 0; Ti d 0, v 2–2–2–2–2; Mt v 2–2–2–2; Ti and Mt I and II with strong spines; leg II, Fm pr 0, d 1–1–1, rl 0; Ti v 2–2–2–2–2–2; Mt 2–2–2–2–2; leg III, Fm pr 0, d 1–1–1, rl 0; Ti v 1–1–0; Mt 2–0; leg IV, Fm pr 0, d 1–1–1, rl 0; Ti v 1–0–0; Mt 0. Abdomen:terminal setal tufts present. Epigyne:large quadrate median septum separating lateral lobes, copulatory openings located anterolaterally to septum, epigynal pockets absent; internally, ducts coil upward to large round spermathecae, and downward to posteriorly located fertilization ducts, posterodorsal fold absent (Figs 47–48). *Dimensions:* Total length 7.70. Cephalothorax length 3.54, width 4.44. Sternum length 1.99, width 2.35. Abdomen length 4.23, width 3.52. Pedipalp: Fm 1.07, Pt 0.67, Ti 0.57, Ta 1.14, (total) 3.45. Leg I: Fm 3.60, Pt 2.01, Ti 3.56, Mt 2.99, Ta 1.26, (total) 13.42. Leg II: Fm 4.73, Pt 1.73, Ti 4.10, Mt 3.65, Ta 1.43, (total) 15.64. Leg III: Fm 5.10, Pt 1.59, Ti 4.25, Mt 3.48, Ta 1.35, (total) 15.77. Leg IV: Fm 5.14, Pt 1.26, Ti 4.14, Mt 3.25, Ta 1.44, (total) 15.23.

*Male (paratype):*Color: carapace yellow-brown, with slightly darker marks laterally and medially; sternum pale yellow-brown; chelicerae red-brown with darker infuscations anteromedially to laterally; labium pale yellow-brown; abdomen dorsally yellow-brown with darker markings; ventrally pale yellow-brown; legs pale yellow-brown with all segments clearly annulated, though not as clear distally, annulations not encircling legs entirely. Cephalothorax: setae of medium length and thickness; 0.78 times longer than broad; fovea longitudinal, broad, very shallow. Eyes:AER slightly recurved; PER recurved; PME same size as AME, PLE largest, ALE smallest; eye group width 1.32; eye diameters, AME 0.21, ALE 0.13, PME 0.21, PLE 0.27; interdistances AME-ALE 0.29, PME-PLE 0.25, ALE-PLE 0.27, AME-PME 0.04; ocular quadrangle AME-AME 0.17, PME-PME 0.57; clypeus 0.04 high. Mouthparts: chelicerae with a few stout setae medially and anteriorly; lateral boss present, smooth; promargin with 3 teeth, retromargin with 2 teeth; maxillae longer than broad, with tuft of conspicuous setae distally; labium distally rounded. Sternum:0.83 times longer than broad, posteriorly indented. Pedipalp: femur, spination dorsal 0–1–2; retrolateral tibial apophysis with 2 apophyses, dorsal apophysis large and wide, directed slightly laterally in ventral view, ventral apophysis slightly narrower and rounded at tip; cymbial scopulae absent, cymbium round, angled bottom right; conductor large with quadrangular apophysis, with one end elongated and directed distally; embolus long and slender, large base, tapers abruptly, beginning at 6 o’clock, terminating at 12 o’clock; MA very large with two processes, one more sclerotized than the other (Figs 49–50). Legs:leg I much shorter than III, slightly shorter than IV; leg formula 3412; scopulae absent on all legs; tarsus I–IV with strong claw tufts; pr claws with c.10–15 teeth, rl claws with 3 or 4 teeth; spination: leg I, Fm pr 1–1–0, d 1–1–1, rl 0; Ti d 0, v 2–2–2–2–2; Mt v 2–2–2–2; Ti and Mt I and II with strong spines; leg II, Fm pr 0, d 1–1–1, rl 0; Ti v 2–2–2–2–2; Mt v 2–2–2–2; leg III, Fm pr 0, d 1–1–1, rl 0; Ti 1–0–0; Mt 1–0; leg IV, Fm pr 0, d 1–1–1, rl 0; Ti v 1–1; Mt 0. Abdomen:terminal setal tufts present. *Dimensions:* Total length 5.36. Cephalothorax length 2.57, width 3.29. Sternum length 1.53, width 1.84. Abdomen length 2.79, width 2.67. Pedipalp: Fm 0.78, Pt 0.42, Ti 0.44, Ta 0.75, (total) 2.39. Leg I: Fm 3.40, Pt 1.32, Ti 3.23, Mt 2.74, Ta 1.37, (total) 12.06. Leg II: Fm 5.06, Pt 1.62, Ti 4.07, Mt 3.55, Ta 1.44, (total) 10.68. Leg III: Fm 5.38, Pt 1.40, Ti 4.26, Mt 3.33, Ta 1.51, (total) 15.88. Leg IV: Fm 4.26, Pt 1.05, Ti 3.52, Mt 3.31, Ta 1.35, (total) 13.49.

###### Natural history.

Found on a rock wall and under bark (Fig. 96).

###### Distribution.

The type locality only (Fig. 96; Map 7).

##### Karaops vadlaadambara sp. n.

urn:lsid:zoobank.org:act:4E75A903-27CF-4809-8724-0055055D19B3

http://species-id.net/wiki/Karaops_vadlaadambara

Figs 51–54 Map 9

###### Type material.

Holotype male (SAM N199353): Arcoona Creek, near Sambot Waterhole, Gammon Ranges National Park, 30°27'S, 139°02'E, South Australia, Australia, 4.V.1989, D. Hirst. Paratypes: same data as holotype, 1♀ (SAM N199354).

###### Other material examined.

**AUSTRALIA: South Australia:** same data as holotype, 1 immature (SAM N199355); Gammon Ranges National Park, West of Arcoona Bluff [30°25'S, 138°59'E], 3.V.1989, D. Hirst, 1♂ (SAM N199358); Gammon Ranges National Park, near Sambot Waterhole, Arcoona Creek, 30°27'S, 139°02'E, 4.V.1989, D.C. Lee, 2♀ (SAM N199351-2); Gammon Ranges National Park, near Sambot Waterhole, Arcoona Creek, 30°27'S, 139°02'E, 4.V.1989, D. Hirst, 2♀ (SAM N199360-1); Mount Serle Station, 0.4 km northwest Mount Cline, 30°20'54"S, 138°46'41"E, 16–27.XI.1998, Flinders Range survey, pitfalls, 1♂ (SAM NN20978); Wilpena Pound, Wilpena Creek, 31°30'S, 138°36'E, 24.IV.1987, D. Hirst, 1♀, 1 immature (SAM N199349).

###### Etymology.

The specific name comes from the Adnyamathanha words *vadla*, meaning flat, and *adambara*, meaning spider, in the language of the indigenous Adnyamathanha people of the region of the type locality. The name is to be treated as a noun in apposition.

###### Diagnosis.

**Figures 51–58.**
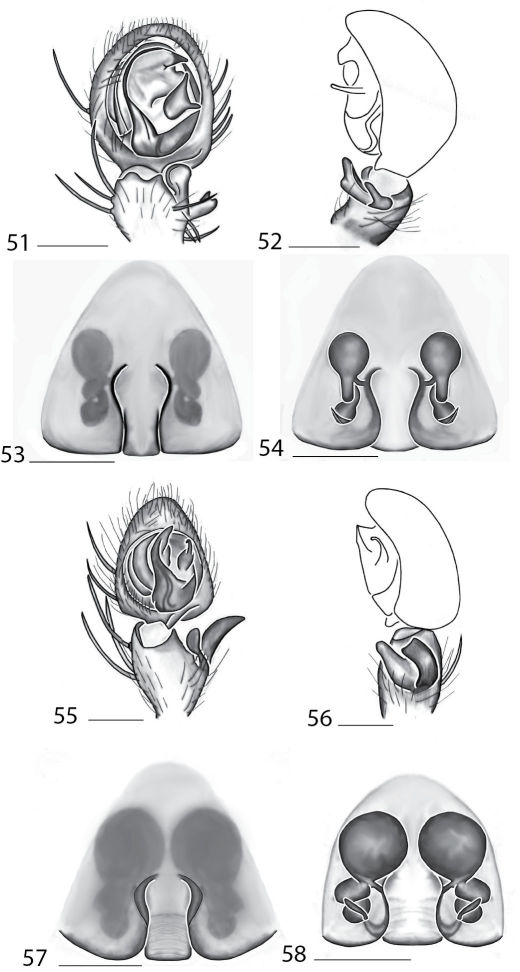
Copulatory organs of *Karaops vadlaadambara* sp. n., male holotype from Arcoona Creek, near Sambot Waterhole, Gammon Ranges National Park, South Australia, Australia (SAM N199353) (**51–52**), female paratype from Arcoona Creek, near Sambot Waterhole, Gammon Ranges National Park, South Australia, Australia (SAM N199354) (**53–54**), and *Karaops pilkingtoni* sp. n., male holotype from Trig Hill, Old Telegraph Station, Alice Springs, Northern Territory, Australia (WAM T76590) (**55–56**), female paratype from Alice Springs, Northern Territory, Australia (SAM N199359) (**57–58**), **51, 55 **male pedipalp, ventral view **52, 56** male pedipalp, retrolateral view **53, 57** epigyne, ventral view **54, 58** spermathecae, dorsal view. Scale bar: 0.25 mm.

Males can be differentiated from all other species by having a portion of the conductor behind the MA, and the conductor being sclerotized terminally. Additionally, the embolus is short and hook shaped, and in the center of the bulb rather than the lateral edge (Fig. 51). Females can be separated from other species by a quadrangular medium septum and well-separated, round spermathecae and short copulatory ducts (Figs 53–54).

###### Description.

*Male (holotype):*Color: carapace yellow-brown, with slightly darker marks medially; sternum pale yellow-brown; chelicerae pale yellow with darker infuscations anteriorly; maxillae pale yellow-brown; labium pale yellow-brown; abdomen dorsally yellow-brown with red-brown markings; ventrally pale yellow-brown; legs with femora, patellae and tibiae I-IV clearly annulated, yellow-brown, darkening distally; annulations not entirely encircling legs. Cephalothorax:setae short, stout and rodlike; 0.88 times longer than broad; fovea longitudinal, broad, very shallow. Eyes:AER slightly recurved; PER recurved; PME larger than AME, PLE largest, ALE smallest; eye group width 1.25; eye diameters, AME 0.15, ALE 0.09, PME 0.18, PLE 0.27; interdistances AME-ALE 0.23, PME-PLE 0.20, ALE-PLE 0.16, AME-PME 0.04; ocular quadrangle AME-AME 0.38, PME-PME 0.69; clypeus 0.12 high. Mouthparts: chelicerae with a few stout setae medially and anteriorly; lateral boss present, smooth; promargin with 3 teeth, retromargin with 2 teeth; maxillae longer than broad, with tuft of conspicuous setae distally; labium distally rounded. Sternum:0.87 times longer than broad, posteriorly indented. Pedipalp:femur, spination dorsal 0–1–1; retrolateral tibial apophysis with 2 processes, dorsal process small and directed laterally, ventral process slightly larger, dilated at tip; retrolateral basal cymbial process present; cymbial scopulae absent, cymbium oval and angled bottom right. Conductor large, pointed at tip, tip well-sclerotized; embolus very long and slender, wide at base and tapering, to abruptly tapering midway, hook-like, going up center of palpal bulb, beginning at 6 o’clock, terminating at 1 o’clock; MA large with two processes (Figs 51–52). Legs:leg I much shorter than legs II, III and IV; leg formula 3421; scopulae absent on all legs; tarsus I–IV with strong claw tufts; claws without teeth; spination: leg I, Fm pr 1–1–0, d 1–1–1, rl 0; Ti d 0, v 2–2–2–2–2; Mt v 2–2–2; Ti and Mt I and II with strong spines; leg II, Fm pr 0, d 1–1–1, rl 0–1–1; Ti v 2–2–2–2–2; Mt v 2–2–2; leg III, Fm pr 0, d 1–1–1, rl 0–1–1; Ti 0; Mt 0; leg IV, Fm pr 0, d 1–1–1, rl 0; Ti 0; Mt 0. Abdomen:terminal setal tufts present. *Dimensions:* Total length 4.68. Cephalothorax length 2.34, width 2.65. Sternum length 1.25, width 1.44. Abdomen length 2.40, width 2.19. Pedipalp: Fm 0.70, Pt 0.45, Ti 0.41, Ta 0.75, (total) 2.31. Leg I: Fm 3.12, Pt 1.19, Ti 2.75, Mt 2.34, Ta 1.16, (total) 10.56. Leg II: Fm 4.00, Pt 1.29, Ti 3.24, Mt 2.84, Ta 1.31, (total) 12.68. Leg III: Fm 4.37, Pt 1.17, Ti 3.48, Mt 3.07, Ta 1.27, (total) 13.36. Leg IV: Fm 4.20, Pt 1.06, Ti 3.32, Mt 3.04, Ta 1.28, (total) 12.90.

*Female (paratype):*Color: carapace yellow-brown, with slightly darker marks medially; sternum pale yellow-brown; chelicerae pale yellow with darker infuscations anteriorly; maxillae pale yellow-brown; labium pale yellow-brown; abdomen dorsally yellow-brown with red-brown markings; ventrally pale yellow-brown; legs with segments clearly annulated, but annulations do not completely encircle femorae, legs darkening distally at tibiae; annulations lighter in centers giving a ‘leopard spot’ appearance. Cephalothorax:setae short, stout, rodlike; 0.89 times longer than broad; fovea longitudinal, broad, very shallow. Eyes:AER slightly recurved; PER recurved; PME larger than AME, PLE largest, ALE smallest; eye group width 1.56; eye diameters, AME 0.14, ALE 0.10, PME 0.20, PLE 0.28; interdistances AME-ALE 0.30, PME-PLE 0.28, ALE-PLE 0.19, AME-PME 0.06; ocular quadrangle AME-AME 0.46, PME-PME 0.85; clypeus 0.13 high. Mouthparts: chelicerae with a few stout setae medially and anteriorly; lateral boss present, smooth; promargin with 3 teeth, retromargin with 2 teeth; maxillae longer than broad, with tuft of conspicuous setae distally; labium distally rounded. Sternum: 0.88 times longer than broad, posteriorly indented. Pedipalp:tarsus slightly swollen, claw present with c. 6 teeth. Legs:leg I only slightly shorter than legs II, III and IV; leg formula 3241; scopulae absent on all legs; tarsus I–IV with strong claw tufts; claws without teeth; spination: leg I, Fm pr 1–1–0, d 1–1–1, rl 0; Ti d 0, v 2–2–2–2–2; Mt v 2–2–2; Ti and Mt I and II with strong spines; leg II, Fm pr 0, d 1–1–1, rl 0–1–1; Ti v 2–2–2–2–2; Mt v 2–2–2; leg III, Fm pr 0, d 1–1–1, rl 0–1–1; Ti 0; Mt 0; leg IV, Fm pr 0, d 1–1–1, rl 0; Ti 0; Mt 0. Abdomen:possible setal tufts (hairs worn off). Epigyne:lateral lobes surrounding a quadrate to keyhole shaped median area, copulatory openings located anterolaterally, epigynal pockets absent; internally, short copulatory ducts lead to round spermathecae, fertilization ducts located posteriorly, posterodorsal fold absent (Figs 53–54). *Dimensions:* Total length 5.51. Cephalothorax length 2.92, width 3.28. Sternum length 1.47, width 1.68. Abdomen length 2.86, width 2.77. Pedipalp: Fm 0.92, Pt 0.51, Ti 0.66, Ta 1.18, (total) 3.27. Leg I: Fm 2.86, Pt 1.36, Ti 2.54, Mt 1.39, Ta 1.00, (total) 9.15. Leg II: Fm 4.08, Pt 1.45, Ti 2.86, Mt 2.47, Ta 1.09, (total) 11.95. Leg III: Fm 4.20, Pt 1.37, Ti 3.07, Mt 2.68, Ta 1.10, (total) 12.42. Leg IV: Fm 3.96, Pt 1.19, Ti 3.02, Mt 2.67, Ta 1.07, (total) 11.91.

###### Natural history.

Collected at night.

###### Distribution.

**Map 9.**
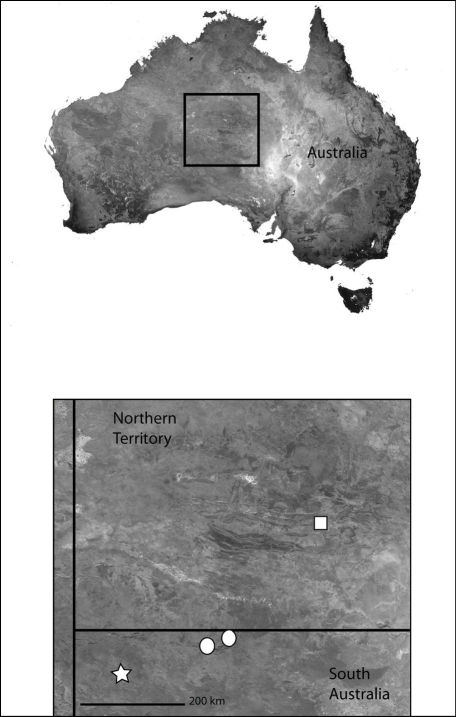
Central Australia (inset) showing the distribution of *Karaops* gen. n. *Karaops pilkingtoni* sp. n.<br/> (white square), *Karaops ngarutjaranya* sp. n. (white circles), *Karaops deserticola* sp. n. (white star).

Known from throughout the Gammon and Flinders Ranges in South Australia (Map 9).

**Map 10.**
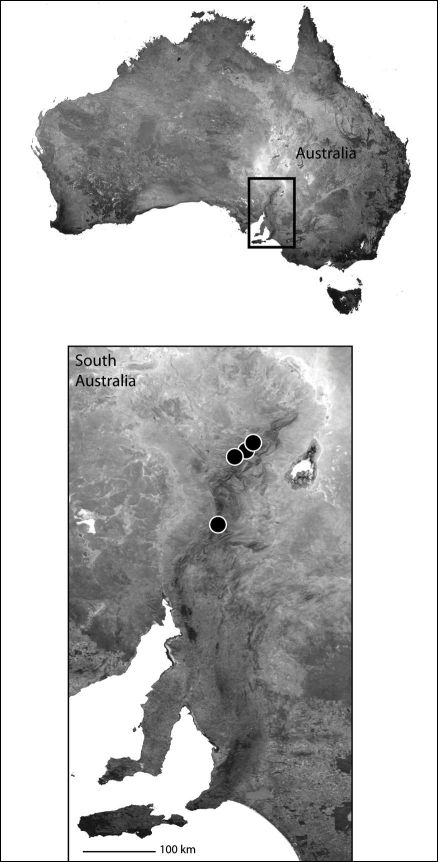
Southern central Australia (inset) showing the distribution of *Karaops vadlaadambara* sp. n. (black circles).

##### Karaops pilkingtoni sp. n.

urn:lsid:zoobank.org:act:D2AD07A0-FFBE-42AB-85C1-1FAC6A4D36B8

http://species-id.net/wiki/Karaops_pilkingtoni

Figs 55–58 Map 9

###### Type material.

Holotype male (WAM T76590): base of Trig Hill, Old Telegraph Station, Alice Springs, Northern Territory, Australia, 23°40'S, 134°14'E, 3.V.1986, B.J. Scott, under rocks. Paratype. Alice Springs, Grid reference 2741.1000, 1:250,000 sheet [23°16'15"S, 134°52'50"E], 25.VI.1978, F. and J. Aslin, 1♀ (SAM N199359).

###### Etymology.

This species is named in honor of Karl Pilkington.

###### Diagnosis.

The male has a thick and short embolus that bisects the bulb (Fig. 55). The female has a wrinkled median septum and huge, round spermathecae that almost touch medially (Figs 57–58).

###### Description.

*Male (holotype):*Color: carapace uniformly yellow-brown; sternum pale yellow-brown; chelicerae pale yellow with darker infuscations anteriorly; maxillae pale yellow-brown; labium pale brown; abdomen dorsally yellow-brown with red-brown markings; ventrally pale yellow-brown; legs with all segments clearly annulated. Cephalothorax:setae short, stout, rodlike; 0.92 times longer than broad; fovea longitudinal, broad, very shallow. Eyes:AER nearly straight; PER recurved; PME larger than AME, PLE largest, ALE smallest; eye group width 1.21; eye diameters, AME 0.16, ALE 0.07, PME 0.20, PLE 0.23; interdistances AME-ALE 0.19, PME-PLE 0.17, ALE-PLE 0.25, AME-PME 0.03; ocular quadrangle AME-AME 0.42, PME-PME 0.66; clypeus 0.09 high. Mouthparts:chelicerae with a few stout setae medially and anteriorly; lateral boss present, smooth; promargin with 3 teeth, retromargin with 2 teeth; maxillae longer than broad, with tuft of conspicuous setae distally; labium distally rounded. Sternum:0.81 times longer than broad, posteriorly indented. Pedipalp:femur, spination dorsal 0–1–1; retrolateral tibial apophysis with 2 processes, dorsal apophysis directed laterally, blade like in ventral view, ventral apophysis flattened at tip; retrolateral basal cymbial process present; cymbial scopulae absent, cymbium triangular, conductor pointed, blade like; embolus short and stout, beginning at 6 o’clock, directed distally through center of bulb, toward 12 o’clock; MA long, with a wide base that tapers to a small hook, directed distally (Figs 55–56). Legs:leg I only slightly shorter than legs II, III and IV; leg formula unknown (at least one leg missing); scopulae absent on all legs; tarsi with strong claw tufts; claws without teeth; spination: leg I, Fm pr 1–1–0, d 1–1–1, rl 0; Ti d 0, v 2–2–2–2–2; Mt v 2–2–2; Ti and Mt I and II with strong spines; leg II, Fm pr 0, d 1–1–1, rl 0–1–1; Ti v 2–2–2–2–2; Mt v 2–2–2; leg III, Fm pr 0, d 1–1–1, rl 0–1–1; Ti 0; Mt 0; leg IV, F pr 0, d 1–1–1, rl 0; Ti 0; Mt 0. Abdomen:terminal setal tufts present. *Dimensions:* Total length 3.89. Cephalothorax length 2.31, width 2.51. Sternum length 1.13, width 1.39. Abdomen length 1.59, width 1.79. Pedipalp: Fm 0.65, Pt 0.55, Ti 0.29, Ta 0.65, (total) 2.14. Leg II: Missing. Leg III: Fm 4.52, Pt 1.22, Ti 3.88, Mt 3.36, Ta 1.38, (total) 14.36. Leg IV: Fm 4.32, Pt 1.15, Ti 3.51, Mt 3.27, Ta 1.55, (total) 13.80.

*Female (paratype):*Color: carapace yellow-brown, with slightly darker marks medially; sternum pale yellow-brown; chelicerae pale yellow with darker infuscations anteriorly and laterally; maxillae pale yellow-brown; labium pale brown; abdomen dorsally yellow-brown with red-brown markings; ventrally pale yellow-brown; legs with all segments clearly annulated. Cephalothorax:setae short stout and rodlike; 0.83 times longer than broad; fovea longitudinal, broad, very shallow. Eyes:AER nearly straight; PER slightly recurved; PME larger than AME, PLE largest, ALE smallest; eye group width 1.62; eye diameters, AME 0.20, ALE 0.12, PME 0.25, PLE 0.28; interdistances AME-ALE 0.32, PME-PLE 0.30 ALE-PLE 0.29, AME-PME 0.07; ocular quadrangle AME-AME 0.50, PME-PME 0.92; clypeus 0.16 high. Mouthparts:chelicerae with a few stout setae medially and anteriorly; lateral boss present, smooth; promargin with 3 teeth, retromargin with 2 teeth; maxillae longer than broad, with tuft of conspicuous setae distally; labium distally rounded. Sternum:0.78 times longer than broad, posteriorly indented. Pedipalp:tarsus slightly swollen, claw present, without teeth. Legs:leg I only slightly shorter than legs II, III and IV; leg formula 3241; scopulae absent on all legs; tarsus I–IV with strong claw tufts; claws without teeth; spination: leg I, Fm pr 1–1–0, d 1–1–1, rl 0; Ti d 0, v 2–2–2–2–2; Mt v 2–2–2; Ti and Mt I and II with strong spines; leg II, Fm pr 0, d 1–1–1, rl 0; Ti v 2–2–2–2–2; Mt v 2–2–2–2; leg III, Fm pr 0, d 1–1–1, rl 0; Ti 0; Mt 0; leg IV, Fm pr 0, d 1–1–1, rl 0; Ti 0; Mt 0. Abdomen:possible setal tufts, hairs worn off. Epigyne:lateral lobes separated by slightly wrinkled, unsclerotized, quadrate fleshy median area, copulatory openings located anterolaterally, epigynal pockets absent; internally, small ducts lead to extremely large round spermathecae, fertilization ducts located posteriorly, posterodorsal fold absent (Figs 57–58). *Dimensions:* Total length 5.25. Cephalothorax length 2.68, width 3.22. Sternum length 1.35, width 1.72. Abdomen length 3.02, width 2.81. Pedipalp: Fm 0.73, Pt 0.60, Ti 0.67, Ta 0.81, (total) 2.81. Leg I: Fm 3.12, Pt 1.35, Ti 2.80, Mt 2.24, Ta 1.08, (total) 10.59. Leg II: Fm 3.96, Pt 1.46, Ti 3.30, Mt 2.65, Ta 1.25, (total) 12.62. Leg III: Fm 4.84, Pt 1.40, Ti 3.54, Mt 2.90, Ta 1.31, (total) 13.99. Leg IV: Fm 4.06, Pt 1.36, Ti 3.12, Mt 2.72, Ta 1.24, (total) 12.50.

###### Natural history.

Collected from under rocks.

###### Distribution.

Only from Alice Springs (Map 9).

##### Karaops deserticola sp. n.

urn:lsid:zoobank.org:act:F487CD06-97AF-444A-BA50-6E42977B6BB3

http://species-id.net/wiki/Karaops_deserticola

Figs 59–60 Map 9

###### Type material.

Holotype female (SAM N199350): Mount Lindsay, South Australia, Australia [27°02'S, 129°53'E], 28.VIII.1980, A. Lees, under rock slab on bare granite slope.

###### Etymology.

The specific epithet comes from the Latin word *desertum* which is a waste place or a wilderness and is an adjective that denotes the presence of this species in desert biotopes.

###### Diagnosis.

**Figures 59–64.**
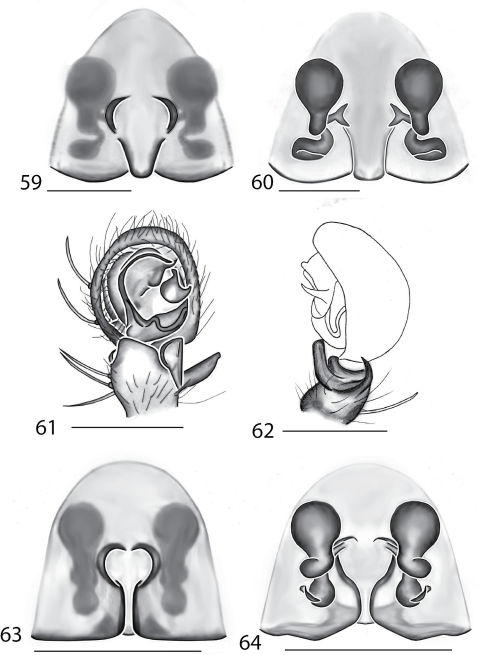
Copulatory organs of *Karaops deserticola* sp. n., holotype female from Mount Lindsay, South Australia, Australia (SAM N199350) (**59–60**), and *Karaops ngarutjaranya* sp. n., male holotype from northeast of Mount Woodroffe, South Australia, Australia (SAM NN10914) (**61–62**), female paratype from southeast of Womikata Bore Homeland, South Australia, Australia (SAM NN10915) (**63–64**), **59, 63** epigyne, ventral view **60, 64** spermathecae, dorsal view. **61** male pedipalp, ventral view **62** male pedipalp, retrolateral view.Scale bar: 0.25 mm (59–60), 0.50 mm (61–64).

Females can be differentiated from other species by the median septum of the copulatory organs tapering posteriorly, giving it a subtriangular appearance (Fig. 59). Males unknown.

###### Description.

*Holotype:*Color: carapace yellow-brown, with slightly darker marks medially; sternum pale yellow-brown; chelicerae pale yellow with darker infuscations anteriorly; maxillae pale yellow-brown; labium pale yellow-brown; abdomen dorsally yellow-brown with red-brown and grey markings; ventrally pale yellow-brown; legs with segments clearly annulated, but annulations do not completely encircle femorae, legs darkening distally at tibiae; annulations lighter in centers giving a ‘leopard spot’ appearance. Cephalothorax:setae short, stout, rodlike; 0.85 times longer than broad; fovea longitudinal, broad, very shallow. Eyes:AER nearly straight; PER recurved; PME larger than AME, PLE largest, ALE smallest; eye group width 1.64; eye diameters, AME 0.17, ALE 0.12, PME 0.23, PLE 0.34; interdistances AME-ALE 0.30, PME-PLE 0.27, ALE-PLE 0.18, AME-PME 0.06; ocular quadrangle AME-AME 0.48, PME-PME 0.92; clypeus 0.15 high. Mouthparts:chelicerae with a few stout setae medially and anteriorly; lateral boss present, smooth; promargin with 3 teeth, retromargin with 2 teeth; maxillae longer than broad, with tuft of conspicuous setae distally; labium distally rounded. Sternum:0.84 times longer than broad, posteriorly indented. Pedipalp:tarsus slightly swollen, claw present, without teeth. Legs:leg I only slightly shorter than legs II, III; leg III longest; scopulae absent on all legs; tarsus I–IV with strong claw tufts; claws prolateral claw with 1 or 2 small teeth; spination: leg I, Fm pr 1–1–0, d 1–1–1, rl 0; Ti d 0, v 2–2–2–2–2; Mt v 2–2–2; Ti and Mt I and II with strong spines; leg II, Fm pr 0, d 1–1–1, rl 0; Ti v 2–2–2–2–2; Mt v 2–2–2; leg III, Fm pr 0, d 1–1–1, rl 0; Ti 0; Mt 0; leg IV, Fm pr 0, d 1–1–1, rl 0; Ti 0; Mt 0. Abdomen:possible setal tufts, hairs worn off. Epigyne:lateral lobes surround triangular median area, two parentheses-like marks located in the center of the plate, strongly sclerotized, copulatory openings located under these, epigynal pockets absent; internally, short ducts lead to round, well separated spermathecae, copulatory ducts twist posteriorly to fertilization ducts, posterodorsal fold absent (Figs 59–60). *Dimensions:* Total length 5.72. Cephalothorax length 2.91, width 3.44. Sternum length 1.51, width 1.79. Abdomen length 2.91, width 2.75. Pedipalp: Fm 0.77, Pt 0.57, Ti 0.48, Ta 0.77, (total) 2.59. Leg I: Fm 3.37, Pt 1.46, Ti 2.96, Mt 1.81, Ta 1.14, (total) 10.74. Leg II: Fm 3.88, Pt 1.48, Ti 2.96, Mt 2.77, Ta 1.18, (total) 12.27. Leg III: F 4.44, Pt 1.63, Ti 3.51, Mt 3.04, tarsus 1.17, (total) 13.79. Leg IV: Missing.

###### Natural history.

Collected from under rocks.

###### Distribution.

The type locality only (Map 9).

##### Karaops ngarutjaranya sp. n.

urn:lsid:zoobank.org:act:D59EAE45-737B-49FF-9B7C-38B9C14429DF

http://species-id.net/wiki/Karaops_ngarutjaranya

Figs 61–64 Map 9

###### Type material.

Holotype male (SAM NN10914): 8 km northeast of Mount Woodroffe, South Australia, Australia, in gorge, 26°17'S, 131°48'E, 15.X.1994, D. Hirst. Paratype: 2.5 km southwest of Womikata Bore Homeland, South Australia, Australia, 26°07'S, 132°08'E, 20.X.1994, D. Hirst, 1♀ (SAM NN10915).

###### Etymology.

The specific epithet refers to the indigenous Pitjantjatjara name for Mount Woodroofe, the type locality. The name is to be treated as a noun in apposition.

###### Diagnosis.

Males of this species can be differentiated from others by having a sinuate anterior margin of the conductor, having two branches on the MA, and a hook-shaped embolus that bisects the palpal bulb (Fig. 61). The females can also be separated from other species by having a keyhole-shaped median septum, and lateral lobes nearly touching posteriorly (Fig. 63).

###### Remarks.

Although the male and female specimens were not collected together (they were collected some 46 km apart), it seems reasonable to assume they are conspecific.

###### Description.

*Male (holotype):*Color: carapace uniformly yellow-brown; sternum pale yellow-brown; chelicerae pale yellow with darker infuscations anteriorly; maxillae pale yellow-brown; labium pale brown; abdomen dorsally yellow-brown with red-brown and grey markings; ventrally pale yellow-brown; legs with all segments clearly annulated. Cephalothorax:setae short, stout, rodlike; 0.9 times longer than broad; fovea longitudinal, broad, very shallow. Eyes:AER slightly recurved; PER recurved; PME larger than AME, PLE largest, ALE smallest; eye group width 1.2; eye diameters, AME 0.15, ALE 0.09, PME 0.18, PLE 0.24; interdistances AME-ALE 0.21, PME-PLE 0.21, ALE-PLE 0.18, AME-PME 0.03; ocular quadrangle AME-AME 0.38, PME-PME 0.66; clypeus 0.07 high. Mouthparts:chelicerae with a few stout setae medially and anteriorly; lateral boss present, smooth; promargin with 3 teeth, retromargin with 2 teeth; maxillae longer than broad, with tuft of conspicuous setae distally; labium distally rounded. Sternum: 0.79 times longer than broad, posteriorly indented. Pedipalp: femur, spination dorsal 0–1–1; retrolateral tibial apophysis with 2 processes, dorsal process directed laterally, pointed at tip, blade like in ventral view, ventral process larger, flattened and subquadrangular at tip; retrolateral basal cymbial process present; cymbial scopulae absent, cymbium oval. Conductor large, sinuous along anterior margin, tip pointed, curved distally; embolus long and slender, arising from oblong base, tapering, hooked beginning at 6 o’clock, terminating at 2 o’clock; MA with quadrate to oval base with two processes, one directed laterally, one distally (Figs 61–62). Legs:leg I much shorter than legs II, III and IV; leg formula 3241; scopulae absent on all legs; tarsus I–IV with strong claw tufts; pr claws with 1 or 2 small teeth; spination: leg I, Fm pr 1–1–0, d 1–1–1, rl 0; Ti d 0, v 2–2–2–2–2; Mt v 2–2–2; Ti and Mt I and II with weak spines; leg II, Fm pr 0, d 1–1–1, rl 0; Ti v 2–2–2–2–2; Mt v 2–2–2; leg III, Fm pr 0, d 1–1–1, rl 0; Ti 0; Mt 0; leg IV, Fm pr 0, d 1–1–1, rl 0; Ti 0; Mt 0. Abdomen:without terminal setal tufts. *Dimensions:* Total length 3.99. Cephalothorax length 2.20, width 2.45. Sternum length 1.10, width 1.40. Abdomen length 2.03, width 2.03. Pedipalp: Fm 0.66, Pt 0.42, Ti 0.36, Ta 0.62, (total) 2.06. Leg I: Fm 3.21, Pt 1.15, Ti 2.87, Mt 2.44, Ta 1.25, (total) 10.92. Leg II: Fm 4.21, Pt 1.25, Ti 3.55, Mt 3.01, Ta 1.38, (total) 13.40. Leg III: Fm 4.60, Pt 1.25, Ti 3.76, Mt 3.14, Ta 1.44, (total) 14.19. Leg IV: Fm 4.26, Pt 1.10, Ti 3.43, Mt 3.12, Ta 1.41, (total) 13.32.

*Female (paratype):*Color: carapace yellow-brown, with slightly darker marks medially; sternum pale yellow-brown; chelicerae pale yellow with darker infuscations anteriorly; maxillae pale yellow-brown; labium pale brown; abdomen dorsally yellow-brown with red-brown and grey markings; ventrally pale yellow-brown; legs with all segments clearly annulated. Cephalothorax: setae short, stout, rodlike; 0.84 times longer than broad; fovea longitudinal, broad, very shallow. Eyes:AER slightly recurved; PER recurved; PME larger than AME, PLE largest, ALE smallest; eye group width 1.64; eye diameters, AME 0.18, ALE 0.14, PME 0.22, PLE 0.31; interdistances AME-ALE 0.31, PME-PLE 0.30, ALE-PLE 0.21, AME-PME 0.05; ocular quadrangle AME-AME 0.49, PME-PME 0.90; clypeus 0.1 high. Mouthparts:chelicerae with a few stout setae medially and anteriorly; lateral boss present, smooth; promargin with 3 teeth, retromargin with 2 teeth; maxillae longer than broad, with tuft of conspicuous setae distally; labium distally rounded. Sternum:0.77 times longer than broad, posteriorly indented. Pedipalp:tarsus slightly swollen, claw present, with c. 6 teeth. Legs:leg I only slightly shorter than legs II, III and IV; leg formula 3421; scopulae absent on all legs; tarsus I–IV with strong claw tufts; claws without teeth; spination: leg I, Fm pr 1–1–0, d 1–1–1, rl 0; Ti d 0, v 2–2–2–2–2; Mt v 2–2–2; Ti and Mt I and II with strong spines; leg II, Fm pr 0, d 1–1–1, rl 0; Ti v 2–2–2–2–2; Mt v 2–2–2; leg III, Fm pr 0, d 1–1–1, rl 0; Ti 0; Mt 0; leg IV, Fm pr 0, d 1–1–1, rl 0; Ti 0; Mt 0. Abdomen:without tufts of setae. Epigyne:lateral lobes nearly touching posteriorly, separated by keyhole-shaped median area, widening medially, anterolateral edges strongly sclerotized, copulatory openings located under these, epigynal pockets absent; internally small ducts connect to round, well-separated spermathecae, fertilization ducts located posteriorly, posterodorsal fold absent (Figs 63–64). *Dimensions:* Total length 7.14. Cephalothorax length 2.81, width 3.33. Sternum length 1.40, width 1.83. Abdomen length 4.37, width 4.11. Pedipalp: Fm 0.99, Pt 0.56, Ti 0.58, Ta 0.90, (total) 3.03. Leg I: Fm 3.28, Pt 1.44, Ti 2.86, Mt 2.27, Ta 1.14, (total) 10.99. Leg II: Fm 4.20, Pt 1.54, Ti 3.17, Mt 2.70, ta 1.25, (total) 12.86. Leg III: Fm 4.60, Pt 1.56, Ti 3.64, Mt 3.02, Ta 1.32, (total) 14.14. Leg IV: Fm 4.29, Pt 1.33, Ti 3.28, Mt 2.88, Ta 1.23, (total) 13.01.

###### Natural history.

No data

###### Distribution.

The type locality only (Map 9).

##### Karaops francesae sp. n.

urn:lsid:zoobank.org:act:9316B855-FBD8-4DB9-BA73-E783D479F2DD

http://species-id.net/wiki/Karaops_francesae

**Figures 65–72.**
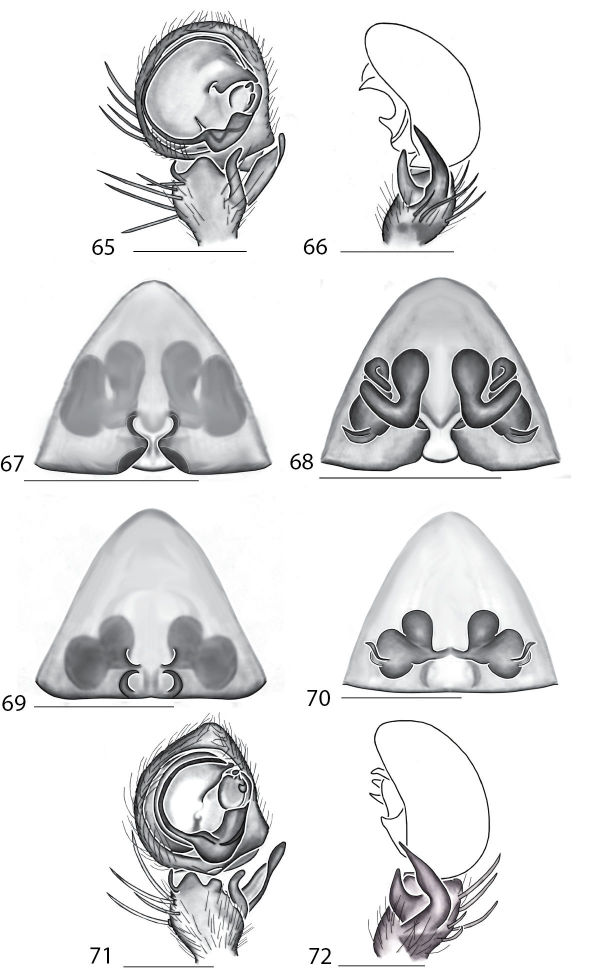
Copulatory organs of *Karaops francesae* sp. n., male holotype from northeast of slope of West Mount Barren, Fitzgerald River National Park, Western Australia, Australia (WAM T54996) (**65–66**), female paratype from east Mount Barren, Fitzgerald River National Park, Western Australia, Australia (WAM T54994) (**67–68**) and *Karaops toolbrunup* sp. n., female holotype from Toolbrunup, Stirling Ranges National Park, Western Australia, Australia (WAM T76592) (**69–70**), male paratype from Toolbrunup, Stirling Ranges National Park, Western Australia, Australia (WAM T62231) (**71–72**), **65, 71** male pedipalp, ventral view **66, 72** male pedipalp, retrolateral view **67, 69** epigyne, ventral view **68, 70** spermathecae, dorsal view. Scale bar: 0.50 mm.

Figs 65–68 94 100 Map 6

###### Type material.

Holotype male (WAM T54996): Fitzgerald River National Park, northeast slope of West Mount Barren, 34°13'S, 119°26'E, Western Australia, Australia, 28.V.1994, M.S. Harvey, J.M. Waldock, under rock. Paratype: Fitzgerald River National Park, south slopes of East Mount Barren, 33°55'S, 120°01'E, Western Australia, Australia, 26.V.1994, M.S. Harvey, J.M. Waldock, G. Harold, N. Brown, under rocks, 1♀ (WAM T54994).

###### Other material examined.

**AUSTRALIA: Western Australia:** Boat Harbour, 34°30'42"S, 118°48'13"E, 9.III.2007, G. Burne, on trunk of *Eucalyptus platypus*, 1♀ (WAM T81157); Cape Arid National Park, Mount Arid, south side near summit, 33°57'45"S, 123°13'01"E, 5.VI.2007, M.L. Moir, M.C. Leng, under rocks, 1♀, 1♂, (WAM T80667), Cape Arid National Park, site 1, Mount Arid, 33°59'14.8"S, 123°13'22.1"E, 25.X.2008, J.M. Waldock, S.C. Crews, under granite rocks on granite slope, 3♀, 2♂, ~32 immatures (WAM T94036, T94046); Cape Le Grand National Park, outcrop above Rossiter Bay, site 4, 33°59'26"S, 122°15'34"E, 4.VI.2007, M.L Moir, M.C. Leng, under granite rocks, 1♀ (WAM T80725); Cape Le Grand, Lucky Bay, 34°00'S, 122°14'E, 19.V.1977, R.P. McMillan, 1♀ (WAM T93/1328); Cape Le Grand National Park, site 1, Rossiter Bay, 33°58'20.6"S, 122°16'13.0"E, 21.X.2008, J.M. Waldock, S.C. Crews, under granite on granite slope, 4♀, 1♂, ~33 immatures (WAM T93995, T94050, T94051, T94053, T94054, T94055, T97252); Cape Riche, Mount Melville, near summit, 34°35'46"S, 118°44'33"E, 8.VI.2007, M.L. Moir, M.C. Leng, under laterite rocks, 1♂ (WAM T80649); Duke of Orleans Bay, Mount Belches, southern side, site 3, 33°56'22"S, 122°34'50"E, 2.VI.2007, M.L. Moir, A. Longbottom, under granite rocks, 1♀, 1♂, 1 immature (WAM T80695); Fitzgerald River National Park, rocky outcrop northeast of Pt. Edwards, site 3, 33°57'37"S, 119°57'28"E, 26.V.2007, M.L. Moir, M.C. Leng, under rock, 1♀, 1♂, (WAM T80760); Fitzgerald River National Park, Northeast slope of West Mount Barren, 34°13'S, 119°26'E, 28.V.1994, M.S. Harvey, J.M. Waldock, under rocks, 1♀ (WAM T54995); Fitzgerald River National Park, East Mount Barren, site 6, 33°55'29"S, 120°01'07"E, 25.XI.2006, M.L. Moir, K.E.C. Brennan, under rock, 1♀ (WAM T78498); Fitzgerald River National Park, East Mount Barren, site 7, 33°55'28"S, 120°01'13"E, 25.XI.2006, M.L. Moir, K.E.C. Brennan, under rock, 2♀, 1♂ (WAM T78499, T78500, T78501); Fitzgerald River National Park, south slopes of East Mount Barren, 33°55'S, 120°01'E, 28.V.1994, M.S. Harvey, J.M. Waldock, G. Harold, N. Brown, under rocks, 2♀, 1♂ (WAM T54993); Fitzgerald River National Park, West Mount Barren, 34°12'39.5"S, 119°25'58.6"E, 21.IV.2009, M. Rix, 1 penultimate ♂ (WAM T97291); Mount Lindesay, 34°50'35"S, 117°18'22"E, 6.V.2005, M.S. Harvey, under granite rock, 1♀ (WAM T66488); Mount Lindesay, granite outcrop, site 4, 34°50'41"S, 117°17'54"E, 20.X.2006, M.L. Moir, J.M. Waldock, under granite rock, 1♀ (WAM T78491); Mount Lindesay National Park, Mt. Lindesay, north of Denmark, first major granite outcrop along trail, ~2 km in, 34°50'41.1"S, 117°17'18.3"E, 10.II.2009, S.C. Crews, under granite, 5♀, 1♂ (WAM T97245-T97250), Ravensthorpe Range South, site WAM 01, 33°37'09.06"S, 120°09'58.01"E, 15.V.2007, M.C. Leng, M.L. Moir, under mallee bark, 1♂ (WAM 80848); Ravensthorpe Range South, site WAM 10, 33°38'16.03"S, 120°10'46.01"E, 17.V.2007, M.C. Leng, M.L. Moir, under rock, 1♂ (WAM T80881); Ravensthorpe Range South, site WAM 41, 33°36'40.05"S, 120°09'06.00"E, 24.V.2007, M.C. Leng, M.L. Moir, under rock, 1♀ (WAM T80996); Ravensthorpe Range South, site WAM 47, 33°38'19.07"S, 120°11'16.06"E, 28.V.2007; M.C. Leng, M.L. Moir, under rock, 1 immature (WAM T81019); Ravensthorpe Range North, Overshot Hill, site WAM 25, 33°32'04.02"S, 120°00'46.03"E, 20.V.2007, M.C. Leng, M.L. Moir, under rock, 1♂, 3♀ (WAM T80928, T80925, T80926, T80927); Recherche Archipelago: Wilson Island, 34°07'S, 122°00'E, 12.V.1991, J. Dell, 1♀ (WAM T93/1327); Recherche Archipelago, Woody Island, 33°57'S, 121°59'E, 9.V.1999, A.F. Longbottom, under granite flake, 1♂ (WAM T54997); Stirling Range National Park, Talyuberlup Picnic Site, 34°24'56"S, 117°57'18"E, 25.IV.1996, M.S. Harvey, J.M. Waldock, B.Y. Main, under bark of *Eucalyptus wandoo*, 1♂ (WAM T54999); Stirling Range National Park, Cascades Trail below Bluff Knoll, 34°22.332'S; 118°14.721'E, 9.II.2009, S.C. Crews, M. Harvey, under granite of small outcrop in forest, 1♀, 4 immatures (WAM T97232, T97241-T97244); Two Peoples Bay Nature Reserve, granite outcrop, site 6, 34°59'18"S, 118°11'44"E, 14.X.2006, M.L. Moir, J.M. Waldock, under granite rock, 2♀, 3♂ (WAM T78482, T78484-T78487); Two Peoples Bay Nature Reserve, above Robinson’s Gully, 34°59'30"S, 118°12'01"E, 28.V.2004, M.S. Harvey, under granite rocks, 1♂ (WAM T62230); Two Peoples Bay Nature Reserve, site MG08, top end of R7 gully, 34°59'44"S, 118°11'40"E, 26.X.1995, S. Comer, pitfall, 1♀ (WAM T67614); Two Peoples Bay Nature Reserve, granite outcrop, site 10, 35°00'20"S, 118°11'10"E, 19.X.2006, M.L. Moir, J.M. Waldock, under granite rock, 2♀, 1♂ (WAM T78488, T78490, T78489); Waychinicup National Park, Mount Manypeaks, granite outcrop, site 1, 34°53'30"S, 118°18'44"E, 26.X.2006, M.L. Moir, A. Sampey, under granite rock, 2♀ (WAM T78494, T78495); Waychinicup National Park, Mount Manypeaks, granite outcrop, site 2, 34°53'45"S, 118°18'07"E, 26.X.2006, M.L. Moir, A. Sampey, under granite rock, 1♀ (WAM T78492)

###### Etymology.

This species is named in honor of the second author’s (MSH) daughter, Frances Harvey.

###### Diagnosis.

**Males of this species** can be separated from others, in particular *Karaops toolbrunup* sp. n., by having the tip of the cymbium rounded and the embolus curving around the outer edge of the cymbium (Fig. 65). Females can be separated from other species by having epigynal pockets and the internal ducts (Figs 67–68).

###### Remarks.

Throughout its range, this species is subject to variation in body size, ventral tibial and metatarsal spination of legs I and II, as well as in the number of promarginal teeth. There are 6 pairs of spines ventrally on tibiae I and II in most specimens, but some specimens have 5 pairs. Typically, there are 4 pairs of spines located ventrally on the metatarsus, however, at least one specimen has a 2–2-1 pattern. This species has either 4 or 5 promarginal teeth. The leg lengths also vary a great deal and have been found to be 3421, 23=41, 3241 and 2341. There is no genitalic variation.

###### Description.

*Male (holotype):*Color: carapace yellow-brown, with slightly darker marks laterally and medially; sternum pale yellow-brown; chelicerae yellow-brown; maxillae pale yellow-brown; labium pale yellow-brown; abdomen dorsally pale creamy-yellow with a few darker flecks; ventrally pale yellow-brown; legs with femora, tibiae and tip of tarsi lightly annulated. Cephalothorax: setae long and thin; 0.94 times longer than broad; fovea longitudinal, broad, very shallow. Eyes:AER slightly recurved; PER slightly recurved; PME larger than AME, PLE largest, ALE smallest; eye group width 1.19; eye diameters, AME 0.17, ALE 0.08, PME 0.19, PLE 0.25; interdistances AME-ALE 0.29, PME-PLE 0.23, ALE-PLE 0.29, AME-PME 0.04; ocular quadrangle AME-AME 0.11, PME-PME 0.52; clypeus 0.08 high. Mouthparts:chelicerae with a few stout setae medially and anteriorly; lateral boss present, smooth; promargin with 4 teeth, retromargin with 3 teeth; maxillae longer than broad, with tuft of conspicuous setae distally; labium distally rounded. Sternum:0.95 times longer than broad, posteriorly indented. Pedipalp:femur, spination dorsal 0–1–2; retrolateral tibial apophysis with 2 processes, dorsal apophysis long, slightly sinusoidal, tapered and pointed in lateral view, ventral apophysis slightly shorter, pointed in lateral view; retrolateral basal cymbial process absent; cymbial scopulae absent, cymbium round to triangular, angled bottom right; conductor large pointed at tip, directed laterally; embolus long and slender, beginning at 6 o’clock, ending at 2 o’clock; MA short, ovoid, centrally depressed with two processes, one small and unsclerotized, the other a sclerotized, blunt hook (Figs 65–66). Legs:leg I only slightly shorter than legs II, III and IV; leg formula 3241; scopulae absent on all legs; tarsus I–IV with strong claw tufts; pr claws with c. 10 teeth, rl claws with none; spination: leg I, Fm pr 1–1–0, d 1–1–1, rl 0; Ti d 0, v 2–2–2–2–2–2; Mt v 2–2–2–2; Ti and Mt I and II with weak spines; leg II, Fm pr 1–1–1, d 1–1–1, rl 0–1–1; Ti v 2–2–2–2–2–2; Mt v 2–2–2–2; leg III, Fm pr 1–1–1, d 1–1–1, rl 0–1–1; Ti 0; Mt 0; leg IV, Fm pr 1–1–0, d 1–1–1, rl 1–1–1; Ti 0; Mt 0. Abdomen:terminal setal tufts present. *Dimensions:* Total length 5.15. Cephalothorax length 2.34, width 2.71. Sternum length 1.42, width 1.49. Abdomen length 2.81, width 2.03. Pedipalp: Fm 0.75, Pt 0.46, Ti 0.40, Ta 0.75, (total) 2.36. Leg I: Fm 3.89, Pt 1.03, Ti 3.18, Mt 2.96, Ta 1.24, (total) 12.20. Leg II: Fm 3.88, Pt 1.48, Ti 3.18, Mt 2.99, Ta 1.22, (total) 12.75. Leg III: Fm 4.22, Pt 1.18, Ti 3.22, Mt 2.96, Ta 1.37, (total) 12.95. Leg IV: Fm 4.07, Pt 0.96, Ti 3.22, Mt 2.82, Ta 1.17, (total) 12.24.

*Female* (paratype)*:*Color: carapace uniformly yellow-brown; sternum pale yellow; chelicerae pale yellow with darker infuscations anteriorly; maxillae pale yellow-brown; labium pale yellow-brown; abdomen dorsally yellow-brown with darker markings; ventrally pale yellow-brown; legs with femora and tibia lightly annulated, annulations don’t completely encircle legs, femora with lots of dark flecks. Cephalothorax:setae long and thin; 0.87 times longer than broad; fovea longitudinal, broad, very shallow. Eyes:AER nearly straight; PER slightly recurved; PME larger than AME, PLE largest, ALE smallest; eye group width 1.43; eye diameters, AME 0.15, ALE 0.06, PME 0.23, PLE 0.25; interdistances AME-ALE 0.42, PME-PLE 0.33, ALE-PLE 0.29, AME-PME 0.06; ocular quadrangle AME-AME 0.17, PME-PME 0.61; clypeus 0.1 high. Mouthparts:chelicerae with a few stout setae medially and anteriorly; lateral boss present, smooth; promargin with 5 teeth, retromargin with 3 teeth; maxillae longer than broad, with tuft of conspicuous setae distally; labium distally rounded. Sternum:0.93 times longer than broad, posteriorly indented. Pedipalp:tarsus slightly swollen, claw present, with c. 6 teeth. Legs:leg I only slightly shorter than legs II, III and IV; leg formula 3241; scopulae absent on all legs; pr claws with c. 10 teeth, rl claws with none; spination: leg I, Fm pr 1–1–0, d 1–1–1, rl 0; Ti d 0, v 2–2–2–2–2–2; Mt v 2–2–2–2; Ti and Mt I and II with strong spines; leg II, Fm pr 0, d 1–1–1, rl 0; Ti v 2–2–2–2–2–2; Mt v 2–2–2–2; leg III, Fm pr 0, d 1–1–1, rl 0; Ti 0; Mt 0; leg IV, Fm pr 0, d 1–1–1, rl 0; Ti 0; Mt 0. Abdomen:terminal setal tufts present. Epigyne:lateral lobes present, small comma-shaped marks, slightly sclerotized, in posterior third of plate, copulatory openings located here, epigynal pockets present; internally, ducts twisted 3 times leading to ovoid spermathecae, fertilization ducts located posteriorly, posterodorsal fold absent (Figs 67–68). *Dimensions:* Total length 5.87. Cephalothorax length 2.48, width 3.14. Sternum length 2.47, width 2.64. Abdomen length 3.38, width 2.85. Pedipalp: Fm 0.77, Pt 0.48, Ti 0.55, Ta 0.78, (total) 2.68. Leg I: Fm 2.92, Pt 1.27, Ti 2.70, Mt 2.18, Ta 1.13, (total) 10.20. Leg II: Fm 3.51, Pt 1.43, Ti 2.77, Mt 2.04, Ta 1.13, (total) 10.88. Leg III: Fm 3.07, Pt 1.18, Ti 2.93, Mt 2.40, Ta 1.08, (total) 11.29. Leg IV: Fm 3.26, Pt 1.09, Ti 2.52, Mt 2.28, Ta 1.18, (total) 10.33.

###### Natural history.

This species has been collected from under bark, on trees, and under rocks. The female guards her egg-sac (Fig. 94).

###### Distribution.

Found along the south coast of Western Australia from Mount Lindesay east to Mount Diamond, and in the islands of the Recherche Archipelago (Fig. 100; Map 6).

##### Karaops toolbrunup sp. n.

urn:lsid:zoobank.org:act:1600F5F8-2D04-445E-BA2A-F2CB72691FE9

http://species-id.net/wiki/Karaops_toolbrunup

Figs 69–72 95, 101 Map 6

###### Type material.

Holotype female (WAM T76592): Stirling Range National Park, Toolbrunup, 34°23'24"S, 118°03'14"E, scree slope, Western Australia, Australia, 5.IV.2004, M.S. Harvey, J.M. Waldock, K. Edward, C. Poustie, under rocks. Paratype: same data as for the holotype, 1♂ (WAM T62231).

###### Other material examined.

**AUSTRALIA: Western Australia:** Stirling Range National Park, Toolbrunup, 34°23'22.9"S, 118°03'12.8"E, 7.II.2009, S.C. Crews, under rocks on scree slope, 3♀, 1♂, 3 immatures (WAM T97234-T97240).

###### Etymology.

The specific epithet refers to the type locality, which means ‘drizzle carrier’ in the indigenous Nyoongar language. The name is to be treated as a noun in apposition.

###### Diagnosis.

This species can be separated from most others by having 4 teeth on the cheliceral promargin, and from *Karaops francesae* sp. n. by genitalic characteristics. In the male, the cymbium is pointed distally, and the base of the embolus extends to the basal margin of the cymbium (Fig. 71). In the female, epigynal pockets are present and the sperm ducts are not coiled (Figs 69–70).

###### Description.

*Female (holotype):*Color: carapace yellow-brown, with slightly darker marks laterally and medially; sternum pale yellow-brown; chelicerae pale yellow with darker infuscations anteriorly; maxillae pale yellow-brown, lightening distally; labium pale yellow-brown, lightening distally; abdomen dorsally pale creamy-yellow with a few darker flecks; ventrally pale yellow-brown; legs with segments clearly annulated, but annulations do not completely encircle femorae, legs darkening distally at tibiae; annulations lighter in centers giving a ‘leopard spot’ appearance. Cephalothorax: setae long and thin; 0.84 times longer than broad; fovea longitudinal, broad, very shallow. Eyes:AER nearly straight; PER slightly recurved; PME larger than AME, PLE largest, ALE smallest; eye group width 1.89; eye diameters, AME 0.19, ALE 0.13, PME 0.29, PLE 0.42; interdistances AME-ALE 0.52, PME-PLE 0.38, ALE-PLE 0.38, AME-PME 0.04; ocular quadrangle AME-AME 0.21, PME-PME 0.77; clypeus 0.06 high. Mouthparts:chelicerae with a few stout setae medially and anteriorly; lateral boss present, smooth; promargin with 4 teeth, retromargin with 3 teeth; maxillae longer than broad, with tuft of conspicuous setae distally; labium distally rounded. Sternum:0.89 times longer than broad, posteriorly indented. Pedipalp:tarsus slightly swollen, claw present with c. 6 teeth. Legs:leg I much shorter than legs II, III and IV; leg formula 3241; scopulae absent on all legs; tarsus I–IV with strong claw tufts; pr claws with c.10–15 teeth, rl claws with 1 or 2 teeth; spination: leg I, Fm L pr 2–1–0, d 1–1–1–1, rl 0 R pr 1–1–0, dorsal 1–1–1, rl 0; Ti d 0, v 2–2–2–2–2–2; Mt v 2–2–2–2; Ti and Mt I and II with strong spines; leg II, Fm pr 0, d 1–1–1, rl 0; Ti v 2–2–2–2–2–2; Mt v 2–2–2–2; leg III, Fm L pr 0, d 1–1–1–1, rl 0; R pr 0, d 1–1–1, rl 0; Ti 0; Mt 0; leg IV, Fm L pr 0, d 1–1–1, rl 0–0–1; R pr 0, d 1–1–1–1, rl 0; Ti v 1–1; Mt 1–0. Abdomen:terminal setal tufts present. Epigyne:lateral lobes indistinct, small comma-shaped sclerotizations in posterior third of plate where copulatory openings are located, epigynal pockets present; internally, ducts not coiled, small ducts lead to ovoid spermathecae, fertilization ducts located posteriorly, posterodorsal fold absent (Figs 69–70). *Dimensions:* Total length 8.89. Cephalothorax length 3.83, width 4.54. Sternum length 2.1, width 2.36. Abdomen length 5.06, width 4.44. Pedipalp: Fm 1.15, Pt 0.80, Ti 0.86, Ta 1.38, (total) 4.19. Leg I: Fm 4.29, Pt 1.91, Ti 3.81, Mt 3.23, Ta 1.15, (total) 14.39. Leg II: Fm 5.38, Pt 1.91, Ti 4.45, Mt 3.81, Ta 1.66, (total) 17.21. Leg III: Fm 5.58, Pt 1.72, Ti 5.92, Mt 3.70, Ta 1.53, (total) 18.45. Leg IV: Fm 5.57, Pt 1.72, Ti 4.44, Mt 3.70, Ta 1.44, (total) 16.87.

*Male* (paratype): Color: carapace yellow-brown, with slightly darker marks laterally and medially; sternum pale yellow; chelicerae pale yellow with darker infuscations anteriorly; maxillae pale yellow-brown, lightening distally; labium pale yellow-brown, lightening distally; abdomen dorsally pale creamy-yellow with a few darker flecks; ventrally pale yellow-brown; legs with segments clearly annulated, but annulations do not completely encircle femorae, legs darkening distally at tibiae; annulations lighter in centers giving a ‘leopard spot’ appearance. Cephalothorax:setae long and thin; 0.85 times longer than broad; fovea longitudinal, broad, very shallow. Eyes:AER nearly straight; PER slightly recurved; PME larger than AME, PLE largest, ALE smallest; eye group width 1.65; eye diameters, AME 0.19, ALE 0.11, PME 0.27, PLE 0.34; interdistances AME-ALE 0.46, PME-PLE 0.36, ALE-PLE 0.31, AME-PME 0.1; ocular quadrangle AME-AME 0.15, PME-PME 0.61; clypeus 0.06 high. Mouthparts:chelicerae with a few stout setae medially and anteriorly; lateral boss present, smooth; promargin with 4 teeth, retromargin with 3 teeth; maxillae longer than broad, with tuft of conspicuous setae distally; labium distally rounded. Sternum: 0.93 times longer than broad, posteriorly indented. Pedipalp: femur, spination dorsal 0–1–2; retrolateral tibial apophysis with 2 processes, dorsal apophysis longer, bent ventrally at almost a right angle, tapered to a fine point ventrally, ventral apophysis, smaller, tapering; retrolateral basal cymbial process absent; cymbial scopulae absent, cymbium round to triangular, angled bottom right; conductor large, pointed at tip; embolus very long and slender, arising from a large ovoid base that tapers abruptly, beginning at 6 o’clock, terminating at 1 o’clock; MA ovoid, centrally depressed, with two processes, one small and unsclerotized, the other a sclerotized, blunt hook (Figs 71–72). Legs:leg I much shorter than legs II, III and IV; leg formula 4231; scopulae absent on all legs; tarsus I–IV with strong claw tufts; pr claws with many c.10–15 teeth, rl claws with 1 or 2 teeth; spination: leg I, Fm pr 1–1–1, d 1–1–1, rl 1–1–1; Ti d 0, v 2–2–2–2–2–2, rl 0–1; Mt v 2–2–2–2; Ti and Mt I and II with strong spines; leg II, Fm pr 1–1–1, d 1–1–1, rl 1–1–1; Ti v 2–2–2–2–2–2; Mt v 2–2–2–2; leg III, Fm pr 1–1–1, d 1–1–1, rl 1–1–1; Ti v 2–2–1, rl 0–1; Mt 2–1; leg IV, Fm pr 1–1–1, d 1–1–1, rl 1–1–1; Ti pr 1–0–0, v 2–2–2–2, rl 1–1–1; Mt 2–1–1. Abdomen:terminal setal tufts present. *Dimensions:* Total length 6.31. Cephalothorax length 3.46, width 4.07. Sternum length 2, width 2.16. Abdomen length 2.85, width 3.25. Pedipalp: Fm 1.08, Pt 0.62, Ti 0.57, Ta 1.15, (total) 3.42. Leg I: Fm 4.44, Pt 1.81, Ti 3.55, Mt 4.07, Ta 1.91, (total) 15.78. Leg II: Fm 5.36, Pt 1.91, Ti 4.96, Mt 4.44, Ta 1.81, (total) 18.48. Leg III: Fm 5.62, Pt 1.99, Ti 4.91, Mt 4.25, Ta 1.73, (total) 18.40. Leg IV: Fm 5.53, Pt 1.68, Ti 5.15, Mt 4.44, Ta 1.90, (total) 18.70.

###### Natural history.

Collected from under rocks on a scree slope (Figs 95, 101).

###### Distribution.

The type locality only (Map 6).

##### Karaops ellenae sp. n.

urn:lsid:zoobank.org:act:FAE8E54B-AF4A-433A-A803-F8F75B0977AD

http://species-id.net/wiki/Karaops_ellenae

**Figures 73–80.**
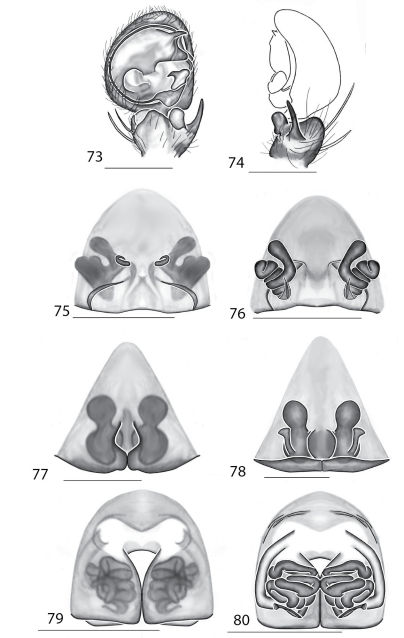
Copulatory organs of *Karaops ellenae* sp. n., male holotype from Mount Cooke, Western Australia, Australia (WAM T93/1366) (**73–74**), female paratype from Mount Cooke, Western Australia, Australia (WAM T93/1359) (**75–76**), *Karaops jenniferae* sp. n., female holotype from Oscar Range, Western Australia, Australia (WAM T65078) (**77–78**), and *Karaops dawara* sp. n., female holotype from Kakadu National Park, Kapalga, Northern Territory, Australia (WAM T54998) (**79–80**), **73** male pedipalp, ventral view **74** male pedipalp, retrolateral view **75, 77, 79** epigyne, ventral view **76, 78, 80** spermathecae, dorsal view. Scale bar: 0.50 mm.

Figs 73–76 110 Map 6

###### Type material.

Holotype male (WAM T93/1366): Mount Cooke, 32°25'S, 116°18'E, Western Australia, Australia, 25.II.1992, M.S. Harvey, J.M. Waldock. Paratype: Mount Cooke 32°25'S, 116°18'E, Western Australia, Australia, 1.X.1990, M.S. Harvey, J.M. Waldock, 1♀ (WAM T93/1359).

###### Other material examined.

**AUSTRALIA: Western Australia:** Beraking Brook Crossing, 32°10'S, 116°25'E, 1.X.1995, J.M. Waldock, under wooden plank, 1♀ (WAM T54987); west of Beraking Brook Crossing, 32°10'S, 116°25'E, 3.X.1994, J.M. Waldock, K. Brimmell, Y. Konishi, under granite rock at base of outcrop, 1♂ (WAM T54986); Canning Dam, Turtle Creek, 32°09'16"S, 116°07'17"E, 26.VIII.2003, V. Framenau, under rock on rocky outcrop, 1♂ (WAM T56032); Darlington, 31°55'S, 116°05'E, 5.IX.1962, E.S. Ross, D. Cavagnaro, 4♀ (CAS 9031586); Darlington, 31°55'S, 116°05'E, 1.I.1966, G.H. Lowe, 1♀ (WAM T93/1351); Darlington, 31°55'S, 116°05'E, 1.I.1972, G.H. Lowe), 1♀ (WAM T93/135); Darlington, 31°55'S, 116°05'E, 19.XI.1979, T. Crawford, 1 immature (WAM T T93/1353); Glenbourne Farm, south of Gracetown, 33°53'S, 115°00'E, 23.XI.1998, J.M. Waldock, 1♂ (WAM T54989); Glen Forrest, 31°55'S, 116°06'E, 3.XII.1973, S.M. Wade, 1♀ (WAM T T93/1354); Gosnells, 32°04'S, 116°00'E, 1.VIII.1969, G. Power, 1♀ (WAM T93/1355); Jarrahdale (Alcoa) Mine area, 32°16'S, 116°06'E, 5.X.1997, K.E.C. Brennan, pitfall, 1♀ (WAM T55033); Jarrahdale (Alcoa) Mine area, near Wungong Dam, junction of Haul and Phillips Roads, 32°14'S, 116°04'E, VIII.1997, K.E.C. Brennan), on side of stump, jarrah forest, unburnt for 8 years, 1♂ (WAM_55032); Mt. Cooke 32°25'S, 116°18'E, Western Australia, Australia, 1.X.1990, M.S. Harvey, J.M. Waldock, 1♀ (WAM T93/1359); Mt. Cooke, 32°25'S, 116°18'E, 7.VIII.1990, M.S. Harvey, J.M. Waldock, M. Peterson, 2♀, 1♂, 3 immatures (WAM T T93/1360- T93/1365); Mount Cooke, 32°25'S, 116°18'E, 19.IX.1991, M.S. Harvey, J.M. Waldock, under rock, 1♀, 1 immature (WAM T T93/1356, T T93/1357); Mount Cooke, 32°25'S, 116°18'E, 18.IX.1995, J.M. Waldock, A. Sampey, under granite rock, 1♀ (WAM T54990); Mount Dale, quarry site on southeast slope, 32°08'S, 116°08'E, 30.IX.1996, J.M. Waldock, under granite rock, 1♂ (WAM T54991); Mount Dale, southwest slopes, 32°07'S, 116°17'E, 27.IX.1998, J.M. Waldock, under rocks, 1♂ (WAM T76593); Serpentine National Park, Serpentine Falls, 32°22'S, 116°00'E, 9.IX.2006, M. Rix, J. Wojcieszeck, 1♂ (WAM T77333); South edge of Beraking Brook, Smith Road, 6.5 km southeast of Mount Dale, 32°08'S, 116°21'E, 26.IX.1999, J.M. Waldock, A. Sampey, under granite slab, 1♀ (WAM T54988); Waylunga Pool, 31°44'S, 116°04'E, VII.1954, W.H. Butler, 1♀ (WAM T54992).

###### Etymology.

This species is named in honor of the second author’s (MSH) daughter, Ellen Harvey.

###### Diagnosis.

Males of this species can be separated from others by the irregular shaped and unsclerotized MA, and the conductor has a pointed projection terminally (Fig. 73). Females of this species can be distinguished from others by the medially located copulatory organs and the widely spaced epigynal pockets (Fig. 75).

###### Description.

*Male (holotype):*Color: carapace yellow-brown, with slightly darker marks medially; sternum pale yellow-brown; chelicerae pale yellow with darker infuscations anteriorly and laterally; maxillae pale yellow-brown; labium pale brown; abdomen dorsally yellow-brown with red-brown and grey markings; ventrally pale yellow-brown; legs with segments clearly annulated, annulations do not completely encircle femorae, legs darkening distally at tibiae; annulations lighter in centers giving a ‘leopard spot’ appearance. Cephalothorax: setae long and thin; 0.88 times longer than broad; fovea longitudinal, broad, very shallow. Eyes:anterior AER slightly recurved; PER recurved; PME larger than AME, PLE largest, ALE smallest; eye group width 1.54; eye diameters, AME 0.19, ALE 0.12, PME 0.24, PLE 0.26; interdistances AME-ALE 0.34, PME-PLE 0.25, ALE-PLE 0.17, AME-PME 0.05; ocular quadrangle AME-AME 0.51, PME-PME 1.2; clypeus 0.1 high. Mouthparts:chelicerae with a few stout setae medially and anteriorly; lateral boss present, smooth; promargin with 3 teeth, retromargin with 2 teeth; maxillae longer than broad, with tuft of conspicuous setae distally; labium distally rounded. Sternum: 0.9 times longer than broad, posteriorly indented. Pedipalp:femur, spination dorsal 1–2–1; retrolateral tibial apophysis with 2 processes, dorsal apophysis long, tapered and pointed at tip, ventral apophysis short, broad, rounded and flattened; retrolateral basal cymbial process present; cymbial scopulae absent, cymbium round to triangular, angled bottom right; conductor large, crescent-shaped, with two terminal processes, the anterior process is slightly curved and pointed, the posterior process is rounded; embolus very long and slender, arising from an ovoid base that tapers abruptly, beginning at 6 o’clock, terminating at 1 o’clock; MA amorphous, slightly sclerotized, with one pointed process (Figs 73–74). Legs:leg I only slightly shorter than legs II, III and IV; leg formula 3241; scopulae absent on all legs; tarsus I–IV with strong claw tufts; pr claws with c. 10 teeth, rl claws with none; spination: leg I, Fm pr 1–1–0, d 1–1–1, rl 0; Ti d 0, v 2–2–2–2–2; Mt v 2–2–2; Ti and Mt I and II with strong spines; leg II, Fm pr 0, d 1–1–1, rl 0–1–1; Ti v 2–2–2–2–2; Mt v 2–2–2; leg III, Fm pr 0, d 1–1–1, rl 0–1–1; Ti 0; Mt 0; leg IV, Fm pr 0, d 1–1–1, rl 0; Ti 0; Mt 0. Abdomen:terminal setal tufts present. *Dimensions:* Total length 5.15. Cephalothorax length 2.75, width 3.12. Sternum length 1.47, width 1.63. Abdomen length 2.49, width 2.06. Pedipalp: Fm 0.79, Pt 0.41, Ti 0.38, Ta 0.77, (total) 2.35. Leg I: Fm 3.49, Pt 1.32, Ti 2.89, Mt 2.62, Ta 1.43, (total) 11.75. Leg II: Fm 4.28, Pt 1.28, Ti 3.45, Mt 2.79, Ta 1.63, (total) 13.43. Leg III: Fm 4.22, Pt 1.25, Ti 3.59, Mt 3.09, Ta 1.49, (total) 13.64. Leg IV: Fm 4.11, Pt 1.09, Ti 3.39, Mt 3.22, Ta 1.49, (total) 13.30.

*Female* (paratype)*:* Color: carapace yellow-brown, with slightly darker marks medially; sternum pale yellow-brown; chelicerae pale yellow with darker infuscations anteriorly and laterally; maxillae pale yellow-brown; labium pale brown; abdomen dorsally yellow-brown with darker markings; ventrally pale yellow-brown; legs with all segments clearly annulated. Cephalothorax:setae long and thin; 0.89 times longer than broad; fovea longitudinal, broad, very shallow. Eyes:AER slightly recurved; PER recurved; PME larger than AME, PLE largest, ALE smallest; eye group width 2.09; eye diameters, AME 0.22, ALE 0.14, PME 0.25, PLE 0.31; interdistances AME-ALE 0.57, PME-PLE 0.37, ALE-PLE 0.12, AME-PME 0.08; ocular quadrangle AME-AME 0.66, PME-PME 1.65; clypeus 0.11 high. Mouthparts:chelicerae with a few stout setae medially and anteriorly; lateral boss present, smooth; promargin with 3 teeth, retromargin with 2 teeth; maxillae longer than broad, with tuft of conspicuous setae distally; labium distally rounded. Sternum:0.89 times longer than broad, posteriorly indented. Pedipalp:tarsus slightly swollen, claw present, without teeth. Legs:leg I only slightly shorter than legs II, III and IV; leg formula 2341; scopulae absent on all legs; tarsus I–IV with strong claw tufts; pr claws with c. 10 teeth, rl claws with none; spination: leg I, Fm pr 1–1–0, d 1–1–1, rl 0; Ti d 0, v 2–2–2–2–2; Mt v 2–2–2; Ti and Mt I and II with strong spines; leg II, Fm pr 0, d 1–1–1, rl 0; Ti v 2–2–2–2–2; Mt v 2–2–2; leg III, Fm pr 0, d 1–1–1, rl 0; Ti 0; Mt 0; leg IV, Fm pr 0–0–1, d 1–1–1, rl 0; Ti 0; Mt 0. Abdomen:terminal setal tufts present. Epigyne:lateral lobes fused, copulatory openings located in center of plate, large epigynal pockets present; internally, ducts coiling 4–5 times leading to small, oblong, ovoid spermathecae, fertilization ducts located posteriorly, posterodorsal fold absent (Figs 75–76). *Dimensions:* Total length 10.28. Cephalothorax length 3.74, width 4.21. Sternum length 1.99, width 2.23. Abdomen length 6.39, width 5.14. Pedipalp: Fm 0.89, Pt 0.63, Ti 0.65, Ta 0.85, (total) 3.08. Leg I: Fm 3.83, Pt 1.61, Ti 3.39, Mt 2.37, Ta 1.32, (total) 12.52. Leg II: Fm 4.79, Pt 1.89, Ti 3.82, Mt 2.75, Ta 1.37, (total) 14.62. Leg III: Fm 4.98, Pt 1.69, Ti 3.89, Mt 2.53, Ta 1.35, (total) 14.44. Leg IV: Fm 4.75, Pt 1.42, Ti 3.61, Mt 3.11, Ta 1.38, (total) 14.27.

###### Natural history.

Collected from beneath bark, rocks and other debris.

###### Distribution.

Along the west coast of southwest Australia (Map 6).

##### Karaops jenniferae sp. n.

urn:lsid:zoobank.org:act:DB7CC137-5009-43F8-B6F2-3E39D66F28FB

http://species-id.net/wiki/Karaops_jenniferae

Figs 77–78 Map 5

###### Type material.

Holotype female (WAM T65078): Oscar Range, Western Australia, Australia, 17°38'16"S, 125°10'08"E, 26.VII.2005, B. Maryan, active at night on limestone.

###### Etymology.

The specific epithet is named in honor of the first author’s (SCC) sister, Jennifer Crews.

###### Diagnosis.

This species can be differentiated from all others by the small round median septum and the lateral lobes comeing into contact posteriorly. The internal ducts are not coiled and the spermathecae are ovoid and slightly pinched medially (Fig. 78). Males unknown.

###### Description.

*Holotype:*Color: carapace yellow-brown, with slightly darker marks laterally and medially; sternum pale yellow; chelicerae yellow-brown, slightly darker brown near anteriorly near lat boss; maxillae pale yellow-brown, lightening distally; labium pale yellow-brown, lightening distally; abdomen dorsally pale creamy-yellow with a few darker flecks; ventrally pale yellow-brown; legs with femora, patellae and tibiae I–IV clearly annulated, yellow-brown, darkening distally; annulations not encircling entire legs. Cephalothorax:setae short, stout, rodlike; 0.71 times longer than broad; fovea longitudinal, broad, very shallow. Eyes:AER slightly recurved; PER recurved; PME larger than AME, PLE largest, ALE smallest; eye group width 1.89; eye diameters, AME 0.23, ALE 0.19, PME 0.38, PLE 0.57; interdistances AME-ALE 0.48, PME-PLE 0.36, ALE-PLE 0.46, AME-PME 0.06; ocular quadrangle AME-AME 0.19, PME-PME 0.61; clypeus 0.11 high. Mouthparts:chelicerae with a few stout setae medially and anteriorly; lateral boss present, smooth; promargin with 3 teeth, retromargin with 2 teeth; maxillae longer than broad, with tuft of conspicuous setae distally; labium distally rounded. Sternum:0.91 times longer than broad, posteriorly indented. Pedipalp:tarsus slightly swollen, claw present with c. 6 teeth. Legs:leg I slightly shorter than II and III; leg formula 3214; scopulae absent on all legs; tarsus I–IV with strong claw tufts; claws without teeth; spination: leg I, Fm pr 1–1–0, d 1–1–1, rl 0; Ti d 0, v 2–2–2–2–2; Mt v 2–2–2; Ti and Mt I and II with strong spines; leg II, Fm pr 0, d 1–1–1, rl 0; Ti v 2–2–2–2–2; Mt v 2–2–2; leg III, Fm pr 0, d 1–1–1, rl 0; Ti v 1–1–0; Mt 1–0; leg IV, Fm pr 0, d 1–1–1, rl 0–0–1; Ti v 1–1; Mt 0. Abdomen:abdomen damaged. Epigyne:lateral lobes surrounding a small, subquadrate median area, lobes coming into contact posteriorly, copulatory openings located anteromedially, epigynal pockets absent; internally, no ducts, just large ovoid spermathecae, fertilization ducts located posteriorly, posterodorsal fold present, small, barely covering any of the internal copulatory organs (Figs 77–78). *Dimensions:* Total length 8.92. Cephalothorax length 3.28, width 4.63. Sternum length 2.03, width 2.22. Abdomen length 5.64, width 4.91. Pedipalp: Fm 1.07, Pt 0.77, Ti 0.88, Ta 1.24, (total) 3.96. Leg I: Fm 5.62, Pt 1.91, Ti 4.96, Mt 4.07, Ta 1.82, (total) 18.38. Leg II: Fm 5.64, Pt 2.28, Ti 5.01, Mt 4.07, Ta 1.76, (total) 18.76. Leg III: Fm 5.92, Pt 2.01, Ti 5.15, Mt 4.29, Ta 1.61, (total) 18.98. Leg IV: Fm 5.57, Pt 1.56, Ti 4.45, Mt 4.22, Ta 1.76, (total) 17.56.

###### Natural history.

This large species has been collected from limestone rocks at night.

###### Distribution.

The type locality only (Map 5).

##### Karaops dawara sp. n.

urn:lsid:zoobank.org:act:F393CF5F-F812-4C7A-A4C1-526DEE99DBE6

http://species-id.net/wiki/Karaops_dawara

Figs 79–80 102 Map 5

###### Type material.

Holotype female (WAM T54998): Kakadu National Park, Kapalga, primary site E, 12°36'S, 132°25'E, Northern Territory, Australia, 9.XI.1990, A. Andersen et al.

###### Other material examined.

**AUSTRALIA: Northern Territory:** Darwin: Charles Darwin National Park, first left hand road after gate, 12°26'12.2"S, 130°52'36.5"E, 15–16.I.2009, S.C. Crews, G. Brown, with egg-sac, on *Pandanus*, 1♀ (WAM T97225); Litchfield National Park, off Litchfield Road, on road on left side, heading south, 13°03.024'S, 130°51.300'E, 20.I.2009, S.C. Crews, G. Brown, under bark near ‘A2’ sign along road, 1 immature (WAM T97233).

###### Etymology.

The specific epithet comes from the word for spider, *dawara*, in the indigenous Larrakia language.

###### Diagnosis.

This species can be distinguished from all others by having an abdominal pattern of light spots on a dark background, and by the copulatory organs, as the lateral lobes come into contact for nearly half of the length of the epigynal plate, and the copulatory ducts are long, laterally positioned, and lead to a mass of winding ducts (Figs 79–80). Males unknown.

###### Description.

*Holotype:*Color: carapace yellow-brown, with slightly darker marks medially; sternum pale yellow-brown; chelicerae pale yellow with darker infuscations anteriorly; maxillae pale yellow-brown; labium pale brown; abdomen dorsally dark grey, with pale patches anteriorly, dorsally and posteriorly; ventrally pale yellow-brown; legs with all segments clearly annulated. Cephalothorax:setae long and thin; 0.83 times longer than broad; fovea longitudinal, broad, very shallow. Eyes:AER nearly straight; PER slightly recurved; PME larger than AME, PLE largest, ALE smallest; eye group width 1.51; eye diameters, AME 0.17, ALE 0.09, PME 0.19, PLE 0.25; interdistances AME-ALE 0.28, PME-PLE 0.26, ALE-PLE 0.23, AME-PME 0.07; ocular quadrangle AME-AME 0.47, PME-PME 0.96; clypeus 0.18 high. Mouthparts: chelicerae with a few stout setae medially and anteriorly; lateral boss present, smooth; promargin with 4 teeth, retromargin with 3 teeth; maxillae longer than broad, with tuft of conspicuous setae distally; labium distally rounded. Sternum:0.85 times longer than broad, posteriorly indented. Pedipalp:tarsus slightly swollen, claw present, with c. 6 teeth. Legs:leg I only slightly shorter than legs II, III and IV; leg formula 2341; scopulae absent on all legs; tarsus I–IV with strong claw tufts; claws without teeth; spination: leg I, Fm pr 1–1–0, d 1–1–1, rl 0; Ti d 0, v 2–2–2–2–2; Mt v 2–2–2–2; Ti and Mt I and II with strong spines; leg II, Fm pr 0, d 1–1–1, rl 0; Ti v 2–2–2–2–2; Mt v 2–2–2–2; leg III, Fm pr 0, d 1–1–1, rl 0; Ti v 1–1–0; Mt 0; leg IV, Fm pr 0, d 1–1–1, rl 1–0–0; Ti pr 1–1–0, v 0, rl 1–0–0; Mt 0. Abdomen:terminal setal tufts present. Epigyne:lateral lobes that come into contact medially, forming a v-shaped opening, sinuate unsclerotized area in the upper third of plate, copulatory openings located laterally in this area; internally, long unsclerotized ducts lead away from the copulatory openings, at the lateral margins, curving medially to a mass of sclerotized coiled ducts, mostly symmetrical, leading to tiny ovoid to round spermathecae, fertilization ducts located posteriorly, posterodorsal fold absent (Figs 79–80). *Dimensions:* Total length 6.35. Cephalothorax length 2.31, width 2.78. Sternum length 1.26, width 1.48. Abdomen length 3.73, width 3.24. Pedipalp: Fm 0.67, Pt 0.44, Ti 0.58, Ta 0.66, (total) 2.35. Leg I: Fm 2.55, Pt 1.04, Ti 2.31, Mt 1.81, Ta 1.04, (total) 8.75. Leg II: Fm 3.14, Pt 1.14, Ti 5.20, Mt 2.15, Ta 1.01, (total) 12.73. Leg III: Fm 3.44, Pt 1.10, Ti 2.70, Mt 2.27, Ta 1.10, (total) 10.61. Leg IV: Fm 3.24, Pt 0.94, Ti 2.48, Mt 2.27, Ta 1.08, (total) 10.01.

###### Natural history.

This species has been found on *Pandanus*, under the fronds where they attach to the trunk, as well as under the leaves, and under bark of a log on the ground (Fig. 102).

###### Distribution.

Known only from the northern region of the Northern Territory (Fig. 102; Map 5).

#### Genus Makdiops gen. n.

urn:lsid:zoobank.org:act:173E8950-B09A-45E4-8463-5688C6C26438

http://species-id.net/wiki/Makdiops

##### Type species:

*Selenops montigenus* Simon, 1889.

##### Etymology.

*Makdiops* comes from a combination of words and is from the indigenous language of the region in which this genus is found. Hindi: मकड़ी *=* makdi (romanization) =spider; Greek: *ops* = face, eye. We retain the traditional ending of selenopid genera of *ops*, which originally referred to the eye arrangement. The gender is masculine.

##### Diagnosis.

*Makdiops* gen. n.can be separated from all other genera by a combination of characters. The ventral spination of the tibiae and metatarsi is 4–3, 3–3, or 3–2, there are no tarsal scopulae, and the genus is only found from India and Nepal.

##### Remarks.

Here we describe two new species, move three species from *Selenops* to *Makdiops* gen. n. (*Makdiops montigenus* comb. n., *Makdiops agumbensis* comb. n., *Makdiops nilgirensis* comb. n.), redescribe *Makdiops montigenus* comb. n., including the first description of the male.

It appears that at least two genera, *Makdiops* gen. n.and *Selenops*, occur throughout the Indo-Asian region (Map 1, Map 3). The species of *Selenops* include *Selenops radiatus*, the most widespread selenopid species, *Selenops sumitrae* Patel & Patel, 1973, and *Selenops shevaroyensis* Gravely, 1931. We were unable to examine specimens of the latter two species, however the published descriptions and illustrations of *Selenops sumitrae* (Patel & Patel 1973) make it difficult to differentiate from *Selenops radiatus*, and if it is not a synonym, they are certainly very closely related. The description and illustration of *Selenops shevaroyensis* are inadequate (Gravely 1931) and the type is not available. At this time, we will make no taxonomic changes to this species, pending the collection of new material.

It is likely that several more species from this region will be found with further exploration, and it is of note that the male of only one species of *Makdiops* gen. n. is known. Most of these are known from only a single specimen, and in cases where they are not, there seems to be a lot of variation. While it is possible that these species may represent more than one genus, at this time, we will group them together based on their geographic locations, genitalic similarities, and lack of tarsal scopulae.

##### Description.

Total length 6.70–9.70. *Cephalothorax:* Carapace with some dark spots or dusky markings, wider than long. Fovea longitudinal, short, broad, and shallow. Setae variable, ranging from short and spine-like, to long and thin; some are of medium length and thickness. AER straight, PER slightly recurved to recurved. PME larger than AME. Chelicerae slightly geniculate, robust, with 3 prolateral and 2 retrolateral teeth. *Legs:* Leg II or III longest, with III usually longer than IV. Tibial and metatarsal ventral spination variable, either 4–3, 3–3, or 3–2. Tarsal scopulae absent. *Female copulatory organs:* Epigynum with lateral lobes, a well-defined median area, and with or without epigynal pockets. Spermathecae range from being simple and not coiled, to some coiling, to extremely coiled and asymmetrical. Posterodorsal fold present or not. *Male copulatory organs:* The male of only one species is known. Palpal tibia with 1 tibial bifid apophysis. Dorsal portion longer, thin and slightly curved; ventral portion shorter and flattened; MA small, simple and single-branched; Conductor large, T-shaped, pointed retrolaterally.

##### Distribution.

*Makdiops* gen. n.occurs throughout India and Nepal (Map 3). It has been found at a higher elevation than any other selenopid species, at over 2500 m. It is likely to be found in other countries throughout the region (Map 3).

##### Composition.

The genus contains five species: *Makdiops montigenus* (Simon, 1889)comb. n., *Makdiops agumbensis* (Tikader, 1969)comb. n., *Makdiops nilgirensis* (Reimoser, 1934)comb. n., *Makdiops shiva* sp. n. and *Makdiops mahishasura* sp. n.

##### Key to Makdiops species

**Table:** 

1	Tibiae I and II with 4 pairs of ventral spines	2
–	Tibiae I and II with 3 pairs of ventral spines	3
2(1)	Epigynal pockets not reaching sinuous margin covering genital openings (Tikader 1969, Fig. 2)	*Makdiops agumbensis*
–	Epigynal pockets reaching margin covering copulatory openings (Fig. 89)	*Makdiops shiva* sp. n.
3(1)	Very large posterodorsal fold covering internal ducts and spermathecae (Fig. 88)	*Makdiops nilgirensis*
–	Posterodorsal fold absent	4
4(3)	Internal ducts asymmetrical, convoluted, twisting numerous times (Fig. 82); male with a single bifid RTA, small hook-shaped MA, and large T-shaped conductor terminating at 3 o’clock (Figs 83–84)	*Makdiops montigenus*
–	Internal ducts only twisted 3 or so times, internal ducts symmetrical (Fig. 86)	*Makdiops mahishasura* sp. n

##### Makdiops montigenus

(Simon, 1889) comb. n.

http://species-id.net/wiki/Makdiops_montigenus

**Figures 81–88.**
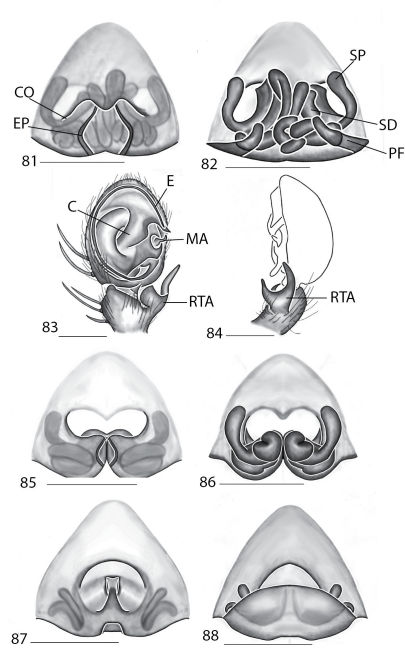
Copulatory organs of *Makdiops montigenus* comb. n., female from Bhowali, Uttarakhand, India (CAS 9031784) (**81–82**), male from Bhowali, Uttarakhand, India (CAS 9031784) (**83–84**), *Makdiops mahishasura* sp. n., female holotype from Punjur, India (CAS 9031588) (**85–86**), and *Makdiops nilgirensis* sp. n., female holotype from Karteri Valley, Tamil Nadu, India (MHN) (**87–88**), **81, 85, 87** epigyne, ventral view **82, 86, 88** spermathecae, dorsal view **83** male pedipalp, ventral view **84** male pedipalp, retrolateral view. Scale bar: 0.50 mm. Abbreviations: **CO** = copulatory openings, **EP** = epigynal pockets, **SP** = spermathecae, **SD** = sperm ducts, **PF** = posterodorsal fold, **C** = conductor, **E** = embolus, **MA** = median apophysis, **RTA** = retrolateral tibial apophysis.

Figs 81–84 112 Map 3

Selenops montigena Simon 1889b: 335. Gravely 1931: Fig. 15D.

###### Type material.

Holotype female (apparently lodged in ZSI; not examined):Jaonsar, Kumia, Uttarakhand, India [30°33'N, 78°10'E], 6000 feet, Oldham. The female type was not examined, but it is clear from Gravely’s (1931) illustration that this is the same species.

###### Other material examined:

**INDIA: Himachal Pradesh:** Patlikuhl Town, 32°07.4'N, 77°08.8'E, 1200m, 17–23.VI.1999, Y. Marusik, 4♀ (ZMUM); **Jharkhand:** 6 miles northeast of Borio [25°07'N, 87°37'E], 220 m, 30.X.1961, E.S. Ross, D.Q. Cavagnaro, 1♂ (CAS 9031587); **Uttarakhand:** Bhowali [29°23'N, 79°31'E], 1800 m, 1.XII.1961, E.S. Ross, D.Q. Cavagnaro, 1♂, 1♀ (CAS 9031784); 5 miles southwest of Dehra Dun [30°15'N, 77°56'E], 600 m, 9.XII.1961, E.S. Ross, D.Q. Cavagnaro, 1♂ (CAS 9031785); 8 miles southwest Dehra Dun [30°15'N, 77°56'E], 750 m, 11.XII.1961, E.S. Ross, D.Q. Cavagnaro, 1♀ (CAS 9031585); Gobind Ghat Village, 30°37.5'N, 79°33.5'E, 1750–1900 m, 17–23.V.1999, Y. Marusik, 2♀, 2 immature (ZMUM); Joshimath Town, 30°33.3'N, 79°33.9'E, 1870 m, 14.V.1999, Y Marusik, 1♀ (ZMUM). **NEPAL**: Chitwan National Park, near Sauraha [27°29'N, 84°21'E], 1♂ (CAS sel_985).

###### Literature records.

**INDIA: Jharkhand:** Chota Nagpur, pass between Chaibassa and Chaardharpur [22°35'N, 85°43'E] (Gravely 1931); **Uttarakhand:** Painsur, above Lohba [30°04'N, 79°19'E], 8000 feet (Gravely 1931).

###### Diagnosis.

Males can be separated from other selenopids by the RTA, a single, bifid process, the large T-shaped conductor, and the small, hooked MA (Figs 83–84). In females, the internal ducts are convoluted and laterally asymmetrical (Fig. 82).

###### Description.

*Female* (CAS 9031784)*:*Color: carapace uniformly red-brown; sternum yellow-brown, darker around border; chelicerae uniformly dark red-brown; maxillae brown, lightening distally; labium pale yellow-brown, lightening distally; abdomen dorsally cream colored with darker cardiac mark and some darker w-shaped marks caudally, festoon mark also present caudally; ventrally pale yellow-brown; legs tan, darkening distally, no annulations visible. Cephalothorax: setae long and thin; 0.91 times longer than broad; fovea longitudinal, broad, somewhat shallow. Eyes: AER nearly straight; PER slightly recurved; PME larger than AME, PLE largest, ALE smallest; eye group width 2.18; eye diameters, AME 0.17, ALE 0.13, PME 0.23, PLE 0.31; interdistances AME-ALE 0.57, PME-PLE 0.55, ALE-PLE 0.38, AME-PME 0.15; ocular quadrangle AME-AME 0.27, PME-PME 0.92; clypeus 0.06 high. Mouthparts:chelicerae with a few stout setae medially and anteriorly; lateral boss present, smooth; promargin with 3 teeth, retromargin with 2 teeth; maxillae longer than broad, with tuft of conspicuous setae distally; labium distally rounded. Sternum:0.96 times longer than broad, posteriorly indented. Pedipalp: claw present, without teeth. Legs:leg I much shorter than legs II, III and IV; leg formula 2341; some scopulae on tarsus I and distally on metatarsus I; tarsus I–IV with strong claw tufts; pr claw with c. 10 teeth, rl claw with none; spination: leg I, Fm pr 1–1–0, d 1–1–1, rl 1–0–0; Ti v 2–2–2; Mt v 2–2–2; Ti and Mt I and II with strong spines; leg II, Fm pr 0, d 1–1–1, rl 0; Ti 2–2–2–2; Mt v 2–2–2; leg III, Fm pr 0, d 1–1–1, rl 0; Ti v 1–1–0; Mt 2–0; leg IV, Fm pr 0, d 1–1–1, rl 0; Ti v 1–1; Mt 1–0. Abdomen:terminal setal tufts present. Epigyne:sinusoidal m-shaped ridge located medially, copulatory openings underneath, epigynal pockets present; internally, ducts are convoluted, laterally asymmetrical, differing in their folding amongst specimens, leading to long spermathecae at the lateral margins, small posterodorsal infoldings (Figs 81–82). *Dimensions:* Total length 9.31. Cephalothorax length 4.07, width 4.46. Sternum length 2.20, width 2.30. Abdomen length 5.24, width 4.07. Pedipalp: Fm 1.34, Pt 0.80, Ti 0.82, Ta 1.34, (total) 4.30. Leg I: Fm 4.40, Pt 2.30, Ti 3.51, Mt 3.17, Ta 1.40, (total) 14.78. Leg II: Fm 5.38, Pt 2.34, Ti 4.44, Mt 3.70, Ta 1.49, (total) 17.35. Leg III: Fm 5.36, Pt 1.97, Ti 4.45, Mt 3.51, Ta 1.34, (total) 16.63. Leg IV: Fm 5.15, Pt 1.72, Ti 3.7, Mt 3.52, Ta 1.34, (total) 15.43.

*Male* (CAS sel_985)*:* Color: carapace uniformly red-brown; sternum yellow brown, darker around border; chelicerae uniformly dark red-brown; maxillae brown, lightening distally; labium pale brown; abdomen dorsally cream colored with darker cardiac mark and some darker w-shaped marks and festoon caudally; ventrally pale yellow-brown; legs tan, darkening distally, no annulations visible. Cephalothorax:setae long and thin; 0.91 times longer than broad; fovea longitudinal, broad, somewhat shallow. Eyes:AER nearly straight; PER slightly recurved; PME larger than AME, PLE largest, ALE smallest; eye group width 2.24; eye diameters, AME 0.23, ALE 0.13, PME 0.29, PLE 0.38; interdistances AME-ALE 0.52, PME-PLE 0.50, ALE-PLE 0.40, AME-PME 0.10; ocular quadrangle AME-AME 0.25, PME-PME 0.86; clypeus 0.06 high. Mouthparts:chelicerae with a few stout setae medially and anteriorly; lateral boss present, smooth; promargin with 3 teeth, retromargin with 2 teeth; maxillae longer than broad, with tuft of conspicuous setae distally; labium distally rounded. Sternum:0.96 times longer than broad, posteriorly indented. Pedipalp:femur, spination dorsal 0–1–2; retrolateral tibial apophysis with 1 apophysis that is bifid, the posterior process longer, tapering and slightly truncate at the tip, the ventral process shorter, pointier; retrolateral basal cymbial process absent; cymbial scopulae present, cymbium oval; conductor emerging from center of bulb on a long stalk, T-shaped, tapering to a point, pointed laterally; embolus very long and slender, beginning at 4 o’clock, terminating at 2 o’clock; median apophysis small, hooked on end, directed distally (Figs 83–84). Legs:leg I much shorter than leg I but only slightly shorter than leg III; leg formula 2314; scopulae absent on all legs; tarsus I–IV with strong claw tufts; pr claws with c.10–15 teeth, rl claw lacking teeth; spination: leg I, Fm pr 1–1–0, d 1–1–1, rl 1–1–1; Ti v 2–2–2, rl 1–0–0; Mt v 2–2–2; Ti and Mt I and II with strong spines; leg II, Fm pr 1–0–0, d 1–1–1, rl 1–1–1; Ti 2–2–2; Mt v 2–2–2; leg III, Fm pr 1–0–0, d 1–1–1, rl 1–1–1; Ti v 1–1–0; Mt 2–0; leg IV, Fm pr 0, d 1–1–1, rl 0; Ti v 1–1; Mt 1–0. Abdomen:terminal setal tufts present. *Dimensions:* Total length 9.78. Cephalothorax length 4.25, width 4.68. Sternum length 2.40, width 2.50. Abdomen length 5.53, width 4.44. Pedipalp: Fm 1.68, Pt 0.94, Ti 0.77, Ta 1.62, (total) 5.01. Leg I: Fm 6.04, Pt 2.48, Ti 5.70, Mt 5.13, Ta 1.83, (total) 21.18. Leg II: Fm 7.99, Ti 7.41, Mt 6.77, Ta 1.94, (total) 26.75. Leg III: Fm 7.33, Ti 5.62, Mt 5.15, Ta 1.59, (total) 21.85. Leg IV: Fm 6.15, Pt 2.19, Ti 5.36, Mt 5.38, Ta 1.66, (total) 20.75.

###### Natural history.

Collected under bark and rocks, and taken from over 2500 m elevation.

###### Distribution.

This species is found in northern India and Nepal, near the Himalaya Mountains (Map 3).

##### Makdiops agumbensis

(Tikader, 1969) comb. n.

http://species-id.net/wiki/Makdiops_agumbensis

Selenops agumbensis Tikader 1969: 252, figs 1–3.

###### Type material.

Holotype female (ZSI; not examined):Agumbe Ghat, District Shimoga, Mysore, India, B.K. Tikader, 15.III.1965.

###### Remarks.

We were unable to examine the type of *Makdiops agumbensis* comb. n. From the description and illustrations (Tikader 1969), it is impossible to tell whether or not it is valid, or whether it is indeed a unique species. However, the description suggests it is a species of *Makdiops* rather than *Selenops*, viz,tibiae I and II have 4 pairs of ventral spines, the epigyne has large epigynal pockets, and the genital openings are located behind a sinuous margin (Tikader 1969; fig. 2).

##### Makdiops mahishasura sp. n.

urn:lsid:zoobank.org:act:A7B8D65A-AC6C-4DD6-BCB8-1E8BD72DE8C2

http://species-id.net/wiki/Makdiops_mahishasura

Figs 85–86 Map 3

###### Type material.

Holotype female (CAS 9031589): 2 miles northwest of Punjur, Karnataka, India [11°50'N, 77°06'E], 850 m, 13.III.1962, E.S. Ross, D.Q. Cavagnaro.

###### Other material examined.

**INDIA: Karnataka:** 8 miles west of Hunsur [12°18'N, 76°10'E], 800 m, 2.II.1962, E.S. Ross, D.Q. Cavagnaro, 4♀ (CAS 9031588).

###### Etymology.

The specific epithet comes from the Kannada word ಮೈಸೂರ = Maisūru referring to Mahishasura, a Hindu asura, for which the city of Mysore, or the region of the type locality, was named. The name is to be treated as a noun in apposition.

###### Diagnosis.

This species can be differentiated from all others by a combination of characters including tibiae I and II with three pairs of ventral spines, sperm ducts only coiled a few times, and ducts symmetrical (Fig. 86). Males unknown.

###### Description.

*Holotype.*Color: carapace red-brown, darker marks laterally and mediolaterally; sternum yellow brown, darker around border; chelicerae red-brown with darker infuscations medially and laterally; maxillae pale yellow-brown, lightening distally; labium pale brown, lightening distally; abdomen dorsally yellow brown with darker flecks medially and laterally, festoon prominent; ventrally pale yellow-brown; legs orange-brown with annulations on femora, patella and tibia, darkening distally. Cephalothorax:setae short, stout, and rodlike; 0.81 times longer than broad; fovea longitudinal, broad, somewhat shallow. Eyes:AER nearly straight; PER slightly recurved; PME larger than AME, PLE largest, ALE smallest; eye group width 1.72; eye diameters, AME 0.19, ALE 0.13, PME 0.27, PLE 0.31; interdistances AME-ALE 0.46, PME-PLE 0.38, ALE-PLE 0.34, AME-PME 0.1; ocular quadrangle AME-AME 0.17, PME-PME 0.69; clypeus 0.1 high. Mouthparts:chelicerae with a few stout setae medially and anteriorly; lateral boss present, smooth; promargin with 3 teeth, retromargin with 2 teeth; maxillae longer than broad, with tuft of conspicuous setae distally; labium distally rounded. Sternum:0.9 times longer than broad, posteriorly indented. Pedipalp: claw present with c. 6 teeth. Legs:leg I much shorter than legs II, III and IV; leg formula 3421; scopulae absent on all legs; tarsus I–IV with strong claw tufts; pr claw with c.10–15 teeth, rl claw lacking teeth; spination: leg I, Fm pr 1–1–0, d 1–1–1, rl 0; Ti v 2–2–2; Mt 2–2; Ti and Mt I and II with strong spines; leg II, Fm pr 0, d 1–1–1, rl 0; Ti 2–2–2; Mt 2–2; leg III, Fm pr 0, d 1–1–1, rl 0; Ti 1–0–0; Mt 2–0; leg IV, Fm pr 0, d 1–1–1, rl 0; Ti v 1–0–0; Mt 0. Abdomen:terminal setal tufts present. Epigyne:lateral lobes come together posteriorly, slightly sinuous to oblong opening in middle of plate, copulatory openings located posteriorly, epigynal pockets present; internally, ducts are coiled several times leading to long, spermathecae located laterally, posterodorsal fold absent (Figs 85–86). *Dimensions:* Total length 8.55. Cephalothorax length 3.26, width 4.03. Sternum length 1.89, width 2.10. Abdomen length 5.29, width 4.40. Pedipalp: Fm 1.05, Pt 0.77, Ti 0.84, Ta 0.96, (total) 3.62. Leg I: Fm 3.52, Pt 1.72, Ti 2.86, Mt 2.30, Ta 1.17, (total) 11.61. Leg II: Fm 4.25, Pt 1.76, Ti 3.43, Mt 2.64, Ta 1.30, (total) 13.38. Leg III: Fm 4.96, Pt 1.82, Ti 3.70, Mt 2.52, Ta 1.26, (total) 14.26. Leg IV: Fm 4.68, Pt 1.43, Mt 2.52, Ta 1.26, (total) 13.44.

###### Natural history.

No data.

###### Distribution.

Known only from southern India (Map 3).

##### Makdiops nilgirensis

(Reimoser, 1934) comb. n.

http://species-id.net/wiki/Makdiops_nilgirensis

Figs 87–88 113 Map 3

Selenops nilgirensis Reimoser 1934: 486, Fig. 10.

###### Type material.

Holotype female (MHNG): Karteri Valley, Tamil Nadu, India [11°18'N, 76°48'E], Voy. Carl et Escher.

###### Diagnosis.

The species can be separated from other species by the raised epigynal plate and very large posterodorsal fold (Figs 87–88). Males unknown.

###### Description.

*Holotype:*Color: carapace uniformly yellow-brown; sternum pale yellow; chelicerae pale yellow with darker infuscations anteriorly; maxillae pale yellow; labium pale yellow-brown; abdomen dorsally yellow brown with darker flecks medially and laterally, festoon prominent; ventrally pale yellow-brown; legs with femora, patellae and tibiae I-IV clearly annulated, yellow-brown, darkening distally; annulations not encircling legs entirely. Cephalothorax:setae long and thin; 0.81 times longer than broad; fovea longitudinal, broad, somewhat shallow. Eyes:AER nearly straight; PER recurved; PME larger than AME, PLE largest, ALE smallest; eye group width 1.53; eye diameters, AME 0.17, ALE 0.10, PME 0.25, PLE 0.29; PME-PLE 0.34, ALE-PLE 0.33, AME-PME 0.08; ocular quadrangle AME-AME 0.21, PME-PME 0.63; clypeus 0.06 high. Mouthparts:chelicerae with a few stout setae medially and anteriorly; lateral boss present, smooth; promargin with 3 teeth, retromargin with 2 teeth; maxillae longer than broad, with tuft of conspicuous setae distally; labium distally rounded. Sternum:0.94 times longer than broad, posteriorly indented. Pedipalp:claw present with c. 6 teeth. Legs:leg I much shorter than legs II, III and IV; leg formula 32=41; scopulae absent on all legs; tarsus I–IV with strong claw tufts; pr claws with c. 10 teeth, rl claw with none; spination: leg I, Fm pr 1–1–0, d 1–1–1, rl 0; Ti v 2–2–2; Mt 2–2; Ti and Mt I and II with strong spines; leg II, Fm pr 0, d 1–1–1, rl 0; Ti 2–2–2; Mt 2–2; leg III, Fm pr 0, d 1–1–1, rl 0; Ti 1–0–0; Mt 1–0; leg IV, Fm pr 0, d 1–1–1, rl 0; Ti v 1–0 L, 0 R; Mt 0. Abdomen:terminal setal tufts present. Epigyne:lateral lobes not distinct, medial arch anterior to w-shaped area, median area in between this raised, small quadrangular area in center of plate, epigynal pockets present; internally, large posterodorsal fold present, and as this is the type of a rare specimen, we chose not to dissect it, spermathecae seen through integument are oblong and narrow, fertilization ducts located posterolaterally (Figs 87–88). *Dimensions:* Total length 6.65. Cephalothorax length 2.95, width 3.62. Sternum length 1.74, width 1.86. Pedipalp: Fm 0.96, Pt 0.48, Ti 0.63, Ta 0.96, (total) 3.03. Leg I: Fm 3.34, Pt 1.38, Ti 2.73, Mt 2.30, Ta 1.13, (total) 10.88. Leg II: Fm 4.25, Pt 1.55, Mt 2.57, Ta 1.17, (total) 12.53. Leg III: Fm 4.59, Pt 1.43, Ti 3.40, Mt 2.64, Ta 1.26, (total) 13.32. Leg IV: Fm 4.40, Pt 1.32, Ti 3.14, Mt 2.52, Ta 1.15, (total) 12.53.

###### Natural history.

No data.

###### Distribution.

The type locality only (Map 3).

##### Makdiops shiva sp. n.

urn:lsid:zoobank.org:act:5B82E0C8-A09E-41A2-9B99-2CD4AE065577

http://species-id.net/wiki/Makdiops_shiva

Figs 89–90 Map 3

###### Type material.

Holotype female (CAS 9031584): Bhimashankar, Maharashtra, India, 19°04'N, 73°32'E, 1–5.II.1990, V. and B. Roth.

###### Other material examined:

**India: Maharashtra** same data as holotype, 1♀ (CAS).

###### Etymology.

The specific epithet refers to the Hindu god Shiva, as the type locality is the location of one of the 12 traditional Jyotirlingas of Shiva. The name is to be treated as a noun in apposition.

###### Diagnosis.

**Figures 89–90.**
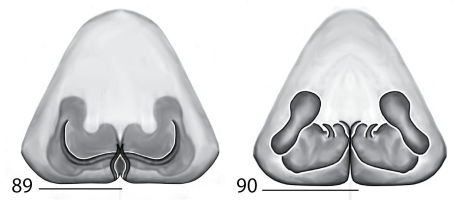
Copulatory organs of *Makdiops shiva* sp. n., female holotype from Maharashtra, Bhimashankar, India, (CAS 9031584) (**89–90**), **89** epigyne, ventral view **90** spermathecae, dorsal view. Scale bar: 0.50 mm.

This species can be differentiated from all others by having 4 pairs of ventral tibial spines on legs I and II, and by the epigynal pockets reaching the sinuous margin where copulatory openings are located (Fig. 89). Males unknown.

###### Description.

*Holotype:*Color: carapace red-brown, darker marks laterally and mediolaterally; sternum yellow brown, darker around border; chelicerae red-brown with darker infuscations medially and laterally; maxillae pale yellow-brown, lightening distally; labium pale brown, lightening distally; abdomen dorsally grey-brown with darker flecks medially and laterally, festoon present but inconspicuous; ventrally pale yellow-brown; legs yellow brown with darker annulations, legs darkening distally. Cephalothorax: setae long and thin; carapace flattened; 0.89 times longer than broad; fovea longitudinal, broad, somewhat shallow. Eyes:AER nearly straight; PER slightly recurved; PME larger than AME, PLE largest, ALE smallest; eye group width 1.99; eye diameters, AME 0.17, ALE 0.13, PME 0.23, PLE 0.27; interdistances AME-ALE 0.48, PME-PLE 0.42, ALE-PLE 0.29, AME-PME 0.1; ocular quadrangle AME-AME 0.19, PME-PME 0.75; clypeus 0.1 high. Mouthparts: chelicerae with a few stout setae medially and anteriorly; lateral boss present, smooth; promargin with 3 teeth, retromargin with 2 teeth; maxillae longer than broad, with tuft of conspicuous setae distally; labium distally rounded. Sternum:0.98 times longer than broad, posteriorly indented. Pedipalp:claw present with c. 6 teeth. Legs:leg I only slightly shorter than legs II, III and IV; leg formula 2341; scopulae absent on all legs; tarsus I–IV with strong claw tufts; pr claw with c. 10 teeth, rl claw with none; spination: leg I, Fm pr 1–1–0, d 1–1–1, rl 0; Ti v 2–2–2–2; Mt v 2–2–2–2; Ti and Mt I and II with strong spines; leg II, Fm pr 0, d 1–1–1, rl 0; Ti 2–2–2–2; Mt v 2–2–2–2; leg III, Fm pr 0, d 1–1–1, rl 0; Ti 1–0–0; Mt 2–0; leg IV, Fm pr 0, d 1–1–1, rl 0; Ti v 1–0; Mt 1–0. Abdomen:terminal setal tufts absent. Epigyne:lateral lobes coming together posteriorly, rectangular to slightly sinusoidal median area formed by lobes, epigynal pockets present; internally wide ducts lead to ovoid spermathecae, fertilization ducts located medially, posterodorsal fold absent (Figs 89–90). *Dimensions:* Total length 8.12. Cephalothorax length 3.44, width 3.85. Sternum length 1.97, width 2.01. Abdomen length 4.68, width 3.70. Pedipalp: Fm 0.96, Pt 0.65, Ti 0.71, Ta 1.11, (total) 3.43. Leg I: Fm 3.22, Pt 1.62, Ti 2.86, Mt 2.64, Ta 1.07, (total) 11.41. Leg II: Fm 4.07, Pt 1.29, Ti 3.66, Mt 2.64, Ta 1.15, (total) 12.81. Leg III: Fm 3.85, Pt 1.56, Ti 3.23, Ta 1.15, (total) 12.72. Leg IV: Fm 4.07, Pt 1.34, Ti 2.96, Mt 2.89, Ta 1.10, (total) 12.36.

###### Natural history.

No data.

###### Distribution.

The type locality only (Map 3).

**Figures 91–95.**
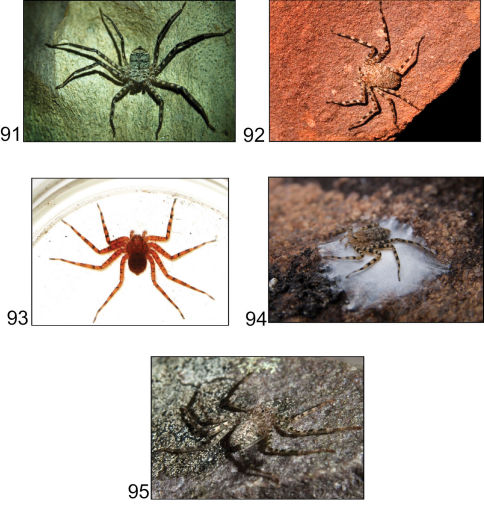
*Karaops* spp. *in situ*. **91** *Karaops raveni* sp. n., on tree at dusk, Skillion Nature Reserve, New South Wales **92** *Karaops badgeradda*sp. n., on turned rock, Badgeradda Range, Muggon Station, Western Australia **93** *Karaops martamarta* sp. n., showing red coloration, from Red Hill, Pilbara, Western Australia **94** *Karaops francesae* sp. n., female guarding eggsac, Cape Arid, Western Australia **95** *Karaops toolbrunup* sp. n., on turned rock from scree slope, Toolbrunup, Stirling Ranges, Western Australia.

**Figure 96–102.**
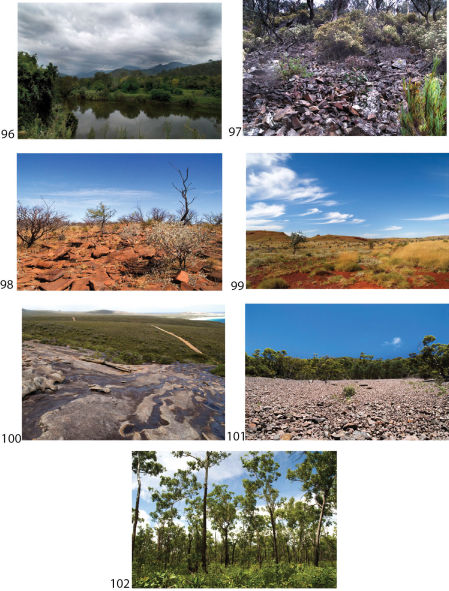
Habitats of *Karaops* species. **96** Macleay River, New South Wales, habitat of *Karaops manaayn* sp. n. and *Karaops raveni* sp. n., both collected under the bark of trees above the river **97** Scree slope in the Tinderry Ranges, New South Wales, habitat of *Karaops raveni* sp. n., collected from beneath the rocks **98 **Badgeradda Range, near Muggon Station, Western Australia, habitat of *Karaops badgeradda* sp. n., collected from under loose rocks **99** Near Red Hill, Pilbara, Western Australia, habitat of *Karaops martamarta* sp. n., collected from beneath loose rocks. These spiders are a very red color, as the color of the substrate **100 **Cape Arid, Western Australia, habitat of *Karaops francesae* sp. n., collected from beneath loose rocks on granite outcrop **101** Scree slope on Toolbrunup, Stirling Ranges National park, habitat of *Karaops toolbrunup* sp. n., collected from beneath rocks on scree slope **102** Forest at Kapalga, near South Alligator River, Kakadu National Park, Northern Territory, habitat of *Karaops dawara* sp. n., collected from beneath bark.

**Figures 103–109.**
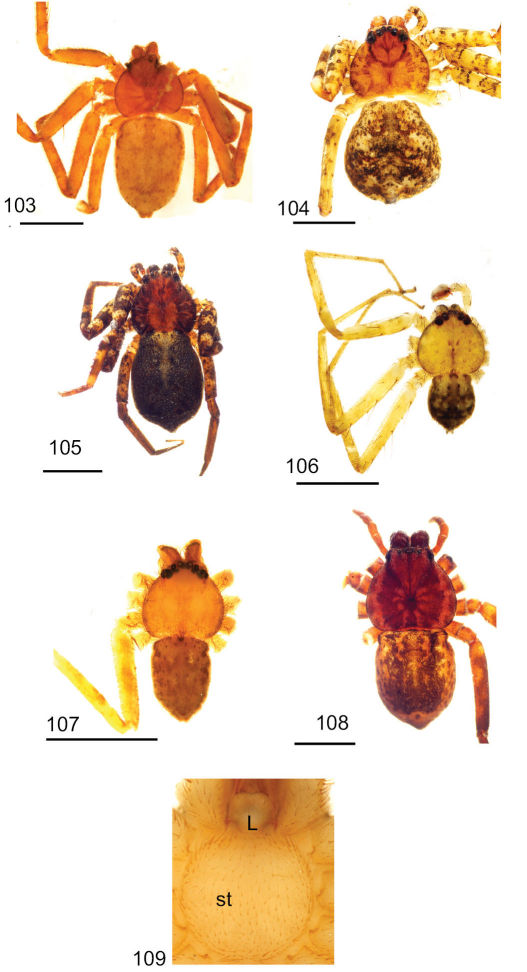
Habitus of representatives of various selenopid genera. **103** *Amamanganops baginawa* sp. n., female, from Mindoro, Philippines. The orange hue is likely not natural and seems to develop after being preserved for some time **104** *Anyphops barnardi* (Lawrence), female, from Gauteng South Africa **105** *Anyphops parvulus* (Pocock), female, from Tsitsikamma National Park, South Africa **106** *Garcorops madagascar* Corronca, male, from Hellville, Madagascar **107** *Godumops caritus* sp. n., male, Madang Province, Papua New Guinea **108** *Hovops* sp., female, from Park National Montagne d’Ambre, Madagascar **109** Labium of *Godumops caritus* sp. n. showing m-shaped distal region. Scale bar = 3.00 mm. Abbreviations: **L** = labium, **st** = sternum.

**Figures 110–115.**
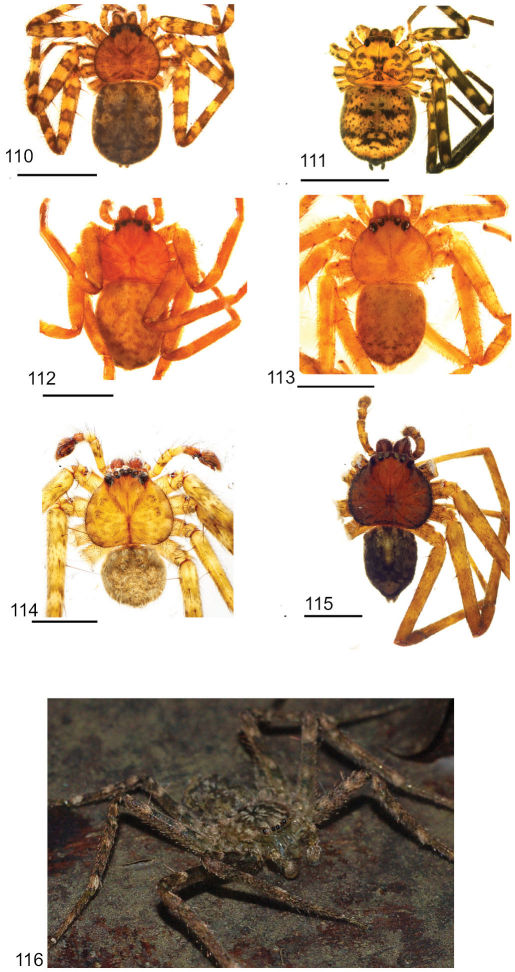
Habitus of representatives of various selenopid genera. **110** *Karaops ellenae* sp. n., female, from Mount Cooke, Western Australia, Australia **111** *Karaops raveni* sp. n., female, from Watchimbark Nature Reserve, New South Wales, Australia **112** *Makdiops montigenus* comb. n., female, from Dehra Dun, India. The orange hue is not natural and due to preservation **113** *Makdiops nilgirensis* comb. n., female, from Karteri Valley, Tamil Nadu, India. The yellow-orange hue is due to preservation **114 ***Selenops radiatus* Latreille, male, from Namibia **115** *Selenops phaselus* Muma, male, from Las Abejas, Dominican Republic **116** *Pakawops formosanus* (Kayashima), penultimate male, Taiwan (photo by Spideryang - http://www.flickr.com/photos/spideryang/3993100774/). Scale bar = 3.00 mm.

#### Genus Pakawops

gen. n.

urn:lsid:zoobank.org:act:8BA445B5-ADC4-46C4-8552-3809E8000BAA

http://species-id.net/wiki/Pakawops

##### Type species:

*Selenops formosanus* Kayashima, 1943a.

##### Etymology.

*Pakawops* gen. n. comes from a combination of words and honors the indigenous peoples of Taiwan and refers to the indigenous selenopid known to occur on the island. Though there were many different indigenous languages of Taiwan, we chose the extinct East Formosan Basay language, as the type locality is within area the Basay peoples once inhabited. Basay: *Pakaw =* spider; Greek: *ops* = face, eye. We retain the traditional ending of selenopid genera of *ops*, which originally referred to the eye arrangement. The gender is masculine.

##### Diagnosis.

*Pakawops* gen. n.can be separated from all other genera by the presence of 7 pairs of ventral spines on tibiae I and II, and 5 pairs on metatarsi I and II in combination with being found in Taiwan.

##### Remarks.

Although we have not examined specimens of this species, the published descriptions (Kayashima 1943a, b) show it to be clearly different from any other genus of Selenopidae. Kayashima (1943a) mentions that it is similar to *Selenops radiatus*, though it differs in the ventral tibial and metatarsal spination and is much smaller.

##### Description.

Total length 6.10. *Cephalothorax:* Carapace yellowish brown to grey wider than long; setae long and thin; chelicerae with 2 retrolateral teeth. *Legs:* Leg II longer than leg IV, leg III longest; tibial and metatarsal ventral spination 7–5.

##### Distribution.

Taiwan, near Taipei (Map 1). It is likely found on other parts of the island.

##### Composition.

A single species, *Pakawops formosanus* (Kayashima, 1943) comb. n.

##### Pakawops formosanus

(Kayashima, 1943) comb. n.

http://species-id.net/wiki/Pakawops_formosanus

Fig. 115 Map 1

Selenops formosanus Kayashima 1943a: 34, plate 17, fig. 2. Kayashima 1943b: 65.

###### Type material.

Kayashima’s collection was thought to have been left in Taiwan when he went to Malaysia (H. Ono pers. comm.), but the material has not been located.

##### Genus Siamspinops

Dankittipakul & Corronca, 2009

http://species-id.net/wiki/Siamspinops

Siamspinops Dankittipakul and Corronca 2009: 69. Type species: *Siamspinops spinosissimus*, Dankittipakul and Corronca, 2009, by original designation.

###### Diagnosis.

*Siamspinops* females can beseparated from those of all other genera by the combination of highly coiled spermathecae and by the presence of a posterodorsal epigynal fold. Some members of *Karaops* gen. n. also have strongly coiled ducts, but are lacking the posterodorsal fold. Males can be easily separated by having extremely strongly forward projecting chelicerae and long fangs. No other genus of selenopid has these characters.

###### Description.

Total length 6.00–7.90. *Cephalothorax:* Carapace with some dusky marks, wider than long. Short, broad, shallow fovea. Setae simple. AER straight, PER recurved. AME smaller than PME. Chelicerae slightly geniculate and robust in female; chelicerae and fangs in male are very long and strongly projecting forward; with 3 prolateral and 2 retrolateral teeth. *Legs:* Leg II is longer than leg IV, with leg III longest in females; tibiae I and II with 11–15 paired ventral spines, metatarsi I and II with 7–13 paired ventral spines; tarsal scopulae present. *Female copulatory organs:* Epigynum with or without lateral lobes, with median field and epigynal pockets. Spermathecae heavily sclerotized and coiled, with 7–14 spirals, posterodorsal fold present. *Male copulatory organs:* Palpal tibia with 2 tibial apophyses; embolus long and filiform; conductor T-shaped with one tip pointed;MA with only one branch, simple, and hook-shaped.

###### Distribution.

*Siamspinops* occurs in Southeast Asia from Thailand, south to Malaysia (Map 1).

###### Composition.

Four species, *Siamspinops allospinosus*, *Siamspinops spinescens*, *Siamspinops spinosissimus*, *Siamspinops spinosus* which were all recently described by Dankittipakul and Corronca (2009), and we transfer one species, *Selenops aculeatus*, to *Siamspinops*, and redescribe this species, bringing the total number of species to five. It is likely there are many more species in the region.

#### Siamspinops aculeatus

(Simon, 1901) comb. n.

http://species-id.net/wiki/Siamspinops_aculeatus

Figs 3–4 Map 1

Selenops aculeatus Simon 1901: 64.

##### Type material.

Female holotype (UMZC I.47430): Gunong, Malaysia [5°55'N, 102°20'E].

##### Diagnosis.

The female of this species can be easily distinguished from all others by the copulatory organs, as the sperm ducts are coiled 16 times, and a posterodorsal fold is present (Fig. 4). Male unknown.

##### Remarks.

*Selenops aculeatus* comb. n. is assigned to *Siamspinops* based on the morphology of the numerous spermathecal coils and the very spiny legs (Dankittipakul and Corronca 2009).

##### Description.

*Holotype:*Color: carapace uniformly yellow-brown; sternum pale yellow; chelicerae pale yellow with darker infuscations anteriorly and laterally; maxillae pale yellow-brown, lightening distally; labium pale yellow-brown, lightening distally; abdomen dorsally reddish-brown, possibly faded; ventrally pale reddish; legs orange-brown with annulations on femora, patella and tibia, darkening distally. Cephalothorax:setae long and thin; 0.9 times longer than broad; fovea longitudinal, broad, very shallow. Eyes:AER slightly recurved; PER recurved; PME larger than AME, PLE largest; eye group width 1.54; eye diameters, AME 0.14, ALE 0.08, PME 0.20, PLE 0.20; interdistances AME-ALE 0.32, PME-PLE 0.29, ALE-PLE 0.2, AME-PME 0.03; ocular quadrangle AME-AME 0.45, PME-PME 0.89; clypeus 0.12 high. Mouthparts:chelicerae with a few stout setae medially and anteriorly; lateral boss present, smooth; promargin with 3 teeth, retromargin with 2 teeth; maxillae longer than broad, with tuft of conspicuous setae distally; labium distally rounded. Sternum:0.99 times longer than broad, posteriorly indented. Pedipalp:tarsus slightly swollen, claw broken off. Legs:leg I much shorter than III, slightly shorter than IV; leg formula 3241; scopulae absent on all legs; tarsus I–IV with strong claw tufts; claws without teeth; spination: leg I, Fm pr 1–1–0, d 1–1–1, rl 0; Ti 2–2–2–2–2–2–2; Mt v 2–2–2–2–2; Ti and Mt I and II with strong spines; leg II, Fm pr 0, d 1–1–1, rl 0; Ti 2–2–2–2–2–2–2; Mt 2–2–2–2–2; leg III, Fm pr 0, d 1–1–1, rl 0; Ti 0; Mt 0; leg IV, Fm pr 0, d 1–1–1, rl 0; Ti 0; Mt 0. Abdomen:terminal setal tufts absent. Epigyne:lateral lobes indistinct, median ovoid area, copulatory openings located posterolaterally, epigynal pockets present; internally ducts coiled c. 15 times, posterodorsal fold present (Figs 3–4). *Dimensions:* Total length 6.68. Cephalothorax length 2.57, width 2.87. Sternum length 1.48, width 1.50. Abdomen length 3.95, width 2.86. Pedipalp: Fm 0.75, Pt 0.48, Ti 0.56, Ta 0.72, (total) 2.51. Leg I: Fm 2.62, Pt 1.12, Ti 2.31, Mt 1.73, Ta 0.92, (total) 8.70. Leg II: Fm 3.39, Pt 1.17, Ti 2.73, Mt 2.05, Ta 0.89, (total) 10.23. Leg III: Fm 3.82, Pt 1.09, Ti 2.90, Mt 2.18, Ta 1.01, (total) 11.00. Leg IV: Fm 3.29, Pt 1.06, Ti 2.29, Mt 1.74, Ta 0.91, (total) 9.29.

##### Natural history.

No data

##### Distribution.

The type locality only (Map 1).

## Supplementary Material

XML Treatment for Selenopidae
